# Astrocyte–microglia crosstalk in subarachnoid hemorrhage: mechanisms and treatments

**DOI:** 10.3389/fimmu.2025.1547858

**Published:** 2025-06-30

**Authors:** Kaibo Yu, Ding Wang, Wenhua Yu

**Affiliations:** ^1^ Department of Neurosurgery, The Second Affiliated Hospital, Zhejiang University School of Medicine, Hangzhou, China; ^2^ Department of Neurosurgery, Affiliated Hangzhou First People’s Hospital, School Of Medicine, Westlake University, Hangzhou, China

**Keywords:** subarachnoid hemorrhage, astrocyte, microglia, neuroinflammation, crosstalk

## Abstract

Subarachnoid hemorrhage (SAH) is a frequently encountered critical emergency characterized by the rupturing of an unhealthy blood vessel, resulting in high mortality and disability rates. Alterations in the neurovascular unit (NVU) are closely related to the pathogenesis of SAH. Microglia, the primary innate immune cells in the brain, and astrocytes, the most abundant cells in the brain, both play crucial roles in the response to SAH-associated cerebral injuries. Recently, the crosstalk between these two cells in the pathology and treatment of central nervous system (CNS) diseases, including SAH, has been revealed. Following acute brain insult, activated microglia and astrocytes can further activate each other, contributing to amplified neuroinflammatory reactions and thus inducing secondary brain injury. This review addresses the pathophysiological mechanisms of microglia and astrocytes in SAH, including neuroinflammation, neuronal damage, blood–brain barrier (BBB) disruption, vasospasm, and hematoma clearance. In addition, the newly identified therapeutic strategies against SAH by regulating astrocytes-microglia crosstalk through targeting damage-associated molecular patterns (DAMPs), immune mediators, and their receptors are also discussed. A thorough comprehension of microglia–astrocyte communication could provide novel ideas for future research and treatment of SAH.

## 1 Introduction

Subarachnoid hemorrhage (SAH) is a neurologic emergency caused by the rupture of a diseased vessel and following bleeding into the subarachnoid space. SAH ranks as the third most common type of stroke, with a mortality rate of almost 50%. Additionally, 30% of SAH survivors are unable to return to their previous way of life (1, 2). Hemorrhaging leads to a quick rise in intracranial pressure (ICP) and widespread cerebral ischemia, potentially resulting in death within minutes. Despite the availability of advanced interventional and microsurgical methods for securely closing aneurysms, patients who survive the initial period after SAH still face a high risk of morbidity and mortality (3). In addition to early brain injury (EBI) following SAH, delayed cerebral ischemia (DCI) is also closely related to poor outcomes in SAH patients. Increasing evidence suggests that microcirculatory dysfunction, glymphatic impairment, inflammation, and neuroelectric disruption are the main pathological factors of SAH-associated DCI (4).

**Figure 1:**
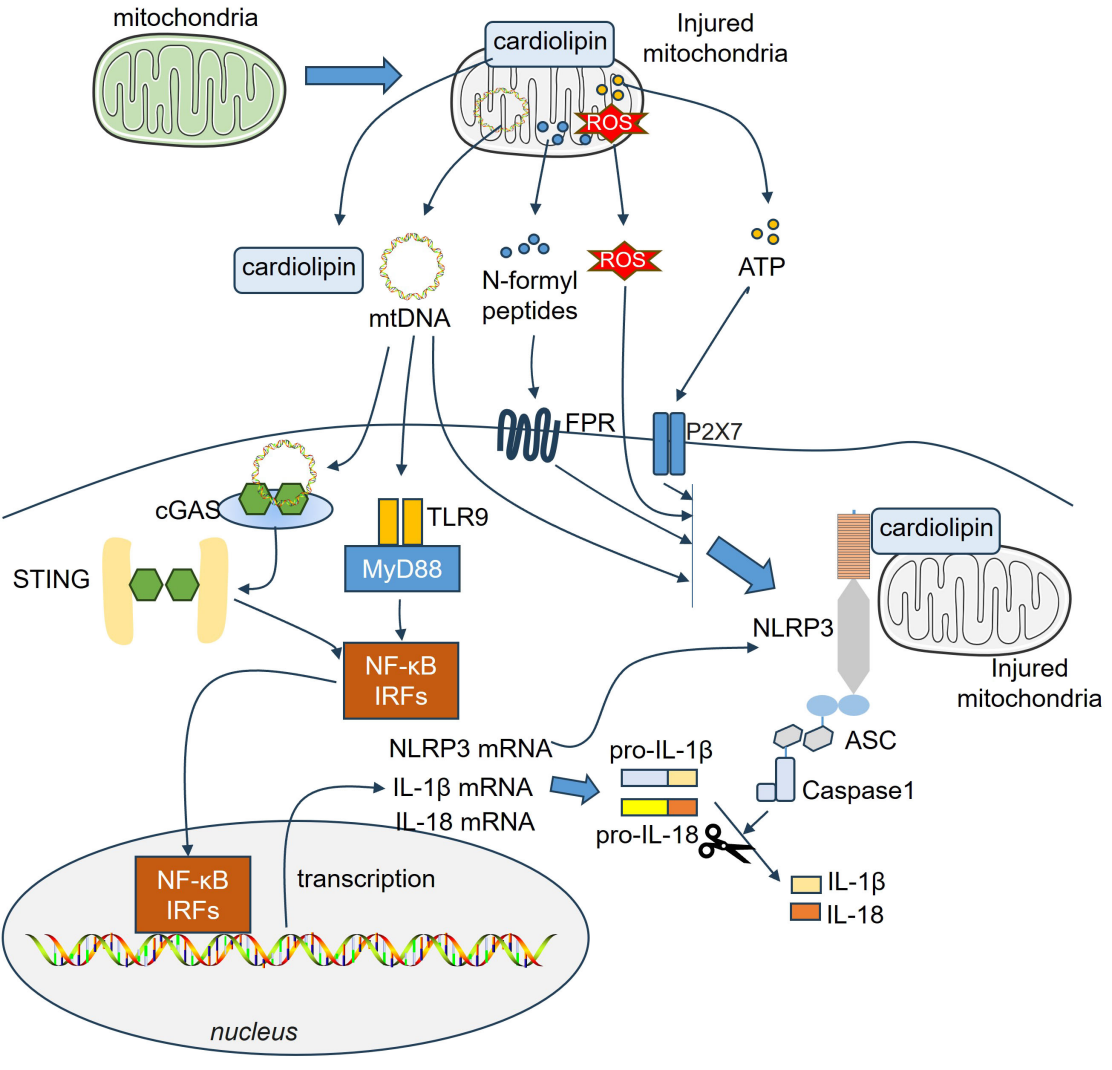
Astrocyte and microglia activation in the pathological changes of SAH. Following SAH, DAMPs are released from damaged cells, such as neurons, endothelial cells, RBCs. Those DAMPs stimulate astrocyte and microglia and induce their activation. Many mediators are involved in the interactions between activated astrocytes and microglia, which include cytokines, Ca2+, chemokines, ATP, growth factors, and so on. ATP, adenosine triphosphate; BBB, blood–brain barrier; BDNF, brain-derived neurotrophic factor; DAMPs, damage-associated molecular patterns; FGF2, fibroblast growth factor 2; GDNF, glial-derived neurotrophic factor; SAH, subarachnoid hemorrhage.

Maintaining the neurovascular unit (NVU) requires normal molecular crosstalk between the nervous and vascular systems (5). Following SAH, there is a broad spectrum of mild to severe neurovascular dysfunctions, including blood–brain barrier (BBB) damage, astrocytic and microglial activation, leukocyte infiltration, vasoconstriction, and astrocyte endfoot hypertrophy (6) (**Figure 1**). BBB injury disturbs the crosstalk among endothelial, vascular, glial, neural, and immune cells and leads to EBI and DCI (6, 7). During the first few hours after SAH, neurons, astrocytes, and parenchymal arterioles maintain normal communication (8). However, progressive impairment of NVC emerges after cerebral hemorrhage, which is considered a secondary pathological alteration (9). Astrocytes, the most abundant cells in brain, surround brain ECs with their endfeet and maintain BBB integrity, cerebral blood flow, nutrient uptake, and waste clearance. They also regulate immune reactions and support BBB integrity (10, 11). However, SAH-mediated astrocytic activation contributes to altered BBB permeability by producing neurotoxic mediators (12, 13). SAH induces changes in calcium (Ca^2+^) signaling within astrocytes, and astrocytic endfeet exhibit asymmetrical hypertrophy, resulting in a change in the neurovascular coupling response where vasodilation shifts to vasoconstriction. suggesting that astrocytes play an essential role in SAH-induced decreases in cortical blood flow (14). However, astrocytes also exert protective functions in SAH by mitigating endothelial cell (EC) dysfunction and BBB permeability (13).

Microglia are immune cells that originate from leptomeningeal mesenchymal cells. Under the stimulation of damage-associated molecular patterns (DAMPs) after SAH, microglia quickly become activated and polarized into different states (15). Classically activated microglia (also called “M1” microglia) can induce secondary brain injury by producing cytotoxic effectors, which aggravate neuronal damage and BBB disruption (16, 17). During the delayed phase of SAH, microglia dynamically polarize from the M1 to the M2 phenotype (18). M2 polarization of microglia significantly improves the inflammatory response, oxidative damage, neuronal degeneration, and BBB breakdown following SAH (19–21). Thus, dual roles are mediated by microglia in controlling BBB integrity. On the other hand, various studies have suggested that microglia–astrocyte crosstalk plays crucial roles in neurodevelopment, homeostasis, and central nervous system (CNS) disease progression (22, 23). Microglia react faster to pathological stimuli than astrocytes and then induce active astrocytes. However, astrocytes can also affect the activation of microglia (7). Following acute brain insult, activated microglia and astrocytes can further activate each other, contributing to amplified neuroinflammatory reactions and thus inducing secondary brain injury (24).

In our previous studies, we have reported that microglia and astrocytes exhibit swift reactions following stroke. Excessive reactive oxygen species (ROS) are generated via the mitochondrial and NADPH oxidase pathways, contributing to oxidative damage to microglia, astrocytes, and neurons (25). Many immune mediators, such as cytokines, chemokines, adenosine triphosphate (ATP), matrix metalloproteinases (MMPs), and growth factors, are altered following SAH (**Table 1**). They are essential for communication between microglia and astrocytes, thus maintaining brain homeostasis and mediating neuropathologies during different stages of SAH.

**Table 1:** The expressions and clinical associations of immune mediators in SAH patients.

Mediators	Source	Grouping		Expression	Clinical relevances	Ref.
IL-2, IL-6, IL-8, IL-10, and TNF-α	serum	poor prognosis group	good prognosis group	up	IL-2, IL-6, IL-8, and IL-10 levels were positively correlated with mRS scores	(26)
IL-4	serum and CSF	aSAH patients	healthy people	up	poor outcome, age, platelet-lymphocyte ratio (PLR), C-reactive protein (CRP), Hunt-Hess grade, mRS score, and World Federation of Neurological Surgeons score (WFNS), complications (intracranial infection, cerebral edema, hydrocephalus, and complications by DCI.	(27)
IL-6, CCL2, CCL11, CSF3, IL-8, IL-10, CX3CL1, and TNF-α	serum	higher clinical severity	lower clinical severity	up	Platelet-derived growth factor (PDGF)-AA, PDGF-AB/BB, soluble CD40 ligand, and tumor necrosis factor-α (TNF-α) increased over time. Colony-stimulating factor (CSF) 3, interleukin (IL)-13, and FMS-like tyrosine kinase 3 ligand decreased over time. IL-6, IL-5, and IL-15 peaked and decreased	(28)
IL-23 and IL-17	serum	aSAH patients	control patients	up	IL-23 and IL-17 showed differential correlations with post hemorrhagic complications	(29)
IL-1α, IL-18, IL-6 and IL-8	CSF	aSAH patients	control patients	up	IL-4 was higher in the CSF of patients who had delayed ischaemic neurological deficit. Day 3 plasma IL-6 levels predicted poor outcome at six months	(30)
IL-17	CSF	aSAH patients	control patients	down		
IL-6 and IL-8	Plasma	aSAH patients	control patients	up		
IL-1α	Plasma	aSAH patients	control patients	down		
CCL2, CCL4, CCL7, CCL11, CCL13, CCL19, CCL20, CXCL1, CXCL5, CXCL6 and CXCL8	CSF	poor outcome patients	good outcome patients	up	Higher levels of CCL11, CCL25, CXCL5 are associated with WFNS score, Fisher score or occurrence of delayed cerebral ischemia (DCI)/delayed ischemic neurological deficit (DIND)	(31)
CCL5	CSF and serum	aSAH patients	control patients	up	CSF CCL5 levels on post-aSAH day 1 were correlated with poor clinical outcome, however, serum CCL5 levels on post-aSAH day 7 were correlated with good clinical outcome.	(32)
CXCL12	serum	aSAH patients	control patients	up	Elevation of serum CXCL12 levels is associated highly with hemorrhagic severity and poor outcome after aSAH	(33)
FABP3 and CXCL-16	serum	poor outcome patients	good outcome patients	up	Early FABP3 and CXCL-16 levels are significantly associated with poor 30-day outcome in patients with aSAH	(34)
CRP	serum and CSF	poor outcome patients	good outcome patients	up	Patients with angiographic vasospasm had higher CRP measurements in serum and CSF	(35)
TNF-α	serum	poor outcome patients	good outcome patients	up	TNF-α over time are associated with poor outcome	(36)
HsCRP	blood	with vasospasm	without vasospasm	up	High plasma levels of HsCRP were significantly associated with angiographic vasospasm and clinical outcome	(37)
		poor outcome patients	good outcome patients	up		
E-selectin , ICAM-1, and VCAM-1	CSF	aSAH patients	control patients	up	Levels of E-selectin were associated with patients who later developed moderate or severe vasospasm	(38)
MMP-9	blood and CSF	poor outcome patients	good outcome patients	up	blood and CSF MMP-9 are associated with poor 3-month SAH clinical outcome	(39)
IL-33	Serum	aSAH patients	control patients	up	High serum IL-33 concentrations have close relation to the inflammation, severity and poor outcome in aSAH	(40)
TGF-β1	CSF	SAH	control patients	up	TGF-beta1 in CSF after SAH are derived initially from blood and later from endogenous sources such as the choroid plexus	(41)
Factor III, Factor VII, fibrin,TAT, IL-1β , IL-2, IL-5, IL-6, IL-7, IL-8, IL-12, IL-17, G-CSF, GM-CSF, IFN-γ, CCL-2 , CCL-4, and TNF-α	CSF	severe cerebral edema	mild cerebral edema	up	TAT, fibrin, IL-1β, IL-2, IL-5, IL-7, and IL-4 were independently associated with severe cerebral edema; Factor VII, fibrin, IL-2, IL-5, IL-12, TNF-α, and CCL-4 were independently associated with chronic hydrocephalus	(42)

## 2 Dynamics of microglia/astrocyte activation in SAH

### 2.1 Dynamics of microglia activation in SAH

Microglia undergo a dynamic activation following SAH with spatiotemporal characteristics. At the early stage of SAH (0-3 days), microglia are rapidly activated in response to the presence of blood components and DAMPs. They change their morphology from a ramified, resting state to an amoeboid, activated state (43). Activated microglia can release a variety of pro-inflammatory cytokines and chemokines, including tumor necrosis factor-alpha (TNF-α), interleukin-1 beta (IL-1β), and interleukin-6 (IL-6). These molecules recruit immune cells from the periphery and initiate an inflammatory cascade, which can contribute to early brain injury (44). The NLRP3 (NOD-, LRR- and pyrin domain-containing 3)-ASC (apoptosis-associated speck-like protein containing a CARD) inflammasome activation plays an essential role in microglia activation, which mediates enhanced cytokine and chemokine concentrations. Within the early stage of SAH, the NLRP3-ASC inflammasome was increased in a time-dependent manner and peaked at 24 h after SAH (45).

Research on mice and rats revealed that microglia exhibited a phenotypic shift during the intermediate stage (3-5 days). They can adopt a more M2-like inflammatory phenotype, which is characterized by the release of cytokines such as interleukin-4 (IL-4) and transforming growth factor-beta (TGF-β). Microglia change morphologically from a ramified to an amoeboid form. Those shifts are thought to be an attempt to resolve inflammation and promote tissue repair (18, 46, 47). During the late stage (5 days and later), microglia begin to phagocytose red blood cells, cell debris, and other foreign substances in the subarachnoid space. This process helps to clear the area of damaged tissue and potentially harmful substances (48). Iba1-labeled microglia/macrophages were significantly enhanced at 7-day and 14-day post-SAH, with elevated neuronal cell death (49). Though “M1” microglia are reduced in the perforated site and hippocampus during the intermediate stage, they are then enhanced at 10 days after SAH (18). Elevated productions of IL-6 and TNF-α can be detected 1 and 2 months after SAH, suggesting that chronic inflammation can persist. They may lead to further damage and potential long-term neurological deficits (49).

### 2.2 Dynamics of astrocyte activation in SAH

Astrocytes significantly contribute to the integrity of the BBB. Their endfeet surround the microvessel walls of the BBB, maintaining a structure that is essential for proper BBB function (50). GFAP-labeled astrocytes are activated 6 hours after SAH in the ventral cortex, and their activation in the dorsal cortex can be observed 24 hours after SAH (51). They change from a resting state to an activated state and show increased cell sizes and an upregulation of MMP9, which mediates BBB disruption (52). Over the course of the following week (days 1-7), MMP9 upregulation became detectable, starting from the cortical top layer towards the deeper layers (52). GFAP levels in the cerebrospinal fluid (CSF) from SAH patients were significantly enhanced and reached the highest on day 1 post-SAH. Higher GFAP levels were associated with poorer clinical outcomes of SAH patients (53). Astrocytes participate in the inflammatory response after SAH by secreting both proinflammatory and anti-inflammatory factors after the activation of distinct pathways. Neuroinflammation after SAH was very long-lasting and still present at day 21 (54–56). In the subacute and chronic stages, astrocytes gradually form a glial scar around the damaged area. The glial scar is mainly composed of astrocyte-derived extracellular matrix components and serves as a physical barrier to prevent the spread of inflammation and limit the invasion of immune cells and harmful substances. However, the glial scar may also hinder the regeneration of nerve fibers and affect the recovery of nerve function (57, 58).

## 3 DAMPs in microglia/astrocyte activation in SAH

Following tissue injury, host nuclear or cytoplasmic non-microbial molecules are released from cells, which are known as DAMPs. These DAMPs trigger the immune system, leading to a non-infectious inflammatory response that can result in systemic inflammation, organ damage, and potentially death (59). A wide variety of endogenous molecules released upon brain injury are termed DAMPs, which cause the activation of microglia and astrocytes (60). For example, extracellular S100B (61) and high mobility group box 1 (HMGB1) (62) are dramatically elevated after brain injury and contribute to reactive gliosis in the injured brain through the activation of a receptor for advanced glycation end products (RAGE) or toll-like receptor (TLR)4 signaling. Other DAMPs, such as heat shock proteins (HSPs), adenosine triphosphate (ATP), purines, and peroxiredoxins, also play vital roles in the activation of the immune system early following brain insult, as reviewed in previous studies (63–66). The increased production of DAMPs in the serum or CSF following SAH is closely associated with the outcomes of SAH patients, and those DAMPs are promising diagnostic, prognostic, therapeutic, and drug therapy candidates for SAH (67) (**Table 2**). Notably, these DAMPs induce significant activation of microglia and/or astrocytes (**Table 3**), leading to further aggravated neurovascular dysfunctions in SAH.

**Table 2:** The expressions and clinical associations of DAMPs in SAH patients.

Mediators	Source	Grouping		Expression	Clinical relevances	Ref.
ex-mito	CSF	aSAH patients	control patients	up	higher mitochondrial membrane potentials in the CSF were correlated with good clinical recovery	(68)
mito-DNA	Serum	aSAH patients	control patients	up	mtDNA may directly or indirectly influence post-SAH complications and clinical outcome	(69)
	CSF and serum	aSAH patients	healthy people	up	Higher CSF DNA levels are associated with worse outcomes in patients with acute spontaneous aneurysmal SAH	(70)
HMGB1	CSF	SAH patients	control patients	up	Altered concentration of CSF HMGB1 correlates with outcome	(71)
	CSF	aSAH patients	control patients	up	CSF HMGB1 levels were positively associated with disease severity scores, IL-6 levels, DCI and 6-month poor outcome	(72)
	CSF	poor outcome	good outcome	up	CSF HMGB1 level was independently associated with unfavorable outcome at three months post-SAH	(73)
	Plasma	aSAH patients	control patients	up	plasma HMGB1 level was an independent predictor of poor functional outcome and mortality after 1 year, in-hospital mortality and cerebrovasospasm	(74)
	Serum	ICH patients	normal controls	up	Serum HMGB1 levels were significantly correlated with the levels of IL-6 and TNF-α and poor outcome of ICH patients	(75)
S100B	CSF and serum	poor outcome	good outcome	up	Serum S100B levels were significantly correlated with poor outcome of SAH patients	(76)
	CSF and serum	poor outcome	good outcome	up	CSF and Serum S100B levels were associated with poor outcome, intracranial hypertension and cerebral infarction of spontaneous SAH patients	(77)
	Serum	severe EBI	mild to moderate EBI		Early S100B seems to have a good diagnostic value to predict severe EBI.	(78)
	Serum	aSAH patients	healthy controls	up	Serum S100B levels predict the clinical prognosis of patients with aSAH	(79)
hemoglobin	Serum	poor outcome	good outcome	down	Higher HGB values are associated with improved outcomes after SAH at 14 days/discharge and 3 months.	(80)
	Serum	poor outcome	good outcome	down	Lower hemoglobin levels are associated with worse outcomes	(81)
	Serum	poor outcome	good outcome	down	SAH patients with higher initial and mean hgb values had improved outcomes	(82)
S100A8/A9	Serum	aSAH patients	healthy controls	up	Serum S100A8/A9 levels within 48 hours after onset was significantly correlated with poor outcome of aSAH patients	(83)
S100A12	Serum	poor outcome	good outcome	up	High serum S100A12 levels at admission are associated with inflammatory response and poor outcome of ICH patients	(84)
hemopexin	Serum	SAH patients	control patients	up	Higher hemopexin levels were associated delayed cerebral ischemia and poorer neurological outcome	(85)
ferritin	Serum	SAH patients	control patients	up	CSF ferritin level after SAH might be a new diagnostic marker	(86)

**Table 3:** The roles of DAMPs in microglia-astrocyte interactions.

DAMPs	Mechanisms		Ref.
	Microglia	Astrocyte	
HMGB1	HMGB1 enhances IL-1β expression from microglia	HMGB1 upregulates AQP4 expression with the help of microglia-derived IL-1β	(87)
	HMGB1 induces NF-κB activation by TLR2, TLR4, and RAGE	HMGB1 induces NF-κB activation in astrocytes with microglial cooperation	(88)
	HMGB1-induced release of CCL5 from astrocytes promotes microglia/macrophage accumulation and M1 polarization.	HMGB1 facilitates the production of CCL5 from astrocytes by binding with TLR2/4 receptors potently	(89)
hemoglobin	Hemoglobin promotes the expression of cytokines (ex. TNF-α, IL-1β, and IL-6) from microglia	Oxyhemoglobin activate the NF-κB pathway in astrocytes and increases the production of proinflammatory cytokines (including TNF-α, IL-1β, IL-6 and MMP9)	(90–94)
mitochondrial DNA	mtDNA promotes “M1” polarization of microglia via the activation of the STING and IRF3/NF-κB signaling pathways	Copper exposure caused the release of mtDNA and subsequent cGAS-STING pathway activation	(95, 96)
S100B	S100B stimulates microglial migration via the upregulation of chemokines and chemokine receptors and promotes proinflammatory reactions of microglia by S100B/RAGE-dependent activation of the JNK and NF-κB pathways	S100B promotes the NF-κB pathway activation dose-dependently and upregulates endogenous RAGE in astrocytes	(83, 97, 98)
Peroxiredoxin6	The peroxiredoxins 6 (PRDX6)-phospholipase A2 (iPLA2) axis is involved in the activation of microglia	PRDX6 is mainly expressed and produced by astrocytes	(99, 100)

### 3.1 HMGB1

HMGB1 is a nonhistone DNA-binding protein. In the brain, HMGB1 mediates neurite outgrowth and cell migration with a complex temporal and spatial distribution pattern (101). Clinical analysis revealed that the levels of HMGB1, along with those of IL-6 and TNF-α, are significantly increased in the cerebrospinal fluid (CSF) of patients with SAH (102) and in the serum of patients with intracerebral hemorrhage (ICH) (75). Notably, HMGB1 is positively correlated with these proinflammatory cytokines (75, 102). Under pathological conditions, such as cerebral ischemic stroke, HMGB1 can be released from injured neurons (103). As early as 2 h after experimental SAH, the mRNA and protein levels of HMGB1 were elevated. HMGB1 is translocated from the nucleus to the cytoplasm, which occurs mainly in neurons and, to a lesser extent, in microglia (104). In the hemoglobin (Hb)-induced *in vitro* SAH model, HMGB1 is rapidly produced from neurons in the medium (104). Murakami et al. reported that in the SAH model, more than 90% of HMGB1-expressing cells were IBA1-labeled microglia/macrophages, suggesting that microglia/macrophages are potentially the major source of HMGB1 in SAH (105). HMGB1 can also be produced by pyroptotic endothelial cells in brain lesions, which further enhances macrophage pyroptosis, resulting in immune disorders (106). In the microcirculation of the brain, HMGB1 expression is upregulated on platelet microvesicles from acute ischemic stroke (AIS) patients and induces neutrophil extracellular trap (NET) formation. The latter significantly promoted procoagulant activity through tissue factor and platelet activation (107).

**Figure 2:**
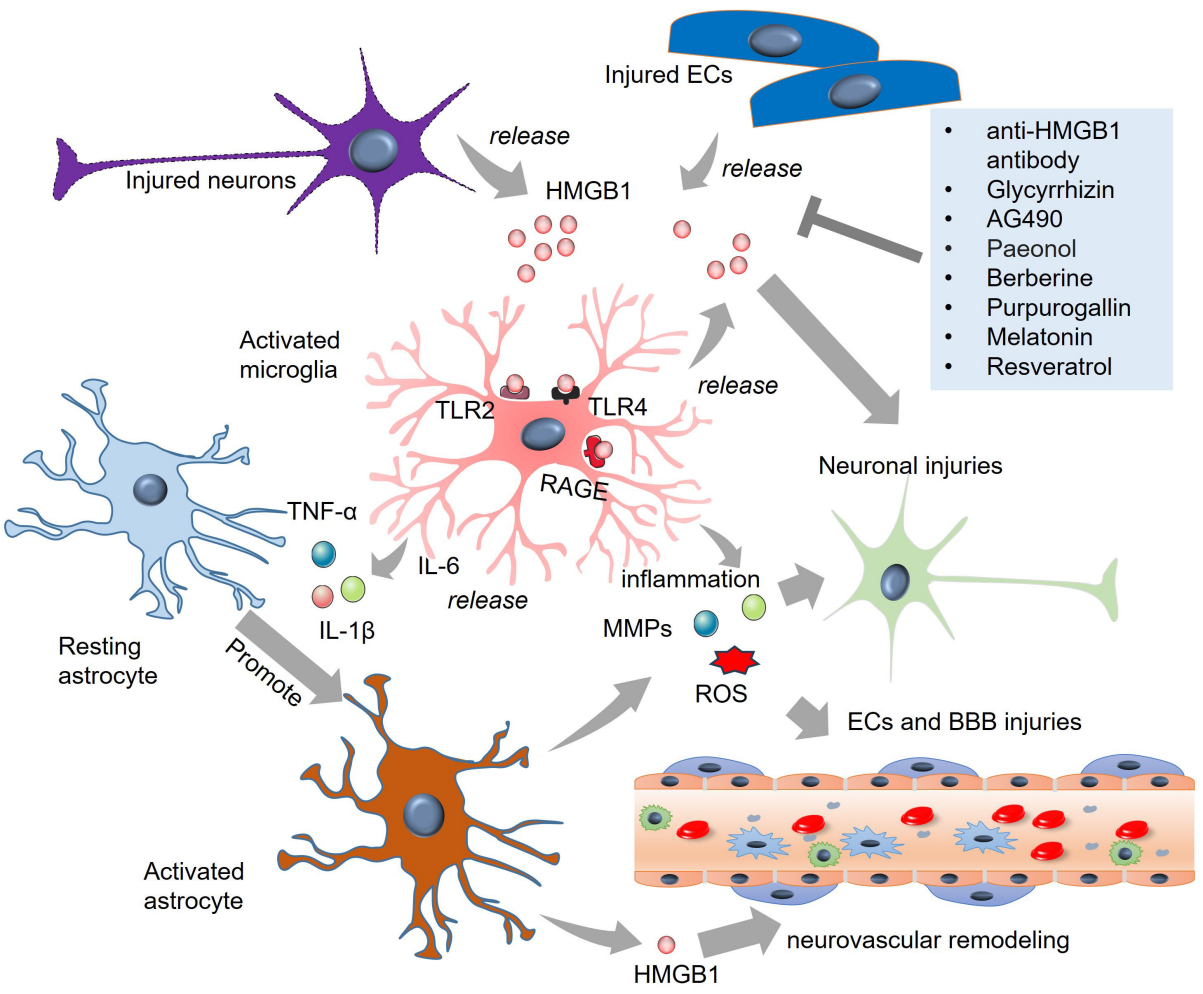
The role of HMGB1 in microglial and astrocytic activation in SAH. HMGB1 is released from injured neurons and endothelial cells. HMGB1 binds TLR-2, TLR-4, and RAGE on microglia and astrocytes and induces the activation of the two cells. Inflammatory cytokines, such as TNF-α, IL-6, and IL-1β, MMPs, and ROS are produced from activated microglia and astrocytes, which promote secondary injuries to neurons and endothelial cells. Targeting HMGB1 via multiple strategies, such as anti-HMGB1 antibody and glycyrrhizin treatment, can mitigate HMGB1-mediated microglial and astrocytic activation. BBB, blood-brain barrier; ECs, endothelial cells; HMGB1, high-mobility group box-1; IL-6, interleukin-6; RAGE, receptor for advanced glycation end products; ROS, reactive oxygen species; TLR, toll-like receptor; TNF-α, tumour necrosis factor-alpha.

HMGB1 plays a vital role in microglial and astrocyte activation in SAH (**Figure 2**). Extracellular HMGB1 binds several pattern recognition receptors (PRRs). The most important receptors include TLR-2, TLR-4, and RAGE on immune cells to increase inflammation (108). RAGE is expressed mainly by neurons and microglia rather than astrocytes after SAH (109). TLR2 and TLR4 are mainly expressed in microglia after SAH (110, 111). HMGB1 release is elevated in traumatic brain injury (TBI) and mediates TLR4 signaling activation in microglia. Activated microglia produce IL-6, which enhances astrocytic expression of aquaporin-4 (AQP4) and ultimately contributes to posttraumatic brain edema (62). Direct treatment of primary cultured astrocytes from rats with HMGB1 did not increase the level of AQP4 mRNA, nor did it affect the nuclear transfer of NF-κB in astrocytes. With the help of microglia-derived IL-1β, AQP4 expression is upregulated in astrocytes (87), suggesting that HMGB1 can mediate astrocytic activation with the help of proinflammatory cytokines produced by activated microglia. In the absence of glial cells, HMGB1 failed to induce neurodegeneration in primary cultured cortical neurons. Glycyrrhizin-mediated HMGB1 blockade reduces neuronal degeneration, reactive astrogliosis, and microgliosis in the long term (88). Recently, Chi et al. reported that in spinal cord injury (SCI), HMGB1 promoted chemokine (C-C motif) ligand 5 (CCL5) expression and release from astrocytes by binding to TLR2/4 receptors. CCL5 mediates microglia/macrophage accumulation and M1 polarization in spinal lesions (89). These studies revealed that the detrimental effects of HMGB1 depend on microglial–astrocytic interactions.

### 3.2 Hemoglobin/heme

Upon hemolysis, red blood cells (RBCs) release hemoglobin (Hb) into the circulation. Different Hb redox states and heme can act as DAMPs in the body (112). On the basis of clinical data, elevated Hb levels are associated with the clinical outcomes of SAH patients and predict less cerebral infarction, mortality, and vasospasm (80, 82, 113). However, increased Hb levels in the CSF of SAH patients are positively related to SAH-associated secondary brain injury (SBI), possibly by inducing an adaptive macrophage response and promoting vasoconstrictive and lipid peroxidation activities (114). When released from globin, heme is thought to be more toxic than hemoglobin, since it is highly lipophilic, easily intercalating into membranes and perturbing cellular function (115). Higher heme levels in the CSF have been found in SAH patients, with a positive association with the development of vascular injury and cerebral vasospasm (116).

Hb easily passes through the walls of cerebral arteries and the cortex, and there is a noticeable spatial relationship between blood clots and the accumulation of iron in the brain cortex (114, 117). Extracellular Hb is believed to have a crucial impact primarily on pathways associated with oxidative stress, inflammation, iron toxicity, and nitric oxide (118). Extracellular Hb/heme induces diverse toxic effects on neurons (119), pericytes (120), cerebral endothelial cells (121), and astrocytes (122). Compared with astrocytes, neurons are more susceptible to both hemoglobin and heme toxicity (123).

**Figure 3:**
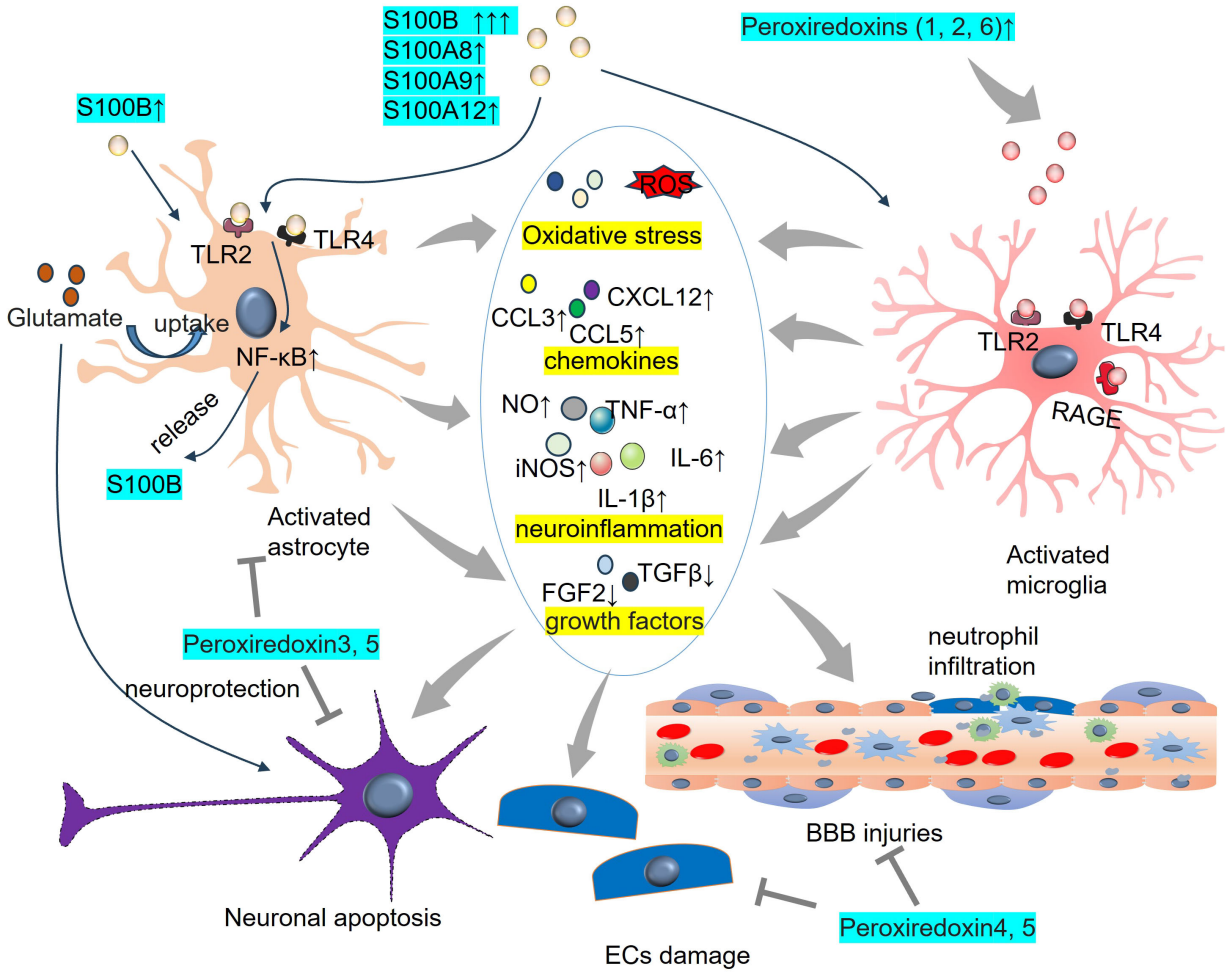
The role of Hb in microglial and astrocytic activation in SAH. Following SAH, hemoglobin (Hb) is released from erythrocytes upon hemolysis. Following dimerization and oxidation, heme is produced and released into the brain parenchyma. Both Hb and heme can mediate microglia/astrocyte activation and induce endothelial cell and BBB damage. BBB, blood-brain barrier; Hb, hemoglobin; NO, nitric oxide.

Accumulating evidence has shown that Hb/heme can mediate the microglial proinflammatory response by promoting the expression of cytokines (ex. TNF-α, IL-1β, and IL-6) and activate autophagy (90–92) (**Figure 3**). Microglia also have protective effects on ICH by accelerating hematoma clearance (80). For example, Hb promoted IL-10 expression in microglia and enhanced phagocytosis, which was dependent on the IL-10-regulated expression of CD36. The mice with IL-10 deficiency presented aggravated neuroinflammation, brain edema, iron deposition, and neurological deficits. The phagocytic ability of microglia is dampened due to decreased CD36 expression, leading to delayed hematoma clearance (124). CD163, a hemoglobin/haptoglobin scavenger receptor, can bind the hemoglobin-haptoglobin complex, thereby mediating extravasal hemolysis. CD163 also has potent anti-inflammatory effects on microglia/macrophages (125, 126). The absence of CD163 in the ICH mouse model resulted in reduced Hb, iron, and blood–brain barrier (BBB) disruption; increased astrogliosis; and neovascularization at 3 days. However, CD163 deficiency causes delayed injurious effects, as evidenced by enhanced iron and VEGF immunoreactivity (127). Damaged neurons can produce fractalkine (FKN) around the hematoma. FKN improves microglial erythrophagocytosis via the CD163/heme oxygenase-1 (HO-1) axis, thus mitigating neuroinflammation, hematoma size and Hb content and relieving neurological deficits in ICH mice (128, 129).

Astrocytes are involved in Hb- and haemin-mediated disorders during ICH (94, 130) (**Figure 3**). Hb pretreatment in astrocytes enhances the expression and nuclear translocation of Nrf2, thus preventing hemin-induced oxidative stress and apoptosis (131). However, Hb can promote pyroptosis and tissue factor production/release in primary astrocytes in SAH rats, which can be blocked by the caspase-1 inhibitor VX-765 (132). Oxyhemoglobin (OxyHb) promoted CD24 expression in hippocampal astrocytes, whereas knockdown of astrocytic CD24 contributed to impaired axons and dendrites of neurons cocultured with astrocytes (133). Astrocyte-derived glutathione (GSH) alleviated hemin-induced apoptosis in cerebral microvascular cells (134). Promoting the activation of Wnt5a/Ror2 signaling in astrocytes reduces heme-induced BBB damage after brain hemorrhage, and the underlying mechanism may depend on the nuclear accumulation of Nrf2 (135). Astrocytes can also affect neuroinflammation in ICH models. After exposure to OxyHb, astrocytes activate the NF-κB pathway and increase the production of proinflammatory cytokines (including TNF-α, IL-1β, IL-6 and MMP9) (93, 94). These effects were significantly reversed following the depletion of Nrf2 (93).

Excessive accumulated iron has been regarded as a hallmark of SAH-associated pathological changes. Iron-related brain injury is considered a major mechanism of intracranial hemorrhage as well as TBI (136). Recycling heme iron is an essential part of overall iron metabolism. Iron is freed from heme molecules with the help of enzymes called heme oxygenases (HOs), particularly HO-1 (137). Iron-rich red blood cells accumulate and lyse at the brain parenchyma following hemorrhagic stroke, thereby contributing to iron-induced lipid peroxidation and cell death (138). Iron deposition in the brain could also possibly modulate microglial activation and motility (139). Hepcidin is vital for iron homeostasis in the brain during neuronal iron loading and brain hemorrhage (140). After SAH, there was an exacerbation in iron accumulation, as well as a decrease in ferroportin 1 (FPN1) in neurons and an increase in hepcidin in astrocytes. Downregulating astrocytic hepcidin enhances neuronal FPN1 levels and reduces iron accumulation (141). Elevated levels of hepcidin-25 were discovered in the serum and mainly in astrocytes following ICH. In mice with ICH, the absence of hepcidin helped reduce the release of brain iron, oxidative brain damage, and cognitive deficits. The TLR4/MyD88 signaling promotes hepcidin expression via the IL-6-signal transducer and activator of transcription 3 (STAT3) signaling pathway, suggesting that inflammation plays an essential role in mediating astrocytic hepcidin expression in the brain (142).

### 3.3 Damaged mitochondria, N-formyl peptides, and mitochondrial DNA

**Figure 4:**
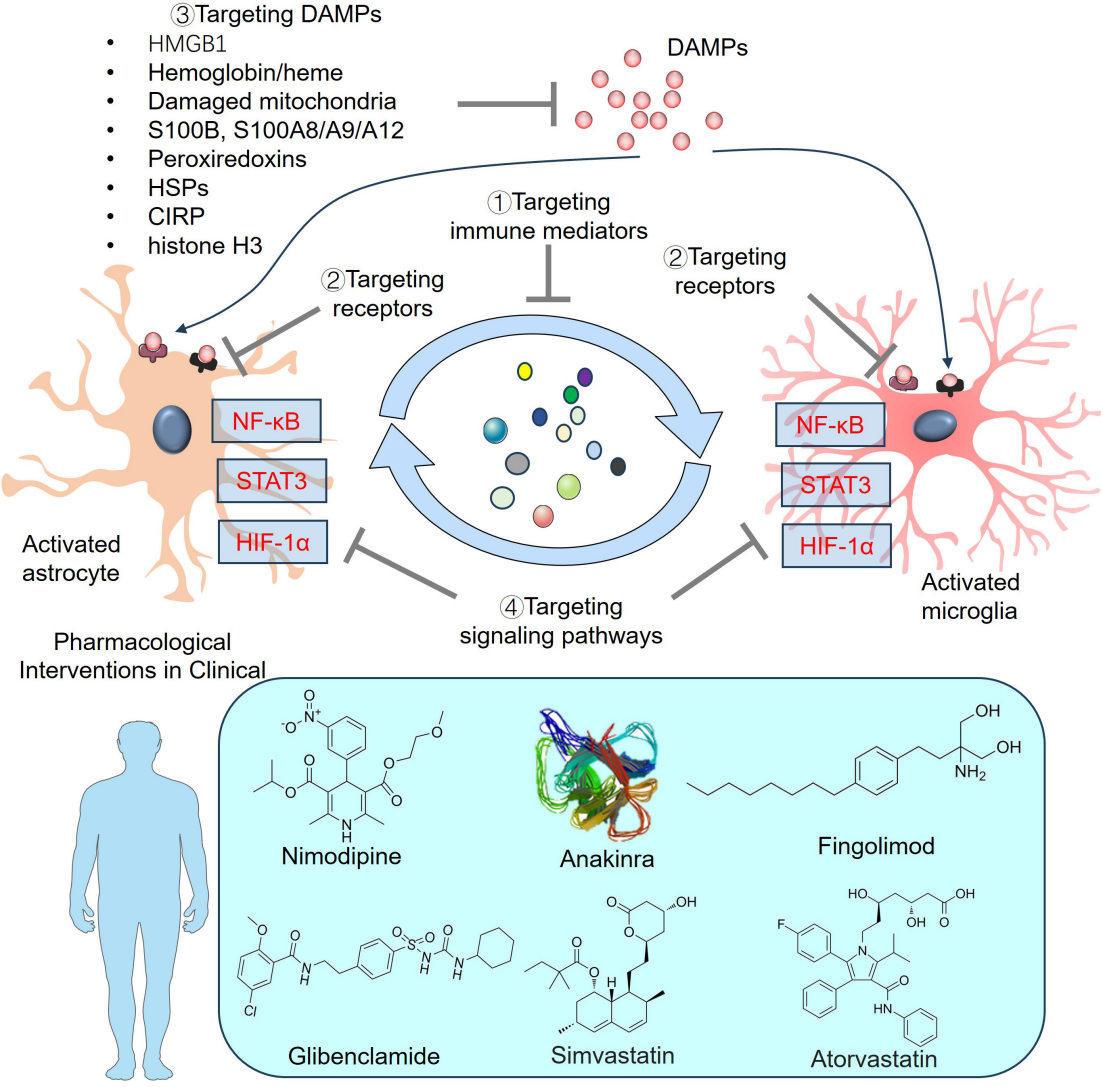
Mitochondria-related DAMPs on microglial and astrocytic activation. Endogenous mitochondrial-derived DAMPs, including N-formyl peptides, cardiolipin (CL), mtDNA and ATP are released from injured cells, which then bind different receptors and mediate inflammatory reactions. The downstream pathways, such as NF-κB, IRFs, NLRP3-ASC-Caspase1 inflammasome, become activated in astrocytes and microglia. ASC, apoptosis-associated speck-like protein a CARD; ATP, adenosine triphosphate; IRFs, Interferon regulatory factors; NF-κB, nuclear factor-kappaB; NLRP3, NACHT, LRR, and PYD domains-containing protein 3; ROS, reactive oxygen species.

Accumulating evidence has shown that damaged mitochondria, or the release of N-formyl peptides and mitochondrial DNA (mtDNA) from mitochondria, can act as DAMPs that activate the innate immune system (143–145) (**Figure 4**). Mitochondrial N-formyl peptides are similar to bacterial N-formylated peptides, both serving as strong immune system activators. Hemorrhagic shock increased the plasma levels of mitochondrial N-formyl peptides in patients with lung damage. The antagonism of formyl peptide receptors (FPRs) ameliorated hemorrhagic shock-induced lung injury in rats (146). Endogenous mitochondrial-derived DAMPs (MTDs), including N-formyl peptides, cardiolipin (CL), and mtDNA, are used to treat HMC3 cells to test their effects on microglial activation *in vitro*. These MTDs fail to induce microglial activation toward a proinflammatory phenotype. However, mtDNA and CL markedly increase reactive oxygen species production in microglia (147). Circulating mitochondrial N-formyl peptides, which are endogenous ligands of FPR1, are augmented and correlated with the magnitude of brain edema in ICH patients. FPR1 is the most abundant DAMP receptor and is expressed mainly by microglia. Interactions of formyl peptides with FPR1-activated microglia increase neutrophil recruitment and aggravate neurological deficits in different ICH mouse models (148).

CL is a mitochondrial membrane phospholipid that supports mitochondrial function and metabolic processes and regulates neuronal and glial cell viability (149). Microglial phagocytosis and expression of neurotrophic factors are enhanced by extracellular CL while the release of inflammatory mediators and cytotoxins by activated microglia-like cells is reduced (150). In the traumatically injured brain, mitochondria are released from the systemic circulation, and the CL is exposed on its surface. Mitochondria exposed to CL exhibit a high level of procoagulant activity and contribute to the development of coagulopathy associated with TBI (151). TBI leads to increased mitophagy in the human brain and promotes oxidation of the CL, thus triggering neuronal apoptosis. CL is regarded as a marker of eliminating injured mitochondria, which in turn reduces neuronal death and decreases behavioral impairments (152). Extracellular mitochondria (Ex-mito) were detected in the CSF of the SAH rat model as well as in the CSF of patients with SAH. The mitochondrial membrane potential decreased following SAH. Higher mitochondrial membrane potentials in the CSF were correlated with good clinical recovery 3 months after SAH onset (68). Moreover, reduced mitochondrial membrane potential in the CSF is also associated with DCI following SAH (153). Ex-mito can activate microglia and contribute to neuroinflammation (154). Considering the crucial role of the CL in mediating mitochondrial functions (149), it would be interesting to detect the CL and explore its role in mitochondrial functions in SAH.

The mitochondria have many copies of mtDNA, which carry instructions for making ribosomal and transfer RNAs, along with important proteins needed for oxidative phosphorylation (155). mtDNA resides in the mitochondrial matrix and is linked to the inner mitochondrial membrane. It acts as a DAMP via binding to toll-like receptor-9 due to unmethylated CpG motifs, leading to upregulation of inflammation (143). Additionally, mtDNA promotes innate immune response through other PRRs, including NLRP3-, NLRC4-, AIM2-, NLRP10-inflammasome complex (156–158), and cyclic GMP-AMP synthase (cGAS)-stimulator of interferon genes (STING) (159). As a unique type of PRRs, cGAS lacks DNA sequence specificity, and cannot effectively distinguish between self and foreign DNA. Therefore, mtDNA can bind cGAS upon entry into the cytoplasm, thus activating the downstream STING and mediating neuroinflammatory responses in CNS disorders (160). Chaudhry et al. analyzed the temporal profiles of three representative mitochondrial gene fragments, including Cytochrome B (CytB), D-Loop, and Cytochrome c oxidase subunit-1 (COX-1) in the serum of aSAH patients. They revealed that serum CytB, D-Loop and COX-1 were all significantly elevated following aSAH and correlated with post-SAH complications (69). Ischemic hypoxia triggers the growth of new mitochondria and elevates mtDNA levels, leading to oxidative stress and cell death (161, 162). For example, the protein levels of cGAS and p-STING were significantly elevated following MCAO in a time-dependent manner. mtDNA was released into the microglial cytoplasm in response to I/R injury and promoted “M1” polarization of microglia. mtDNA induced the activation of the cGAS-STING and IRF3/NF-κB signaling pathways. In turn, repressing STING via C-176 treatment markedly suppressed microglial mtDNA leakage (95). In the SCI model, the downregulation of mitofusin 2 (Mfn2) in microglia contributed to an imbalanced mitochondrial fusion and division. After that, mtDNA was released from microglia and activated the cGas-Sting signaling pathway (163). Gu et al. reported that oxyhemoglobin (OxyHb) increased cytosolic mtDNA levels in microglia. Microglia-derived mtDNA further activated the AIM2 inflammasome and IL-1β and IL-18 release following oxyHb stimulation. Suppressing AIM2 markedly relieved microglia-mediated neuroinflammation after ICH (164). Treatment with recombinant fibroblast growth factor 21 (rFGF21) improved neurological deficits and neural apoptosis by relieving microglia-mediated neuroinflammation. Specifically, rFGF21 restrained microglial mtDNA release into the cytoplasm, thus dampening the activation of the cGAS-STING pathway (165). In copper-induced damage to astrocytes, mitochondrial ROS (mtROS) levels are increased, resulting in mitochondrial damage and mtDNA release into the cytoplasm. mtDNA activates the cGAS-STING pathway and induces NLRP3 inflammasome-associated pyroptosis (96). Therefore, mtDNA is considered a potent activator in both microglia and astrocytes.

### 3.4 S100 family members

**Figure 5:**
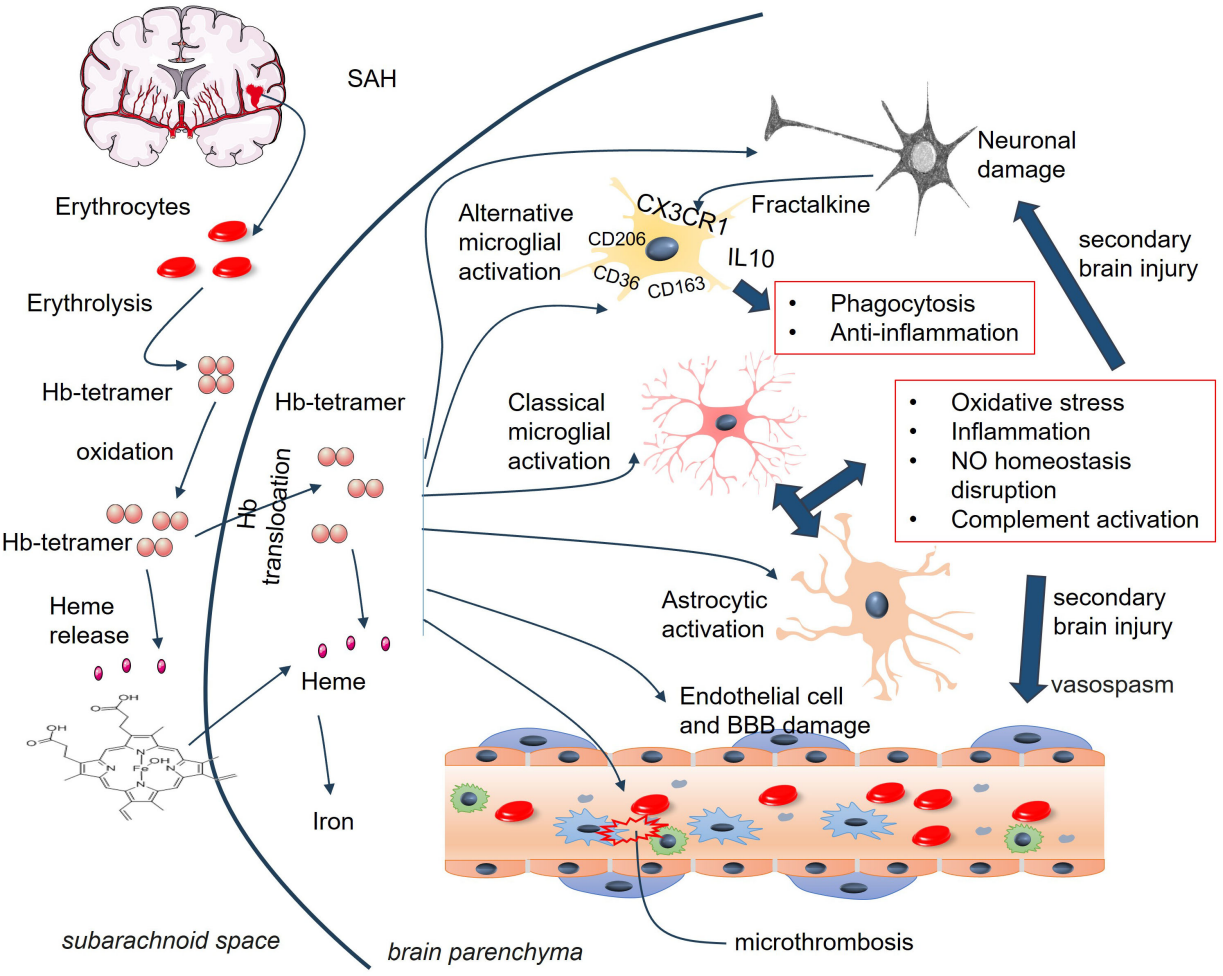
DAMPs of peroxiredoxin family members and S100 family members on astrocyte and microglia activaton. S100B binds TLR2 and TLR4 on astrocytes and promotes NF-κB activation. Elevated peroxiredoxins (1,2, 6) bind TLR2, TLR4, and RAGE on microglia and induce the proinflammatory reactions of microglia. BBB, blood-brain barrier; CCL, Chemokine C-C motif chemokine ligand; CXCL, C-X-C chemokine ligand; IL-6, interleukin-6; iNOS, inducible nitric oxide synthase; NO, nitric oxide; TLR, toll-like receptor; TNF-α, tumour necrosis factor-alpha.

There are more than 20 S100 family members. Among them, S100 calcium-binding protein B (S100B) is a Ca^2+^-binding protein that is concentrated mainly in astrocytes and is selectively expressed in neurons and peripheral cells, where it exerts differential effects (166). S100B levels in the CSF and blood are significantly elevated following TBI or stroke and are associated with the severity of brain injury. Thus, S100B has significant potential as a diagnostic biomarker in these diseases (167–169). Moreover, increasing evidence now points to S100B as a DAMP that triggers a tissue reaction to damage (170) (**Figure 5**). The biological functions of S100B in neurons in SAH are strongly linked to its concentration, leading to either neuroprotection or neurotoxicity (171). S100B is involved in cerebral vasospasm and brain damage in SAH (172–174). The S100B level is consistent with the development of reactive astrogliosis, and its role in mediating oxidative stress and neuroinflammation has been identified. For example, S100B expression and autocrine signaling in astrocytes are promoted by minimal hepatic encephalopathy in rats. Upon S100B stimulation, VEGF autocrine expression is facilitated and further contributes to the interaction between VEGFR2 and COX-2. The NF-κB pathway is activated, which eventually leads to inflammation and oxidative stress in MHE astrocytes (175). When astrocytes are stimulated by S100B, they are polarized into a proinflammatory phenotype with enhanced expressions of TLR2, inducible nitric oxide synthase (iNOS) and IL-1β. Thus, oxygen-glucose deprivation-induced neuron death is significantly aggravated. Moreover, S100B promotes the NF-κB pathway activation dose-dependently and upregulates endogenous RAGE in astrocytes (176). Moreover, high S100B stimulates microglial migration via the upregulation of chemokines (CCL3, CCL5, and CXCL12) and chemokine receptors (CCR1 and CCR5), which are dependent on RAGE expression (97). The proinflammatory reactions of microglia can also be accelerated by S100B/RAGE-dependent activation of the JNK and NF-κB pathways (98).

In addition to S100B, other S100 family members show significant alterations in ICH. For example, the level of calprotectin, a stable heteromorphic dimer complex of S100A8 and S100A9, was significantly greater in SAH patients than in healthy controls. Higher calprotectin levels predict poorer prognosis in SAH patients, and they are related to delayed cerebral ischemia and the complications of secondary pneumonia (83). Elevated levels of S100A12 are correlated with increased inflammation, greater hemorrhagic severity, and higher short-term mortality in patients with ICH (84, 177). S100A8/A9 and S100A12 represent prototypes of DAMPs in multiple diseases, such as myocardial infarction (178), COVID-19 (179), and atopic dermatitis (180). Recruited neutrophils to the infarct lesions produce specific alarmins of S100A8 and S100A9, which bind to TLR4 and prime the NLRP3 inflammasome activation in naïve neutrophils (178). S100A8 and S100A9 are also involved in intracranial hemorrhage (181–183). For example, S100A8 was significantly upregulated in microglia following SAH or OxyHb administration. Specific microglial S100A8 downregulation attenuated expressions of ferroptosis-related genes (including *ALOX15, ACSL4, PTGS2, NOX2* and *NOS2*), production of lipid peroxidation products, and ROS in microglia, and also relieved neural function deficits and neuronal apoptosis in SAH mice (181). Knocking out S100A9 exerted neuroprotective effects in SAH mice and mitigated SAH-induced neurogenic pulmonary edema (NPE). The TLR4/MYD88/NF-κB signaling pathway and the expression of inflammatory molecules (IL-1β and TNF-α) are downregulated by the knockout of S100A9 (182). Compared with those in the superficial temporal arteries, the gene levels of S100A8, S100A9, and S100A12 were upregulated in the aneurysmal domes of ruptured intracranial aneurysms. These three genes are associated with inflammatory and immune responses and phagocytosis (183). Recently, Wang et al. suggested that SAH patients had significantly higher CSF S100A9 level. S100A9 protein can activate the TLR4-NF-κB pathway and increase the activation of inflammation, and ultimately aggravate nerve injury (184).

### 3.5 Peroxiredoxin family members

Peroxiredoxins (Prxs, also known as PRDXs), a group of sulphhydryl-dependent peroxidases, have been identified as immune-modulating DAMPs in mammals (185). At least 6 PRDX family members (PRDX1-6) have been identified, all of which are altered during ischemic stroke or ICH (99, 186–190) (**Figure 5**). PRDX1 is believed to be a significant form of PRDX that is induced by hemorrhagic stress during the acute and subacute stages of ICH. Exogenous PRDX1 enhances the production of proinflammatory mediators (NO, TNF-α, and IL-6) in macrophages by activating the TLR4–NF-κB p65 axis. Within 72 hours of ICH, murine neurological deficits, cerebral edema, and various neuropathological changes, including neuron injury, activation of astrocytes and microglia/macrophages, as well as invasion by neutrophils and T lymphocytes, are induced by ICH. Moreover, ICH stimulates PRDX1 expression and extracellular release (191). PRDX2 expression is elevated in the CSF of SAH patients and is associated with brain injury and prognosis (192). Prx2 can cause brain injury following ICH by inducing brain swelling, microglial activation, neutrophil infiltration, neuronal death, and neurological deficits via the TLR4/NF-κB pathway (193). PRX-2 upregulated lipocalin-2 (LCN-2) in ICH mice. However, LCN-2 knockout partly reduced the effects of PRX-2 on neutrophil infiltration and microglia/macrophage activation and ultimately brain damage (194). PRDX3 is located mainly in the mitochondria of neurons. Neuronal overexpression of PRDX3 prevents neuronal death via the mitochondria-mediated pathway in SAH rats (188). Following ICH, Txnrd2, Trx2 and Prx3 were increased in neurons and astrocytes. Txnrd2 downregulation facilitates ROS production, peroxidation and endoplasmic reticulum stress, which are associated with reduced Trx2 and Prx3 expression (195). Similarly, overexpression of Prx4 or Prx5 reduces BBB damage and has antioxidative stress and antiapoptotic effects in ischemic stroke and SAH models (189, 190). The release of astrocyte-produced PRDX6 contributes to neuroapoptosis in ischemic stroke (99). PRDX6 is expressed mainly in astrocytes, and PRDX6-phospholipase A2 (iPLA2) is involved in the activation of astrocytes and microglia. Further investigations revealed that PRDX6-iPLA2 worsens the production of ROS and activation of microglia caused by astrocytes through the activation of mitochondrial fission pathways dependent on Nox2 and Drp1 (100).

### 3.6 Other DAMPs

In addition to the abovementioned DAMPs, there are other DAMPs whose expression is elevated during stroke. These DAMPs, such as heat shock protein (HSP)70 (196), cold-inducible RNA-binding protein (CIRP) (197), and IL-33 (198), are significantly associated with the outcomes of stroke patients. These DAMPs have diverse functions, including mediating neuroinflammation, neuronal and endothelial cell damage, BBB integrity, and microglial and astrocytic activation (199–203). For example, CIRP levels are increased in the blood of ICH patients and are associated with poorer clinical outcomes. Knocking out CIRP significantly attenuated brain edema; neurological deficits; and the expression of proinflammatory cytokines (including IL-6, TNF-α, and IL-1β) and TLR4-NF-κB signaling (199). Neural-specific CIRP KO prevented neuronal apoptosis and alleviated glial cell activation in TBI mice. Neruron-derived secretion of extracellular CIRP (eCIRP) contributes to neural damage and glial-mediated inflammation (204). Histone H3 is one of the core histones responsible for binding DNA and regulating gene expression within the nucleus. Histone H3 can be released into the extracellular space by activated or damaged cells, where it then acts as a DAMP and triggers immune responses by interacting directly with TLRs and RAGE (205).

## 4 Crosstalk between microglia and astrocytes in SAH

**Figure 6:**
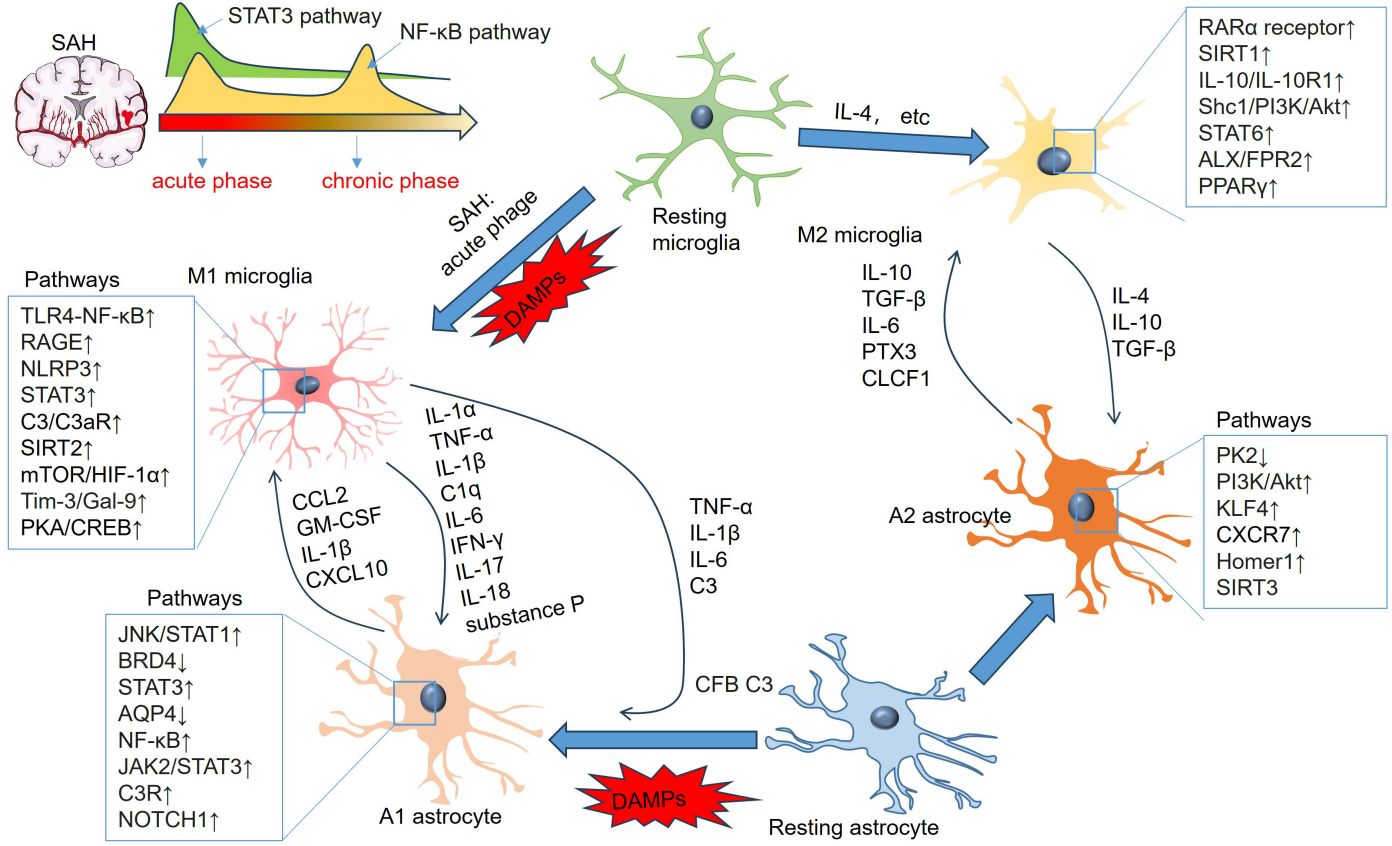
Signaling pathways involving microglial and astrocytic activation and inflammatory mediators in astrocyte-microglia crosstalk. “M1” microglia-produced TNF-α, IL-1β, IL-6, IFN-γ and IL-17 induce “A1” polarization of astrocytes. “A1” astrocytes-produced CCL2, IL-1β, and CXCL10 also mediate the proinflammatory reactions of microglia. “M2” microglia produce IL-4, IL-10, TGF-β and substance P, which promote “A2” polarization of astrocytes. “A2” astrocytes produce elevated IL-10, TGF-β, IL-6, PTX3, and CLCF1, which can mediate “M2” polarization of microglia.

Microglia are among the first nonneuronal cells on the scene of the innate immune response to ICH (206). Neves and colleagues also reported that the number of CD11+ microglia in the ipsilesional striatum and sensorimotor cortex was increased 6 h after ICH. The number of glial fibrillary acidic protein (GFAP)+ cells in the ipsilesional striatum increased 24 h after ICH. However, in the ipsilateral sensorimotor cortex, the number of GFAP+ cells was reduced 6 h after ICH, then gradually increased and peaked at 7 days post-ICH (207). Astrocyte–microglia communication can significantly affect their activation state, leading to further enhanced activation or attenuated activation. DAMPs and many other bioactive molecules are involved in their interaction, including cytokines, chemokines, chemokines, innate immune mediators (such as C3), growth factors, mitogenic factors, NO, reactive oxygen species, MMPs, neurotransmitters, gliotransmitters, tissue damage molecules (such as ATP), and metabolic mediators (such as glutamate and lactate) (208) (**Table 3**, **Figure 6**). Although most of these bioactive molecules can be expressed by both microglia and astrocytes, their expression levels, as well as their temporal and spatial characteristics, are significantly different after ICH (209–212).

### 4.1 Inflammatory cytokines

Following SAH, many inflammatory cytokines are elevated in the serum and CSF, including proinflammatory cytokines (e.g., IL-1α, IL-1β, IL-2, IL-6, IL-8, IFN-γ, TNF-α, IL-15, IL-17, and IL-18) and anti-inflammatory cytokines (e.g., IL-Ra, IL-4, IL-13, IL-10, and TGF-β) (**Table 1**). IL-1α and TNF-α are two representative neurotoxic cytokines produced by microglia in SAH (213, 214). Activated microglia exhibit increased release of IL-1α, TNF and C1q, which together promote A1 polarization of astrocytes and induce the death of neurons and oligodendrocytes (215). Administration of an IL-1 receptor antagonist (IL-1Ra) mitigated BBB breakdown in an SAH rat model. In addition, IL-1Ra completely reversed heme-induced cellular injury in organotypic slice culture (OSC) by blocking IL-1 signaling (214). Pure microglial cultures, but not pure astrocyte cultures, released IL-1ra in response to treatment with CM from injured primary neurons. Endogenous IL-1ra produced by microglia is neuroprotective in cerebral ischemia or excitotoxicity (216).

IL-1β and IL-18 are also members of the IL-1 family. These two proinflammatory factors can be released from activated microglia and astrocytes, where they mediate neurotoxic effects and BBB damage during ICH (217–219). The cultured human fetal astrocytes exhibit significant reactions to IL-1β produced by human fetal microglia, but not the primary stimulus LPS (220). HMGB1 indirectly upregulates AQP4 expression in astrocytes through diffusible factor(s), such as IL-1β from microglia (87). These two studies indicate that astrocyte activation may be a secondary consequence of microglial activation. In an SAH rat model, the neutralization of IL-1β activity markedly attenuated the increase in the S-100B level (an astrocyte) and the number of leukocytes migrating into the CNS (221). IL-33 is another member of the IL-1 family (222). In the microglia–astrocyte circuit, IL-33 is produced by astrocytes, which activate ST2–AKT signaling in microglia, thus supporting microglial metabolic adaptation and phagocytic function during early development (223). Following SAH, IL-33 was primarily expressed by neurons and astrocytes rather than by microglia. Additionally, there is a notable correlation between the expression of IL-33 and IL-1β (224). In an ICH rat model, IL-33 attenuated short-term and long-term neurological deficits by reducing neuronal degeneration and transforming the “M1” to “M2” polarization of microglia (225). The results suggest that IL-33 could have a significant impact on the inflammatory reaction after SAH.

LPS (100 ng/ml) was treated with primary astrocytes and microglia for different durations (8 h or 24 h). Short-term LPS exposure induced bidirectional polarization of both microglia (M1 and M2) and astrocytes (A1 and A2). A longer duration of LPS exposure enhances proinflammatory and neurotoxic microglial and astrocytic polarization (M1/A1). However, the administration of IL-1 antagonists had no significant effect on the modulation of specific microglia or astrocyte activation pathways, suggesting that there are other mediators involved in microglia–astrocyte interactions (226). Recently, Shi et al. reported that IL-15 was significantly elevated in astrocytes from patients with ICH and wild-type mice subjected to experimental ICH. Brain water content and neurological disorders were facilitated by astrocytes with enhanced IL-15 expression, accompanied by increased microglial accumulation near astrocytes in perihematomal tissues. Moreover, there was a significant increase in microglial expression of CD86, IL-1β, and TNF-α in GFAP-IL-15^tg^ mice after ICH. Additionally, the worsening of ICH injury in GFAP-IL-15^tg^ mice was reduced by depleting microglia. The study showed that IL-15 plays a role in the communication between astrocytes and microglia, leading to worsened brain injury after ICH (227).

As a proinflammatory cytokine, IL-6 not only plays a major role in the pathobiology and pathophysiology of aneurysm formation and aneurysmal SAH (aSAH) but also has a close correlation with DCI/vasospasm and secondary brain injury (228). In the brain, IL-6 release is closely tied to reactive astrocytes, which are critical for blood product breakdown following SAH (229). Recently, Lucke-Wold et al. reported that IL-6 expression was elevated and peaked 3 days after SAH. Knocking out IL-6 in mice significantly mitigated vasospasm, secondary cascades, and the reduction in cerebral blood flow after SAH. The infiltration of macrophages occurred in regions of vasospasm adjacent to regions of microglial activation and increased the expression of IL-6 receptors. IL-6 blockade prevented vasospasm, improved neurologic performance, inhibited “M1” polarization of microglia, and enhanced “M2” polarization. In addition, they indicated that the release of IL-6 at the endothelial border next to reactive astrocytes led to an elevation in Caveolin 3 levels. Caveolin 3 facilitates the BBB’s preparation for peripheral macrophage diapedesis (230).

Several anti-inflammatory cytokines, such as IL-4, IL-10 and TGF-β, play essential roles in microglia–astrocyte interactions (231, 232). Following SAH, IL-4 can mediate hematoma resolution through activating STAT6/ST2 signaling in microglia/macrophages (233), and IL-10 has similar functions by regulating CD36 (124). IL-10-redirected astrocytes can relieve the activation of microglia by suppressing IL-1β expression and upregulating CX3CR1 and interleukin 4 receptor-α (IL-4Rα) in microglia. IL-10-mediated effects depend on astrocyte-derived TGF-β (234). Interestingly, Taylor et al. reported that TGF-β1 administration dampened the inflammatory reactions of microglia and improved functional outcomes in an ICH murine model (235). These authors suggested that astrocytes might provide an early source of TGF-β1 that initiates phenotypic modulation in microglia, considering that astrocytic TGF-β1 can restrain neuroinflammation and motor function deficits in an ischemic stroke mouse model (236).

There are more cytokines that mediate the interaction between microglia and astrocytes in other CNS diseases. For example, astrocyte-derived IL-3 induces microglia to undergo transcriptional, morphological, and functional changes that equip them with a rapid immune response, improved movement abilities, and the capability to gather and remove Aβ and tau accumulations. Thus, AD pathology and cognitive decline can be significantly mitigated (237). Following acute SCI, TLR4/p38 MAPK signaling promotes the production of elevated IL-18 by microglia, which stimulates IL-18R on astrocytes. IL-18R increases phosphorylated NF-κB level in astrocytes and causes GFAP upregulation (238). Radiation significantly promoted the expression of TNF-α, IL-1β and ICAM-1 in primary microglia. Lonizing radiation failed to induce ICAM-1 expression in astrocytes without microglia-produced TNF-α and IL-1β, suggesting that microglia-derived proinflammatory cytokines may be necessary for ICAM-1 expression in astrocytes during CNS radiation injury (239).

### 4.2 Chemokines

In patients with SAH, many chemokines, including CCL2, CCL4, CCL5, CCL7, CCL11, CCL13, CCL19, CCL20, CXCL1, CXCL5, CXCL6, CXCL8, CXCL12, and CXCL16, are upregulated in the CSF or serum. These chemokines are associated with poorer outcomes in SAH patients, indicating their potential roles in SAH pathology (31–34). Astrocytes play a major role in producing various chemokines, including CCL2, CXCL1, CXCL10, and CXCL12, while microglia express certain chemokine receptors like CCR2 and CXCR4 (216), which implies a strong association between microglia and astrocytes.

The CCL2–CCR2 chemotactic system is one of the major signaling pathways that induces inflammation and apoptosis in the brain. Jin et al. conducted an *in vitro* study and reported that TNF-α stimulated CCL2 release from astrocytes. The conditioned medium of TNF-α-stimulated astrocytes increased the “M1” polarization and migration ability of microglia. The above effects were significantly reversed by knocking down CCL2 in astrocytes or inhibiting CCR2 in microglia (240). They later confirmed the involvement of the CCL2–CCR2 pathway in astrocyte–microglia interactions in rats with surgery-induced cognitive dysfunction. Targeting CCL2-CCR2 signaling significantly inhibited astrocyte-mediated microglial activation (241). In a collagenase-induced ICH mouse model, knocking out CCL2 or CCR2 can significantly decrease hematoma volume, BBB damage, microglial activation/migration, and leukocyte and neutrophil infiltration (242). Sphingosine-1-phosphate receptor 3 (S1PR3) and its ligand, sphingosine 1-phosphate (S1P), are dramatically increased following ICH. S1P upregulated the expression of CCL2 in astrocytes. CAY10444 (an S1PR3 antagonist) significantly attenuated CCL2 in astrocytes, improved neurological functions and BBB integrity, and suppressed microglial proliferation and M1 polarization in SAH rats (243).

CXCL10 is a vital chemokine produced by activated A1 astrocytes. A1 astrocyte-secreted CXCL10 enhances STAT3 phosphorylation in neurons via CXCR3, leading to ferroptosis-associated lipid peroxidation in epileptic brains (244). In the cuprizone model, CXCL10, CCL2, and CCL3 are three chemokines with distinct alterations. CXCL10, which is produced mainly by astrocytes, participates in the initiation of microglial activation. Early microglial activation was significantly reduced only in CXCL10-deficient mice but not in CCL2- and CCL3-deficient mice (245). Inhibiting astrocytic P2Y(1)R suppressed CXCL10 expression in astrocytes, thus decreasing infarct volume and improving motor functions in rats with cerebral ischemia (246). In the ICH mouse model, treatment with the colony-stimulating factor-1 receptor antagonist GW2580 repressed the proliferation and inflammatory response of microglia, after which astrocytes became activated and produced elevated CXCL10 around brain lesions. The CXCL10/CXCR3 axis is essential for the brain homing of regulatory CD8+CD122+ T cells, which exert synergistic anti-inflammatory effects with microglia (247).

CCL5 plays a critical role in initiating the intrinsic neuronal regeneration system following brain injury (248). CCL5 was among the genes whose expression was upregulated the most in astrocytes activated by IL-1α, TNFα, and C1q treatment. Following hemorrhagic stroke, CCL5 expression is elevated in the mouse brain. Knocking out CCL5 in astrocytes improved neurobehavioral outcomes and improved BBB integrity (249). CCL5 can enhance the proinflammatory reactions of microglia, as evidenced by both *in vitro* and *in vivo* experiments (250, 251). CCL5 and CCR5 (C-C chemokine receptor 5) expression increased after ICH and peaked at 24 hours. CCR5 was positively expressed in neurons, microglia, and astrocytes. CCL5/CCR5 axis activation aggravated neurological deficits in ICH mice by mediating neuronal pyroptosis, BBB disruption, and the activation of microglia, astrocytes and monocytes, partially through the CCR5/PKA/CREB/NLRP1 signaling pathway (252, 253).

### 4.3 Innate immune mediators

As vital mediators in the innate immune system, complement components have essential roles in several neurological disorders. The activation of this pathway occurs when the recognition protein C1q from the complement binds to the cell surface, resulting in the activation of C3 convertase and the cleavage of C3 into C3a and C3b fragments (254). In the CNS, C1q is principally produced by microglia, and C3 is produced by activated astrocytes (219, 255). Microglia-produced C1q can induce the A1 neurotoxic phenotype of astrocytes, which then produces elevated C3 and contributes to increased microglial activation via the C3–C3aR pathway (254). The microglia-mediated C3-C3aR pathway plays a crucial role in neuroretinal development by regulating developmental astrocyte and vascular network spatial patterning (256). Several studies have shown that intervening in C1q/C3-C3aR pathway-mediated astrocyte–microglia crosstalk has high potential for treating Parkinson’s disease (257), posthemorrhagic hydrocephalus (PHH) (258), neuropathic pain (259, 260), and Alzheimer’s disease (261).

Notably, excessive complement activation, including C1q/C3-C3aR signaling, also plays a prominent role in ICH (262). Higher complement C1q levels are associated with poorer outcomes in patients with acute ICH (263). Elevated C3a in the CSF correlates with delayed ischemic neurological deficits in SAH patients via activation of the coagulation system (264). C3 deficiency or C3aR inhibition effectively alleviated neurological deficits, brain edema, erythrolysis, inflammatory cell infiltration, and neuroinflammation following SAH (265–267). For example, inhibition of C3aR decreased abnormal microglial activation by reducing p53-induced death domain protein 1 (Pidd1) and protein kinase RNA-like ER kinase (PERK). In the subacute phase of SAH, intranasal administration of C3a, a proteolytically activated peptide of the complement component C3, significantly enhances “M2” polarization of microglia, suppresses astrocyte reactivity and improves cognitive function (268). Therefore, C3-C3aR signaling might also be involved in microglia–astrocyte crosstalk in SAH.

### 4.4 Growth factors

Several growth factors, such as insulin-like growth factor 1 (IGF1) (269) and VEGF (270), are involved in the pathologies of SAH, and they are potential mediators of microglia–astrocyte crosstalk. In a tri-culture composed of neurons, astrocytes, and microglia, microglia presented elevated IGF-1 expression and reduced caspase 3/7 activity. In response to LPS stimulation, astrocyte hypertrophy and neuron apoptosis are promoted by microglia, which increase the secretion of proinflammatory cytokines (e.g., TNF, IL-1α, IL-1β, and IL-6). During glutamate-induced excitotoxicity, microglia play a significant neuroprotective role in tri-culture by relieving reduced neuron loss and astrocyte hypertrophy (271). During the early stage of ICH, sustained microglial depletion induces disorganized astrocytic scarring, aggravates neutrophil infiltration, and impairs tissue repair. Spatial transcriptomics (ST) analysis revealed that IGF1 produced by microglia mediates protective astrocytic scar formation, which is further facilitated by repopulating microglia (RMs). During the chronic stage of ICH, astrocytic scars exhibit destructive functions instead of primary protective effects. Delayed microglial depletion could partly reverse this phenomenon (210). Microglia play different roles in modulating proinflammatory and neurotoxic activities in astrocytes by producing TGFα and VEGF-B in an experimental autoimmune encephalomyelitis (EAE) mouse model. TGFα produced by microglia limits the pathogenic activities and development of EAE by acting through the ErbB1 receptor in astrocytes. On the other hand, VEGF-B released by microglia worsens EAE by activating FLT-1 signaling in astrocytes (272). In ischemic stroke, astrocyte-derived VEGFD, which acts on VEGFR3 in astrocytes and microglia, contributes to crosstalk dysfunction and proinflammatory activation of these two types of glial cells, thereby mediating neuronal damage (273). Hypertonic saline (HS) alleviates ischemic blood–brain barrier permeability in a cerebral ischemia rat model by suppressing the NLRP3/IL-1β signaling axis in microglia. In addition, HS inhibited VEGF expression in astrocytes by restraining the activation of the IL-1β/IL-1R1/NF-κB signaling pathway (274).

### 4.5 Extracellular vesicles

EVs have gained tremendous attention for the diagnosis and treatment of CNS disorders. EVs play important roles in intercellular communication, reparative processes, and as potential drug delivery vehicles. Due to their lipid membranes, EVs can easily cross the BBB and establish communication with target neurons and glia deep within the brain (275). Omics analysis revealed that many microRNAs (miRNAs) and proteins can be delivered by EVs, and their alterations are regarded as hallmarks of SAH (276–278). The administration of blood-derived EVs improved recovery after ICH (279). EV-enriched serum amyloid A1 (SAA1) is significantly increased in EVs from the plasma of ICH patients. SAA1 stimulation exacerbates neuroinflammation by increasing the accumulation of microglia and astrocytes (280). Previous studies have shown that EVs play a vital role in astrocyte–microglia communication (281, 282). For example, reactive astrocytes produce dual immunoglobulin domain-containing cell adhesion molecules (DICAMs), which are mainly secreted via extracellular vesicles (EVs). EV-delivered DICAM plays a significant role in modulating neuroinflammation by transforming microglia into the M2 phenotype (283). A2-reactive astrocyte-derived exosomes, which highly express miR-628, significantly attenuate pyroptosis and BBB damage following ischemic stroke and promote M2 microglial polarization (284). Following TBI, astrocyte-produced exosomes are enriched with miR-873a-5p, which mediates M2 microglial polarization by regulating extracellular regulated kinase (ERK) and NF-κB pathways (281). Homer scaffolding protein 1 (Homer1) has been found to play a protective role in ICH via the phenotypic conversion (A1 to A2) of astrocytes (285) as well as microglial activation (286). A2 astrocytes can produce EVs that contain high levels of Homer1a. EV-encapsulated Homer1a enhanced the conversion of A1 to A2 astrocytes in ICH mice, improved neurological functions and reduced neuronal injuries through repressing the RAGE/NF-κB/IL-17 signaling pathway (287).

### 4.6 MMPs

Following SAH, MMPs exhibit altered expression and play a paramount role in pathological processes, such as BBB disruption, neuroinflammation and neuronal damage (39, 288–290). Knocking out TLR2 markedly attenuated MMP9 expression in astrocytes, thus mitigating BBB damage, neutrophil infiltration, and proinflammatory gene expression in brain lesions (291). Recently, Feng et al. reported that increased MMP-9 was derived mainly from reactive astrocytes after SAH. Downregulating NDRG2-PPM1A signaling inhibited MMP-9 expression in astrocytes after SAH and attenuated BBB damage (52). In the ICH mouse model, the expression of extracellular matrix metalloproteinase inducer (EMMPRIN) and MMP9 was elevated in both GFAP-labeled astrocytes and Iba1-labeled microglia. EMMPRIN inhibition by an anti-EMMPRIN (CD147) monoclonal antibody exhibited neuroprotective effects by suppressing MMP9 (292). The dipeptidyl peptidase (DPP4) inhibitor omarigliptin markedly decreased hematoma formation, neurobehavioral deficits, microglial/macrophage activation and neutrophil infiltration in ICH mice by repressing DPP4 expression. Omarigliptin also decreased the expression of DPP4 and MMP9 and inhibited CX43 expression in astrocytes (293). Therefore, MMP9 might be a potential mediator involved in astrocyte–microglia crosstalk in SAH.

### 4.8 Other bioactive molecules

OPN is a multifunctional extracellular matrix glycoprotein that is significantly induced in reactive astrocytes and capillary endothelial cells and peaks at 72 hours after SAH. OPN is necessary for repairing disrupted BBB after SAH (294). The administration of recombinant OPN has shown therapeutic effects by modulating diverse pathological processes in SAH, such as brain edema (295), neuroinflammation (296) and neutrophil infiltration (297). Notably, OPN altered microglial activation states from “M1” to “M2” polarization (296). Hematogenous macrophages infiltrating the inner border zone of infarcts can produce OPN and promote reestablishment of the BBB after ischemic stroke by mediating reactive astrocyte polarization (298).

Lipocalin-2 (LCN2) is a neutrophil gelatinase associated-lipocalin of the lipocalin superfamily. LCN2 can be produced by neurons as a “help-me” signal in the stroke-damaged brain. LCN2 can mediate microglia and astrocyte activation (299). In addition, astrocytes, as well as invading immune cells, are important sources of LCN2 in the peri-infarct region of the rat brain after ischemic stroke (300). LCN2-treated microglia presented increased IL-10 release and increased phagocytosis. In astrocytes, LCN2 upregulated BDNF and thrombospondin-1. The conditioned media from LCN2-treated microglia and astrocytes protected neurons against oxygen–glucose deprivation (299). Following SAH, microglia, astrocytes, and neurons all presented elevated LCN2 expression, which plays hazardous roles by mediating microglial ferroptosis-induced neural injury (301). The LCN2 receptor 24p3R is expressed in oligodendrocytes, astrocytes, endothelial cells, and pericytes in the white matter. LCN2 knockout improves BBB disruption caused by SAH (302). In addition, inhibiting the expression of microglial LCN2 early could help reduce ferroptosis-induced harm to oligodendrocytes and related neurological impairments, presenting a hopeful neuroprotective approach after ICH (303). Together, LCN2 might be a vital mediator of glial cell crosstalk in SAH.

### 4.9 Metabolic interactions

The brain is one of the most energy-consuming organs in the body. Astrocytes are considered an essential source of blood-borne glucose or its metabolites to neurons. Astrocytes and microglia can produce a large amount of mitochondrial ROS and transport glycolytically derived metabolites, like L-lactate, L-serine, and glutamate to neurons. These metabolites support energy requirements, maintain the balance of oxidation-reduction reactions, and influence neurotransmitter-receptor activity (304, 305). Following SAH, alterations in cerebral metabolism occur, along with the buildup of metabolites including glucose, lactate, pyruvate, and glutamate. These phenomena might disrupt oxidative metabolism within the brain. Such disruption can trigger secondary brain damage and is associated with an unfavorable prognosis (306–308). For instance, a decline in glucose levels and a sharp increase in glutamate were observed prior to the onset of DCI after SAH, accompanied by elevations in the lactate/pyruvate and lactate/glucose ratios. When cerebral microdialysis (CMD) monitoring was carried out outside the lesion area, the surge in glutamate was not evident, yet significant changes were still noted in glucose levels and the lactate/glucose (L/G) ratio. Therefore, glucose and the L/G ratio have the potential to prompt additional diagnostic evaluations or therapeutic interventions at an earlier stage (309).

Glucose transporter-1 (GLUT1) is the main astrocyte glucose transporter. Astrocytic GLUT1 has a pivotal role in mediating astrocyte insulin signaling and brain purinergic signaling, which are essential for sustaining metabolic and antioxidant support for neurons, and improving brain function and systemic glucose metabolism (310). Astrocyte-specific deletion of GLUT1 does not affect sensorimotor and memory functions in male mice suffering stroke. Astrocytes with GLUT1 deletion maintain normal resting glucose levels but exhibit a more than two-fold increase in glucose consumption, indicating enhanced metabolic activity (311). GLUT1 exhibits higher expression in microglia with LPS + IFNγ treatment. It acts as a main mediator in controlling glucose uptake and facilitating glycolysis in microglia, particularly under inflammatory conditions (312). After ICH, reduced glucose uptake of microglia was observed in the perihaematomal region. The downregulation of GLUT1 and HK2 in microglia resulted in a reduced amount of glucose-6-phosphate (G-6-P), impaired early-stage glycolysis, and proinflammatory reactions of microglia after ICH (313). Elevated lactate can be produced by activated microglia. Its concentration is significantly increased in the core and penumbra regions of hemorrhagic foci (314). Lactate is not only beneficial for the proliferation, cell survival, migration, and phagocytosis property of the microglia (314), but also protects neurons from oxygen and glucose deprivation/reoxygenation (OGD/R) injury (315). Normally, lactate is transferred from astrocytes to neurons to match the neuronal energetic needs, and to provide signals that modulate neuronal functions, including excitability, plasticity and memory consolidation. However, increased brain lactate derived from astrocytes aggravates ischemic brain injury (316). Elevated lactate levels in stroke patients are inversely correlated with astrocytic mitochondria, which might affect astrocyte-to-neuron mitochondria transfer (317). Those studies suggest that lactate and other metabolites have potential roles in microglia-astrocyte crosstalk.

Pyruvate Kinase M2 (PKM2), a key glycolytic enzyme, has an essential impact on glial metabolic reprogramming and subsequent neuroinflammation (318, 319). PKM2 localizes in the cell body of neurons and mediates high levels of aerobic glycolysis, thus protecting against oxidative stress (320). In astrocytes, PKM2 is crucial for the astrocyte-neuron lactate shuttle, which helps maintain energy metabolism in neurons (319). PKM2 is upregulated in microglia in multiple CNS disorders, including ischemic stroke (321), TBI (322), and ICH (323), and it drives metabolic reprogramming, inducing microglial pro-inflammatory polarization and chemotaxis. TEPP-46 is a PKM2 activator by mediating the formation of the PKM2 tetramer and stabilizing PKM2 subunit interactions. In the TBI model, treatment with TEPP-46 improves the interaction between PKM2 and MFN2, enhances mitochondria, and reduces neuroinflammation (322). Following ICH, administering TEPP-46 significantly decreased PKM2 nuclear translocation, repressed the activation of microglia and astrocytes, and improved neurological functions of ICH mice (323). Hypoxia-inducible factor-1α (Hif-1α) mediated the nuclear translocation of PKM2 in ischemia-induced microglia. Therefore, PKM2 affected microglial polarization by associating with acetyl-H3K9 and upregulating the expression of microglial CCL2 in peri-infarct (324). Moreover, PKM2 knockdown inhibited NF-κB pathway-mediated microglial activation and production of inflammatory mediators (including C1q, TNF-α, and IL-1α), followed by reduced astrocyte C3 expression due to the C3-C3aR interaction (325). Collectively, those studies suggest PKM2 as a potential therapeutic target in stroke.

## 5 Strategies for SAH treatment by mediating astrocyte and microglial activation

**Figure 7:**
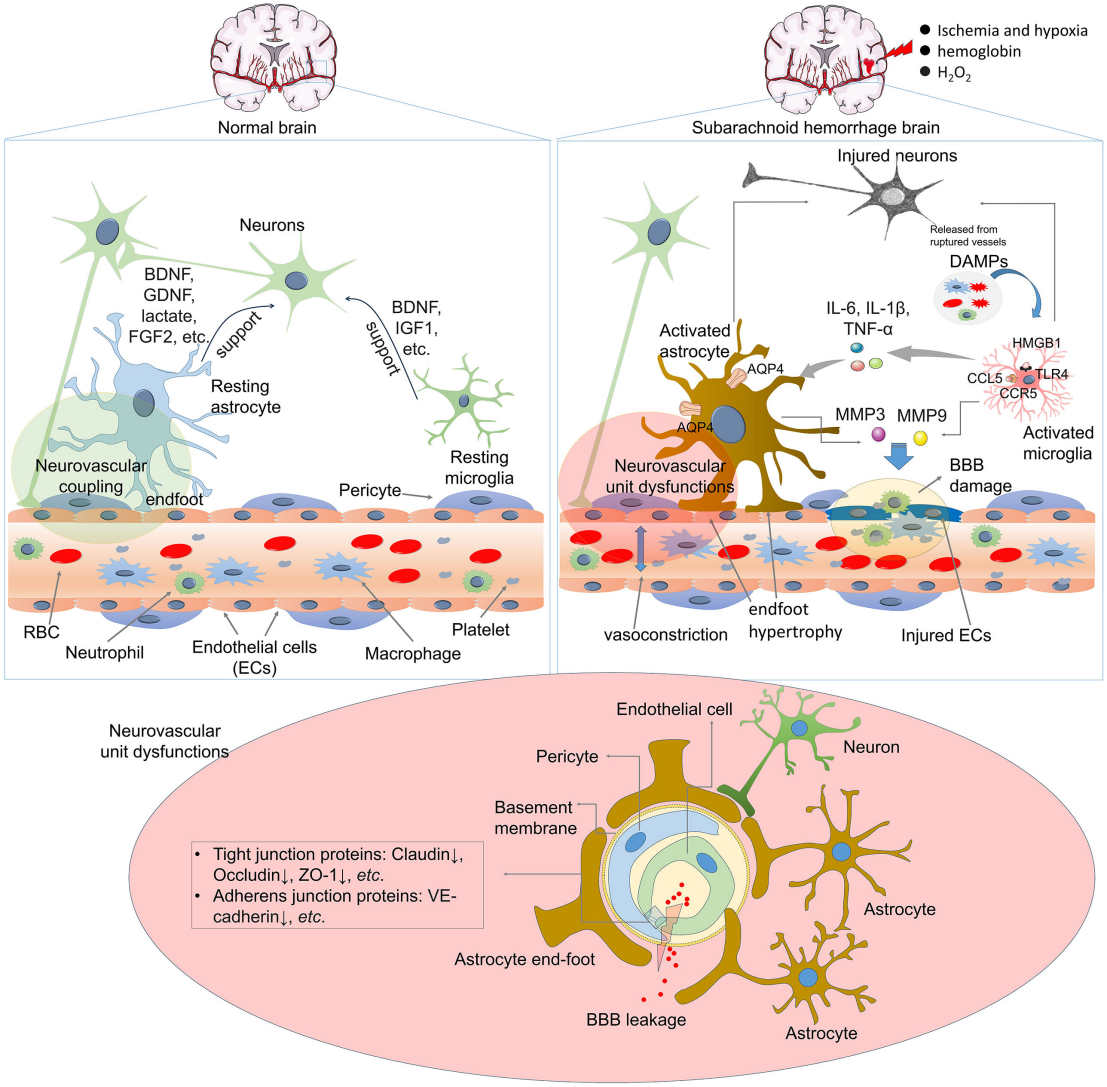
Therapeutic strategies against SAH by regulating astrocytes-microglia crosstalk. ①Targeting immune mediators, ②Targeting receptors, ③Targeting DAMPs, ④Targeting downstream pathways. Clinical pharmacological interventions against SAH by regulating microglial and astrocytic activation.

After SAH, microglia and astrocytes exhibit a complex and specific response based on the stimulus, involving unique subpopulations of each cell type. Many strategies, including the use of agonists or antagonists that target immune mediators, related receptors, related signaling pathways, inhibitors of DAMPs, anti-inflammatory drugs, stem cell therapy, and mitochondrial transfer, have been developed to treat SAH through the intervention of astrocytes and microglia (**Figure 7**).

### 5.1 Targeting immune mediators and related receptors

Neuroprotective effects can be achieved by administering antagonists of neurotoxic immune mediators or related receptors. For example, microglia-derived IL-1Ra reduced the expression of astrocytic CXCL1 caused by lack of oxygen and glucose, and also hindered the movement of neutrophils. In an ischemic stroke model, neutralizing antibody therapy against CXCL1 or the administration of recombinant IL-1Ra protein exhibited significant neuroprotective effects (326). Exogenously administered IL-1Ra can suppress IL-1β-mediated microglial astrocytic activation (327). Neurokinin 1 receptor (NK1R) is involved in the inflammatory reactions of microglia and astrocytes by mediating the release of IL-1β, IL-6, and TNF-α (328, 329). Aprepitant is a selective NK1R antagonist with the ability to cross the BBB. Aprepitant treatment significantly promoted hematoma clearance in a mouse model of ICH by suppressing M1 polarization while increasing M2 polarization of microglia (330). Aprepitant also improved the neurological functions of mice with ICH by decreasing neuronal pyroptosis, suppressing the expression of the NLRC4 inflammasome, and inhibiting the release of IL-1β and IL-18 (331). Targeting the S1P-S1PR3 axis could significantly mitigate brain injury by mediating the proinflammatory M1 polarization of microglia (332) and reactive astrocytes (333). Fingolimod (FTY720), an S1P antagonist, significantly decreases edema, apoptosis and brain atrophy in a mouse model of ICH (334) and restrains the expression of ICAM-1, INF-γ, and IL-17 in the brains of mice with ICH (335). CAY10444, an S1PR3 antagonist, significantly suppressed S1PR3, CCL2, TNF-α, and cleaved caspase-3 and relieved neuronal apoptosis following ICH (336). CAY10444 can also inhibit the expression of CCL2, p-p38 MAPK, and ICAM-1 in astrocytes and restrain the M1 polarization of microglia in an ICH model (243).

### 5.2 Targeting DAMPs and related receptors

DAMPs increase inflammation after binding to their cognate receptors on immune cells and underlie early and delayed brain injury after SAH. Thus, strategies have been developed to relieve the detrimental functions of DAMPs (337). Targeting HMGB1 with either an anti-HMGB1 antibody (338) or its inhibitors (e.g., glycyrrhizin) (339) can alleviate hemorrhage-induced brain injury by decreasing brain edema, protecting BBB integrity, reducing microglial activation and neuronal death, and suppressing the expression of inflammatory factors. Targeting RAGE and TLR4, the two putative receptors of HMGB1, also has beneficial effects on SAH. For example, FPS-ZM1 is a selective RAGE inhibitor that specifically binds to the V domain of RAGE. FPS-ZM1 treatment significantly improved neurobehavioral functions and BBB permeability, inhibited the infiltration of inflammatory cells, and downregulated the expression of IL-1β, IL-6, IL-8R, COX-2, iNOS, and MMP-9 in the perihematoma after ICH (340–342). The TLR4 antagonist TAK-242 dampens neurological deficits and brain edema and inhibits the production of inflammatory factors as well as peripheral inflammatory cell infiltration in an ICH mouse model (343).

In addition to the proinflammatory functions in hemorrhagic stroke models, HMGB1 can also help cerebrovascular repair by regulating endothelial cell functions (337). For example, Hmgb1 is highly expressed in astrocytes at birth and then decreases rapidly during the first two postnatal weeks. Astrocyte-selective ablation of Hmgb1 at birth alters astrocyte morphology and endfoot placement and then disrupts the endothelial ultrastructure. In the adult mouse, a lack of astrocytic Hmgb1 impairs neurovascular coupling and behavior (344). Hayakawa et al. reported that astrocyte-derived HMGB1 helps in neurovascular remodeling, which is mediated by endothelial progenitor cells (EPCs) during stroke recovery (345). Astrocytes increase EPC accumulation in damaged white matter by increasing the migration and tube formation of EPCs. The HMGB1-RAGE axis plays a prominent role in astrocyte-EPC signaling (346). Most recently, Qi et al. reported that low-intensity focused ultrasound stimulation (LIFUS) promoted angiogenesis and synaptogenesis in transient MCAO mice, which was associated with increased HMGB1 expression in the ipsilateral hemisphere of the brain at 14 days after focal cerebral ischemia. Astrocytes were the main cells expressing HMGB1. Inhibition of HMGB1 expression aggravated microcirculation disturbance in the ischemic brain (347). In an SAH rat model, the administration of two HMGB1 inhibitors (including ethyl pyruvate and glycyrrhizin) and the Rage antagonist FPS-ZM1 effectively reduced HMGB1 and Rage expression. However, neurovascular recovery was prevented following those treatments. Oxidized HMGB1 failed to trigger the production of TNF. However, it could enhance brain recovery through the promotion of neurotrophin expression. Instead, recombinant HMGB1 can promote brain injury by stimulating proinflammatory cytokine expression. The authors suggested that HMGB1 in the oxidized state could enhance neurovascular recovery in the late stage of SAH (348). Therefore, HMGB1 functions as a powerful regulator in the processes of brain tissue reconstruction, neurovascular restoration, and inflammatory responses subsequent to SAH. Understanding the dynamics of redox states of HMGB1 holds great promise for its application as a biomarker and in the development of therapeutic strategies for SAH.

Extracellular Hb and heme are scavenged by the acute phase plasma proteins haptoglobin (Hp) and hemopexin (Hx), respectively, thus dampening their deleterious effects (349). As the body’s first line of defense against the toxicity of extracellular Hb in SAH, the Hp level is low in the CSF, emphasizing the potential for therapeutic Hp supplementation (350). Functionally, the Hp concentrate was effective in preventing both EBI and cerebral vasospasm by obstructing the penetration of Hb into brain tissues and enhancing the drainage of free Hb through the lymphatic system in a mouse model of SAH (351).

### 5.3 Targeting downstream signaling pathways

Many signaling pathways are involved in microglial and astrocyte activation following SAH (**Figure 6**). Transcription factors, including NF-κB (352, 353), STAT3 (354, 355), and HIF-1α (356, 357), are involved in the transcription of proinflammatory genes such as TNF-α, IL-1β, and IL-6. In the SAH models, NF-κB activity showed a double elevation and peaks in rabbit brains and cultured neurons. The first NF-κB activity peak (at day 3) is involved in neuronal injury; however, the late peak (at day 10) might have no significant association with damaged neurons (358). The mRNA and protein level of STAT3 was enhanced following SAH, peaking at 24 h post-SAH (359). Over the first 24 hours post-SAH, the phosphorylations of ERK1/2 and STAT3 were enhanced at 1 h and remained elevated at 6 h and 24 h post-SAH. Though phosphorylated calcium calmodulin-dependent kinase II (CaMKII), focal adhesion kinase (FAK), and c-Jun were markedly increased at 1 h post-SAH, their levels were no longer significantly regulated at 6 h and 24 h (360). Phosphorylated STAT3 at Tyr705 and Ser727 showed different characteristics post-SAH. p-STAT3 at Tyr^705^ had a 2.5-fold enhancement at 2 h after SAH, with a gradual decrease thereafter. Differently, p-STAT3 at Ser^727^ peaked at 1-2 d post-SAH during 1–2 d, then decreased by 7 days (361).

The inhibition of these transcription factors in microglia or astrocytes can significantly improve neurological functions and BBB integrity and mitigate inflammation and immune cell infiltration in animal SAH models. Minocycline is a promising anti-inflammatory, antiapoptotic, and neuroprotective compound for treating various CNS disorders, including SAH (362). Pretreatment with minocycline suppressed microglial/astrocytic activation; downregulated the expression of inflammatory mediators, including S100B, TNF-α, IL-6, iNOS, VCAM-1, ICAM-1, and MMP-9; and repressed TLR4–MyD88 pathway-mediated NF–κB p65 activation. These findings suggest that minocycline modulates neuroinflammatory reactions by interfering with the molecular crosstalk between reactive astrocytes and activated microglia (24). Our group also reported that minocycline relieves neurovascular injury and microglia/astrocyte activation in ICH by mediating multiple signaling pathways, including complement C1q/C3-CR3 signaling (363). Moreover, minocycline significantly modulates the Notch1 signaling pathway (364), TrkB/BDNF pathway (365), and DKK1-Wnt signaling (366) in SAH models. Iron overload in the brain is involved in brain injury after ICH by causing brain edema, neuronal death, and BBB disruption. Minocycline treatment reduced total serum iron and nonheme iron levels in the brain and suppressed ICH-induced upregulation of brain iron-handling proteins and neuronal death (367).

Following SAH, brain cells are exposed to different DAMPs, leading to the concerted activation of multiple inflammasomes, including NLRP3, AIM2, NLRC4, and NLRP1, which mediate the maturation of IL-1β and IL-18 and lead to pyroptosis. Suppressing these inflammasomes can significantly relieve SAH-associated brain injuries (368–371). MCC950 is a selective inhibitor of the NLRP3 inflammasome. MCC950 treatment has neuroprotective effects on SAH by improving the gut microbiota and corticospinal tract (CST) injury (372); relieving neurodeficits, perihematomal brain edema, leukocyte infiltration and microglial activation (373); and preventing early brain injury and delayed cerebral vasospasm (374). Three well-identified neuroprotective agents, resveratrol (375), melatonin (376), and minocycline (377), can prevent SAH-associated brain injury by suppressing NLRP3. Ozanimod, a novel selective S1P receptor modulator, improved the neurological functions of ICH model mice by suppressing microglial and AIM2 inflammasome activation through the regulation of the SIRT3/NF-κB axis (378).

STAT6 and PPAR-γ are two vital microglia-mediated neuroinflammation and efferocytosis factors (379–381). In the ICH model, IL-4-mediated STAT6 signaling activation promoted hematoma resolution and functional recovery (233). The ferroptosis inhibitor ferrostatin-1 (Fer-1) improved neurological function, promoted hematoma absorption, and enhanced the phagocytic function of microglia. Fer-1 mediates M2 polarization of microglia by activating the Fer-1-orchestrating Janus kinase 1/STAT6 pathway (382). Treatment with the PPAR-γ agonist rosiglitazone or PPAR-γ overexpression further elevated PPAR-γ levels in microglia, reduced proinflammatory cytokines, and increased microglial phagocytosis in premature rabbits with intraventricular hemorrhage (383). On the other hand, STAT6 and PPAR-γ also play vital roles in mediating the inflammatory reactions of astrocytes (384, 385). LPS-stimulated microglia promoted “A1” polarization of astrocytes by releasing IL-1α, TNF-α and C1q. Telmisartan, a PPARγ agonist, reversed the microglia-mediated effects on astrocytes by inducing NF-κB p65 downregulation (386). Therefore, targeting STAT6/PPARγ is promising for SAH treatment because it alters microglia and astrocyte activation.

### 5.4 Progress of pharmacological interventions in clinic

Recently, accumulated clinical trials have strongly promoted the development of pharmacological treatments for SAH (387). Several drugs have been shown to have significant pharmacological effects on modulating immune inflammatory responses and the immune microenvironment in the brain, as well as the activation of microglia and astrocytes (**Figure 7**) (388).

Dexmedetomidine (DEX) is an α2-adrenergic agonist widely used for anesthesia, and also anti-inflammation, antioxidation, and anticell death (389). Based on the data from the MIMIC-IV database, DEX was a protective factor for in-hospital mortality of SAH patients (390). Glucocorticoids help to alleviate the inflammatory reaction in CNS diseases (391, 392). Güresir E et al. are conducting a randomized controlled trial to investigate the neuroprotective and anti-inflammatory effects of dexamethasone in treating aSAH patients. In this phase 3 clinical trial, 334 aSAH patients will be enrolled (393). The activation of the complement system plays a crucial role in neuroinflammation following SAH, and it is a key mediator in the pathophysiology of SAH-associated EBI and DCI (394). In a recently started clinical trial (ID. NCT06359782), Dr.Daan de Groot and his colleagues are going to investigate the safety and efficacy of C1-inhibitor Cinryze (an approved inhibitor of the complement system) in treating patients with SAH. In this Phase 2 clinical trial, the inflammatory markers (including TNF-alpha and ILs) in serum and CSF will be measured. In another clinical trial (ID. NCT06579274) enrolling 112 spontaneous SAH patients, the effects of parecoxib (a specific COX-2 inhibitor) in improving the clinical outcome, occurrence of symptomatic vasospasms, as well as occurrence of inflammation will be tested. Those clinical trials will provide more evidence of anti-inflammatory therapy in SAH.

Nimodipine, a lipophilic L-type calcium channel antagonist, is the only proven therapy for vasospasm (387). Nimodipine inhibits spreading depolarization and ischemic injury in mouse live brain slice preparations. Moreover, nimodipine suppressed neuronal damage and directly reduced reactive astrogliosis and microglial activation (395, 396). Anakinra, an IL-1 receptor antagonist (also known as IL-1Ra), can limit brain injury in an experimental SAH model and reduce the levels of inflammatory mediators (214). Anakinra administration contributes to a reduction in IL-6, C-reactive protein, and fibrinogen levels in the blood of SAH patients (397).

Fingolimod is an immunomodulatory agent approved by the Food and Drug Administration (FDA) for treating multiple sclerosis. Studies have shown that fingolimod significantly attenuates SAH-related neurological deficits, brain edema, and neuroinflammation (398, 399). Fingolimod significantly alleviates brain injuries by suppressing neutrophil recruitment, microglia, and astrocyte activation in the brain (400–402). Clinical studies have shown that fingolimod effectively reduces secondary brain injury after ICH by modulating systemic inflammation and protecting vascular permeability (403, 404). Glibenclamide is an orally active ATP-sensitive K^+^ channel (K_ATP_) inhibitor that directly binds and blocks the SUR1 subunits of K_ATP._ Glibenclamide treatment suppressed IL-1β and TNFα and inhibited microglial activation in the cortex of SAH rats (405). Glibenclamide also mitigated cerebral edema by suppressing astrocytic activation (406, 407). Oral administration of glibenclamide significantly reduces cerebral edema in SAH patients (408), mitigates ICH-associated perihematomal edema and improves the 90-day prognosis of patients (409).

Statins, such as simvastatin, may benefit some SAH patients by reducing the incidence of vasospasm and delayed ischemic events (410 –411). In an experimental ICH model, the administration of simvastatin improved hematoma absorption and neurological outcomes. Simvastatin upregulated CD36 expression, facilitated “M2” polarization of microglia and reinforced microglia-induced erythrocyte phagocytosis by inducing PPARγ activation (412). Both simvastatin and atorvastatin significantly reduce microglia and astrocyte activation and suppress IL-1β expression in acute brain injury models (413, 414).

### 5.5 Clinical translational challenges

While multiple animal studies have been widely investigated for treating SAH, their clinical translations remain challenging. Both experimental investigations and clinical observations have demonstrated that SAH inflicts significant damage on the capillary basement membranes and endothelial cells of the BBB. Elevated BBB permeability can be observed at 24-36 hours, peaking at 48 hours, and normalizing on day 3 (415). As a result, the passage of drugs and other substances across this barrier is affected. Depending on the circumstances, the permeability for drug penetration may either decrease, increase, or remain unaltered (416–419). For instance, phenytoin is a routinely prescribed prophylactic antiepileptic following aSAH. aSAH patients (grade 3 and 4) exhibited significantly decreased phenytoin concentration in the brain (420). Altered pharmacokinetics might contribute to unwanted effects of proposed treatments in SAH, which might be associated with clinical challenges in managing aSAH-associated vasospasm. For example, oral nimodipine is the mainstay of pharmacotherapy of cerebral vasospasm and DCI. However, nimodipine-induced hypotension is a serious concern, as blood pressure fluctuations have been associated with the development of focal deficits and worse outcomes (421). The short half-life, acute hypoxemia, armacokinetic variability, drug-drug interactions, and risk of vasoplegia are further shortcomings (422–424). Eicosapentaenoic acid, dapsone and clazosentan showed a good balance between effectiveness and favorable pharmacokinetics (422). Nicardipine and cilostazol, which have longer half-lives than nimodipine, had robust evidence of efficacy and safety (425, 426). Different administration methods, such as localized drug release implants, intracranial or intrathecal administration, can also provide investigational therapies for improving aSAH-associated vasospasm and DCI (427–429).

## 6 Perspectives

The cell–cell crosstalk within the brain is complex. Following ICH, pathological changes result in more complex cell–cell crosstalk in the NVU and neural–glia networks (430). Although the diagnosis and management of SAH have greatly improved in recent decades, substantial morbidity, mortality and burdens of SAH on the healthcare system still exist. Therefore, it is essential to improve current treatment protocols and create novel strategies for managing SAH (431).

Innovative progress in single-cell RNA-sequencing technology has enhanced our understanding of the brain microenvironment at a single-cell level, providing the opportunity to uncover communication between cells and enhance the treatment benefits resulting from complex interactions within the brain (432–434). Recently, scRNA-seq analysis has been conducted on experimental SAH models. For example, the results indicated that during the subacute stage following SAH, reparative microglia infiltrated and expanded clonally in white matter-enriched areas. Moreover, microglia-associated pleiotrophin (PTN) associated with microglia was found to play a role in regulating OPCs in SAH model mice by activating the mTOR signaling pathway. These results highlight the importance of interactions between microglia and OPCs through the PTN pathway, potentially aiding in white matter repair during the subacute phase after SAH (435). Zhang et al. performed a scRNA-seq analysis using perihematomal edema (PHE) from ICH patients. Twelve microglial subsets and 5 neutrophil subsets were identified in PHE tissues. The secreted phosphoprotein-1 (SPP1) pathway provides a foundation for microglial subclusters to communicate with each other as PHE advances. Furthermore, the osteopontin (OPN) produced by microglia can control the immune environment in PHE tissue by interacting with CD44-positive cells (436). Therefore, scRNA-seq analysis could offer more insights into the mechanism of SAH and the development of drugs that target specific cell subtypes.

Cerebral vasospasm is a persistent arterial narrowing typically observed during the 3–14 days following SAH, which is frequently associated with ischemic neurological deficits or even death, resulting in a poor prognosis for patients (437). Circulating immune cells, including neutrophils, lymphocytes and monocytes, as well as immune mediators, such as IL-6, MMP-9 and VEGF, are involved in SAH-associated vasospasm (438–440). For example, our group reported that an elevated neutrophil-to-lymphocyte ratio (NLR) was significantly associated with poorer outcomes and DCI occurrence in SAH patients (441). Following early intracerebral infiltration and peripheral activation of innate immune cells, microglia and astrocytic activation are believed to occur at later time points and then induce secondary neurotoxicity (442, 443). Crosstalk between activated microglia and astrocytes can result in increased neuroinflammatory reactions (24). However, the protective effects of microglia and astrocytes in SAH should also be considered. They can mediate phagocytosis and release anti-inflammatory cytokines, which help with hematoma clearance, BBB repair and neurocognitive recovery (124, 444, 445). A promising strategy for preventing secondary injury involves mediating their crosstalk.

Stem cell therapy is a promising strategy for SAH because it involves multiple functions, including neuroregeneration, the modulation of astrocytes and microglia/macrophage activation, in the lesioned hemisphere (446–448). Notably, stem cell therapy can also mediate astrocyte–microglia crosstalk. Human umbilical cord mesenchymal stem cell (hUC-MSC) administration ameliorated the depression-like behaviors of chronic unpredictable mild stress model (CUMS) mice. hUC-MSCs promoted “M2” polarization while suppressing “M1” polarization of microglia. Moreover, hUC-MSCs inhibited the expression of complement C3a and C3aR in microglia. Thus, hUC-MSCs decrease and alleviate neuronal damage and synaptic deficits by restraining neuroinflammation (449). To overcome the shortcomings of MSC therapy (450), MSC-derived EVs are promising alternatives for SAH treatment because of their low or nonexistent immunogenicity and lack of tumorigenic potential (451). In addition, MSC-EVs have the capability to effectively pass through the BBB. They can also be modified to contain an excess of certain proteins or be filled with specific miRNAs in order to deliver desired therapeutic substances, enhancing their ability to reduce apoptosis and neuroinflammation (447).

Mitochondrial biogenesis, as well as fusion and fission processes, are essential for preserving mitochondrial function and balance. Mitochondrial dysfunctions are hallmarks of SAH and contribute to brain impairments by producing ROS, igniting apoptosis and inducing neuroinflammation (448–454). Mitochondrial transfer to damaged cells can help revive the energy of recipient cells. Accumulating evidence has shown that mitochondrial transplantation can replace impaired or dysfunctional mitochondria and exert significant therapeutic effects against ischemic stroke (455). For example, astrocytic mitochondria can be delivered into adjacent neurons following transient focal cerebral ischemia, and this entry amplifies cell survival signals (456). Microglia share healthy mitochondria with burdened neurons, reducing oxidative stress and normalizing gene expression (457). Astrocytes release both intact mitochondria and humanin, a small bioactive peptide normally transcribed from the mitochondrial genome. With the help of humanin, astrocyte-secreted mitochondria enter microglia, promote PPARγ expression, increase phagocytic activity toward red blood cells and suppress proinflammatory responses. Following ICH, the expression of humanin is significantly reduced. Intravenous administration of humanin reduced neurological deficits and improved hematoma clearance (458). Intravenously transplanted astrocytic mitochondria are transferred into neurons with the help of humanin, thus reducing ICH-associated brain injury (459). Thus, mitochondrial transfer between astrocytes and microglia is also a vital mechanism and therapeutic strategy in SAH.

## 7 Conclusion

The crosstalk between astrocytes and microglia is complex and plays essential roles in the pathological changes that occur during different stages of SAH. In this review, we discuss the effects of SAH-associated DAMPs on microglia and astrocytic activation and the intricate interplay between microglia and astrocytes and summarize the impacts of astrocyte–microglia crosstalk on the NVU as well as circulating immune cells. The extensive bidirectional crosstalk is initially mediated by DAMPs and then mediated by inflammatory cytokines, chemokines, and neurotrophic factors, which are mainly released by microglia/astrocytes and infiltrated immune cells. Moreover, we discuss strategies for SAH treatment by mediating astrocyte and microglia activation and their crosstalk. Further investigations to understand the detailed mechanisms mediating microglia–astrocyte interactions and their impact on the NVU are essential for developing effective therapeutic interventions. Nevertheless, our understanding of microglia/astrocyte communication relies mainly on data derived from research conducted on experimental animal models. Translating preclinical discoveries into the clinical application still faces challenges, partly due to limitations in current SAH animal models and an incomplete understanding of the pathophysiology of this intricate disease. Given that age, sex, and specific medical comorbidities (such as diabetes and obesity) can affect the progression of spontaneous SAH to a considerable extent, it is of great significance to develop SAH animal models that better mimic this clinical disease. Moreover, the systemic inflammation that occurs after SAH is the result of intricate interactions between the nervous and immune systems, impacting all organ systems significantly. Hence, it is crucial to identify and assess the level of systemic inflammation in SAH to identify possible therapeutic opportunities. While past studies have highlighted the benefits of different treatment strategies, more research is needed to fully comprehend the pathophysiological processes of systemic inflammation post aSAH and how they can be applied in clinical practice. Overall, we hope that the above summary can provide a better basis for SAH treatment and stimulate further research.

## Glossary

AIS: acute ischemic stroke
AQP4: aquaporin-4
aSAH: aneurysmal subarachnoid hemorrhage
ATP: adenosine triphosphate
BBB: blood–brain barrier
BDNF: brain-derived neurotrophic factor
CaMKII: calcium calmodulin-dependent kinase II
CCL: Chemokine C-C motif chemokine ligand
CCR2: CC chemokine receptor 2
cGAS: cyclic GMP-AMP synthase
CL: cardiolipin
CNS: central nervous system
COX-1: Cytochrome c oxidase subunit-1
CSF: cerebrospinal fluid
CytB: Cytochrome B
DAMPs: damage-associated molecular patterns
DCI: delayed cerebral ischemia
DEX: Dexmedetomidine
EBI: early brain injury
Ex-mito: Extracellular mitochondria
GFAP: glial fibrillary acidic protein
FAK: focal adhesion kinase
FGF2: fibroblast growth factor 2
FPRs: formyl peptide receptors
GDNF: glial-derived neurotrophic factor
Hb: hemoglobin
HIF-1α: Hypoxia-inducible factor-1α
HO-1: heme oxygenase-1
HSP: heat shock protein
ICAM1: intercellular adhesion molecule-1
ICP: intracranial pressure
IL-6: interleukin-6
iNOS: inducible nitric oxide synthase;
IRFs: Interferon regulatory factors
LCN2: Lipocalin-2
MMPs: matrix metalloproteinases
mtROS: mitochondrial ROS
NET: neutrophil extracellular trap
NF-κB: nuclear factor-kappaB
NK1R: Neurokinin 1 receptor
NVU: neurovascular unit
NLRP3: NACHT, LRR, and PYD domains-containing protein 3
NO: nitric oxide
OxyHbm: oxyhemoglobin;
PRDXs: Peroxiredoxins
RBC: red blood cell
ROS: reactive oxygen species
S1P: sphingosine 1-phosphate
S1PR3: Sphingosine-1-phosphate receptor 3
SAH: subarachnoid hemorrhage
SBI: secondary brain injury
SCI: spinal cord injury
STAT6: Signal Transducer and Activator of Transcription 6
STING: stimulator of interferon genes
TLR: toll-like receptor
TBI: traumatic brain injury
TNF-α: tumour necrosis factor-alpha.

## References

[1] Long B Koyfman A Runyon MS. Subarachnoid hemorrhage: updates in diagnosis and management. Emerg Med Clin North Am. (2017) 35:803–24. doi: 10.1016/j.emc.2017.07.001 28987430

[2] Macdonald RL Schweizer TA. Spontaneous subarachnoid haemorrhage. Lancet. (2017) 389:655–66. doi: 10.1016/S0140-6736(16)30668-7 27637674

[3] Balbi M Vega MJ Lourbopoulos A Terpolilli NA Plesnila N. Long-term impairment of neurovascular coupling following experimental subarachnoid hemorrhage. J Cereb Blood Flow Metab. (2020) 40:1193–202. doi: 10.1177/0271678X19863021 PMC7238370 31296132

[4] Dodd WS Laurent D Dumont AS Hasan DM Jabbour PM Starke RM. Pathophysiology of delayed cerebral ischemia after subarachnoid hemorrhage: A review. J Am Heart Assoc. (2021) 10:e021845. doi: 10.1161/JAHA.121.021845 34325514 PMC8475656

[5] Segarra M Aburto MR Hefendehl J Acker-Palmer A. Neurovascular interactions in the nervous system. Annu Rev Cell Dev Biol. (2019) 35:615–35. doi: 10.1146/annurev-cellbio-100818-125142 31590587

[6] Solár P Zamani A Lakatosová K Joukal M. The blood-brain barrier and the neurovascular unit in subarachnoid hemorrhage: molecular events and potential treatments. Fluids Barriers CNS. (2022) 19:29. doi: 10.1186/s12987-022-00312-4 35410231 PMC8996682

[7] Candelario-Jalil E Dijkhuizen RM Magnus T. Neuroinflammation, stroke, blood-brain barrier dysfunction, and imaging modalities. Stroke. (2022) 53:1473–86. doi: 10.1161/STROKEAHA.122.036946 PMC9038693 35387495

[8] Balbi M Koide M Schwarzmaier SM Wellman GC Plesnila N. Acute changes in neurovascular reactivity after subarachnoid hemorrhage *in vivo* . J Cereb Blood Flow Metab. (2017) 37:178–87. doi: 10.1177/0271678X15621253 PMC5363735 26676226

[9] Balbi M Koide M Wellman GC Plesnila N. Inversion of neurovascular coupling after subarachnoid hemorrhage in *vivo* . J Cereb Blood Flow Metab. (2017) 37:3625–34. doi: 10.1177/0271678X16686595 PMC5669344 28112024

[10] Díaz-Castro B Robel S Mishra A. Astrocyte endfeet in brain function and pathology: open questions. Annu Rev Neurosci. (2023) 46:101–21. doi: 10.1146/annurev-neuro-091922-031205 36854317

[11] Li R Zhao M Yao D Zhou X Lenahan C Wang L. The role of the astrocyte in subarachnoid hemorrhage and its therapeutic implications. Front Immunol. (2022) 13:1008795. doi: 10.3389/fimmu.2022.1008795 36248855 PMC9556431

[12] Anzabi M Ardalan M Iversen NK Rafati AH Hansen B Østergaard L. Hippocampal atrophy following subarachnoid hemorrhage correlates with disruption of astrocyte morphology and capillary coverage by AQP4. Front Cell Neurosci. (2018) 12:19. doi: 10.3389/fncel.2018.00019 29445328 PMC5797792

[13] Wei B Liu W Jin L Huang Y Cheng W Fan H. Hepcidin depending on astrocytic NEO1 ameliorates blood-brain barrier dysfunction after subarachnoid hemorrhage. Cell Death Dis. (2024) 15:569. doi: 10.1038/s41419-024-06909-x 39107268 PMC11303805

[14] Pappas AC Koide M Wellman GC. Astrocyte ca2+ Signaling drives inversion of neurovascular coupling after subarachnoid hemorrhage. J Neurosci. (2015) 35:13375–84. doi: 10.1523/JNEUROSCI.1551-15.2015 PMC4588610 26424885

[15] Schneider UC Davids AM Brandenburg S. Microglia inflict delayed brain injury after subarachnoid hemorrhage. Acta Neuropathol. (2015) 130:215–31. doi: 10.1007/s00401-015-1440-1 25956409

[16] Lauzier DC Athiraman U. Role of microglia after subarachnoid hemorrhage. J Cereb Blood Flow Metab. (2024) 44:841–56. doi: 10.1177/0271678X241237070 PMC11318405 38415607

[17] Heinz R Brandenburg S Nieminen-Kelhä M. Microglia as target for anti-inflammatory approaches to prevent secondary brain injury after subarachnoid hemorrhage (SAH). J Neuroinflammation. (2021) 18:36. doi: 10.1186/s12974-021-02085-3 33516246 PMC7847606

[18] Zheng ZV Lyu H Lam SYE Lam PK Poon WS Wong GKC. The dynamics of microglial polarization reveal the resident neuroinflammatory responses after subarachnoid hemorrhage. Transl Stroke Res. (2020) 11:433–49. doi: 10.1007/s12975-019-00728-5 31628642

[19] Xia DY Yuan JL Jiang XC. SIRT1 promotes M2 microglia polarization via reducing ROS-mediated NLRP3 inflammasome signaling after subarachnoid hemorrhage. Front Immunol. (2021) 12:770744. doi: 10.3389/fimmu.2021.770744 34899720 PMC8653696

[20] Liu H Guo D Wang J. Aloe-emodin from Sanhua Decoction inhibits neuroinflammation by regulating microglia polarization after subarachnoid hemorrhage. J Ethnopharmacol. (2024) 322:117583. doi: 10.1016/j.jep.2023.117583 38122912

[21] Haruwaka K Ikegami A Tachibana Y. Dual microglia effects on blood brain barrier permeability induced by systemic inflammation. Nat Commun. (2019) 10:5816. doi: 10.1038/s41467-019-13812-z 31862977 PMC6925219

[22] Jha MK Jo M Kim JH Suk K. Microglia-astrocyte crosstalk: an intimate molecular conversation. Neuroscientist. (2019) 25:227–40. doi: 10.1177/1073858418783959 29931997

[23] Araki T Ikegaya Y Koyama R. The effects of microglia- and astrocyte-derived factors on neurogenesis in health and disease. Eur J Neurosci. (2021) 54:5880–901. doi: 10.1111/ejn.14969 PMC8451940 32920880

[24] Yang J Wang T Jin X Wang G Zhao F Jin Y. Roles of crosstalk between astrocytes and microglia in triggering neuroinflammation and brain edema formation in 1,2-dichloroethane-intoxicated mice. Cells. (2021) 10:2647. doi: 10.3390/cells10102647 34685627 PMC8534694

[25] Zhu G Wang X Chen L Li Z Li J Xue G. Crosstalk between the oxidative stress and glia cells after stroke: from mechanism to therapies. Front Immunol. (2022) 13:852416. doi: 10.3389/fimmu.2022.852416 35281064 PMC8913707

[26] Luo C Yao J Bi H Wang D Zhu L Su C. Clinical value of inflammatory cytokines in patients with aneurysmal subarachnoid hemorrhage. Clin Interv Aging. (2022) 17:615–26. 10.2147/CIA.S362854 PMC9056097 35502188

[27] Hu X Zhao M Wang M Wang D Zhu L Su C. Elevated serum and cerebrospinal fluid levels of Interleukin-4 related to poor outcome of Aneurysmal subarachnoid hemorrhage. Cytokine. (2024) 184:156780. doi: 10.1016/j.cyto.2024.156780 39432948

[28] Savarraj JPJ Parsha K Hergenroeder GW Zhu L Bajgur SS Ahn S. Systematic model of peripheral inflammation after subarachnoid hemorrhage. Neurology. (2017) 88:1535–45. doi: 10.1212/WNL.0000000000003842 PMC5395070 28314864

[29] Chaudhry SR Güresir E Vatter H Kinfe TM Dietrich D Lamprecht A. Aneurysmal subarachnoid hemorrhage lead to systemic upregulation of IL-23/IL-17 inflammatory axis. Cytokine. (2017) 97:96–103. doi: 10.1016/j.cyto.2017.05.025 28609751

[30] Al-Tamimi YZ Bhargava D Orsi NM Teraifi A Cummings M Ekbote UV. Compartmentalisation of the inflammatory response following aneurysmal subarachnoid haemorrhage. Cytokine. (2019) 123:154778. doi: 10.1016/j.cyto.2019.154778 31323526

[31] Vlachogiannis P Hillered L Enblad P Ronne-Engström E. Elevated levels of several chemokines in the cerebrospinal fluid of patients with subarachnoid hemorrhage are associated with worse clinical outcome. PloS One. (2023) 18:e0282424. doi: 10.1371/journal.pone.0282424 36893189 PMC9997919

[32] Chaudhry SR Kinfe TM Lamprecht A Niemelä M Dobreva G Hänggi D. Elevated level of cerebrospinal fluid and systemic chemokine CCL5 is a predictive biomarker of clinical outcome after aneurysmal subarachnoid hemorrhage (aSAH). Cytokine. (2020) 133:155142. doi: 10.1016/j.cyto.2020.155142 32485621

[33] Pan DS Yan M Hassan M Fang ZB Chen MT. Elevation of serum CXC chemokine ligand-12 levels predicts poor outcome after aneurysmal subarachnoid hemorrhage. J Neurol Sci. (2016) 362:53–8. doi: 10.1016/j.jns.2016.01.024 26944117

[34] Schranz D Molnar T Erdo-Bonyar S Simon D Berki T Zavori L. Fatty acid-binding protein 3 and CXC-chemokine ligand 16 are associated with unfavorable outcome in aneurysmal subarachnoid hemorrhage. J Stroke Cerebrovasc Dis. (2021) 30:106068. doi: 10.1016/j.jstrokecerebrovasdis.2021.106068 34455150

[35] Fountas KN Tasiou A Kapsalaki EZ Paterakis KN Grigorian AA Lee GP. Serum and cerebrospinal fluid C-reactive protein levels as predictors of vasospasm in aneurysmal subarachnoid hemorrhage. Clin article. Neurosurg Focus. (2009) 26:E22. doi: 10.3171/2009.2.FOCUS08311 19409001

[36] Chou SH Feske SK Atherton J Konigsberg RG De Jager PL Du R. Early elevation of serum tumor necrosis factor-α is associated with poor outcome in subarachnoid hemorrhage. J Investig Med. (2012) 60:1054–8. doi: 10.2310/JIM.0b013e3182686932 PMC3740211 22918199

[37] Rasmussen R Bache S Stavngaard T Møller K. Plasma levels of IL-6, IL-8, IL-10, ICAM-1, VCAM-1, IFNγ, and TNFα are not associated with delayed cerebral ischemia, cerebral vasospasm, or clinical outcome in patients with subarachnoid hemorrhage. World Neurosurg. (2019) 128:e1131–6. doi: 10.1016/j.wneu.2019.05.102 31121365

[38] Polin RS Bavbek M Shaffrey ME Billups K Bogaev CA Kassell NF. Detection of soluble E-selectin, ICAM-1, VCAM-1, and L-selectin in the cerebrospinal fluid of patients after subarachnoid hemorrhage. J Neurosurg. (1998) 89:559–67. doi: 10.3171/jns.1998.89.4.0559 9761049

[39] Chou SH Feske SK Simmons SL Konigsberg RG Orzell SC Marckmann A. Elevated peripheral neutrophils and matrix metalloproteinase 9 as biomarkers of functional outcome following subarachnoid hemorrhage. Transl Stroke Res. (2011) 2:600–7. doi: 10.1007/s12975-011-0117-x PMC3236293 22207885

[40] Gong J Zhu Y Yu J Jin J Chen M Liu W. Increased serum interleukin-33 concentrations predict worse prognosis of aneurysmal subarachnoid hemorrhage. Clin Chim Acta. (2018) 486:214–8. doi: 10.1016/j.cca.2018.08.011 30102896

[41] Flood C Akinwunmi J Lagord C Daniel M Berry M Jackowski A. Transforming growth factor-beta1 in the cerebrospinal fluid of patients with subarachnoid hemorrhage: titers derived from exogenous and endogenous sources. J Cereb Blood Flow Metab. (2001) 21:157–62. doi: 10.1097/00004647-200102000-00007 11176281

[42] Fang Y Liu Y Chen L Wang J Zhang J Zhang H. Cerebrospinal fluid markers of neuroinflammation and coagulation in severe cerebral edema and chronic hydrocephalus after subarachnoid hemorrhage: a prospective study. J Neuroinflammation. (2024) 21:237. doi: 10.1186/s12974-024-03236-y 39334416 PMC11438016

[43] Lyubomudrov M Babkina A Tsokolaeva Z Yadgarov M Shigeev S Sundukov D. Morphology of cortical microglia in the hyperacute phase of subarachnoid hemorrhage. Biol (Basel). (2024) 13:917. doi: 10.3390/biology13110917 PMC11591589 39596872

[44] Wang XY Wu F Zhan RY Zhou HJ. Inflammatory role of microglia in brain injury caused by subarachnoid hemorrhage. Front Cell Neurosci. (2022) 16:956185. doi: 10.3389/fncel.2022.956185 36561497 PMC9763450

[45] Hu X Yan J Huang L Araujo C Peng J Gao L. INT-777 attenuates NLRP3-ASC inflammasome-mediated neuroinflammation via TGR5/cAMP/PKA signaling pathway after subarachnoid hemorrhage in rats. Brain Behav Immun. (2024) 119:1021–2. doi: 10.1016/j.bbi.2024.03.011 PMC11162918 38490921

[46] Li R Liu W Yin J Chen Y Guo S Fan H. TSG-6 attenuates inflammation-induced brain injury via modulation of microglial polarization in SAH rats through the SOCS3/STAT3 pathway. J Neuroinflammation. (2018) 15:231. doi: 10.1186/s12974-018-1279-1 30126439 PMC6102893

[47] Peng J Pang J Huang L Enkhjargal B Zhang T Mo J. LRP1 activation attenuates white matter injury by modulating microglial polarization through Shc1/PI3K/Akt pathway after subarachnoid hemorrhage in rats. Redox Biol. (2024) 71:103098. doi: 10.1016/j.redox.2024.103098. Redox Biol. 2019;21:101121. 30703614 PMC6351270

[48] Schallner N Pandit R LeBlanc R 3rd Thomas AJ Ogilvy CS Zuckerbraun BS. Microglia regulate blood clearance in subarachnoid hemorrhage by heme oxygenase-1. J Clin Invest. (2015) 125:2609–25. doi: 10.1016/j.cell.2015.10.067 PMC4563677 26011640

[49] Patsouris V Blecharz-Lang KG Nieminen-Kelhä M Schneider UC Vajkoczy P. Resolution of cerebral inflammation following subarachnoid hemorrhage. Neurocrit Care. (2023) 39:218–28. doi: 10.1007/s12028-023-01770-w PMC10499726 37349601

[50] Zhao Z Nelson AR Betsholtz C Zlokovic BV. Establishment and dysfunction of the blood-brain barrier. Cell. (2015) 163:1064–78. 10.1016/j.cell.2015.10.067 PMC4655822 26590417

[51] Hou C Li J Wang B Liu Q Zhao Y Zhang H. Dynamic evolution of the glymphatic system at the early stages of subarachnoid hemorrhage. Front Neurol. (2022) 13:924080. doi: 10.3389/fneur.2022.924080 35847203 PMC9283644

[52] Feng D Zhou J Liu H Wu X Li F Zhao J. Astrocytic NDRG2-PPM1A interaction exacerbates blood-brain barrier disruption after subarachnoid hemorrhage. Sci Adv. (2022) 8:eabq2423. doi: 10.1126/sciadv.abq2423 36179025 PMC9524825

[53] Petzold A Keir G Kerr M Kay A Kitchen N Smith M. Early identification of secondary brain damage in subarachnoid hemorrhage: a role for glial fibrillary acidic protein. J Neurotrauma. (2006) 23:1179–84. doi: 10.1089/neu.2006.23.1179 16866629

[54] Coulibaly AP Provencio JJ. Aneurysmal subarachnoid hemorrhage: an overview of inflammation-induced cellular changes. Neurotherapeutics. (2020) 17:436–45. doi: 10.1007/s13311-019-00829-x PMC7283430 31907877

[55] Kooijman E Nijboer CH van Velthoven CT Mol W Dijkhuizen RM Kesecioglu J. Long-term functional consequences and ongoing cerebral inflammation after subarachnoid hemorrhage in the rat. PloS One. (2014) 9:e90584. doi: 10.1371/journal.pone.0090584 24603553 PMC3946189

[56] Alsbrook DL Di Napoli M Bhatia K Biller J Andalib S Hinduja A. Neuroinflammation in acute ischemic and hemorrhagic stroke. Curr Neurol Neurosci Rep. (2023) 23:407–31. doi: 10.1007/s11910-023-01282-2 PMC10544736 37395873

[57] van Dijk BJ Vergouwen MD Kelfkens MM Rinkel GJ Hol EM. Glial cell response after aneurysmal subarachnoid hemorrhage - Functional consequences and clinical implications. Biochim Biophys Acta. (2016) 1862:492–505. doi: 10.1016/j.bbadis.2015.10.013 26493445

[58] Zhou HJ Yang X Cui HJ Tang T Zhong JH Luo JK. Leukemia Inhibitory Factor Contributes to Reactive Astrogliosis via Activation of Signal Transducer and Activator of Transcription 3 Signaling after Intracerebral Hemorrhage in Rats. J Neurotrauma. (2017) 34:1658–65. doi: 10.1089/neu.2016.4711 27825285

[59] Denning NL Aziz M Gurien SD Wang P. DAMPs and NETs in sepsis. Front Immunol. (2019) 10:2536. doi: 10.3389/fimmu.2019.02536 31736963 PMC6831555

[60] Frank MG Weber MD Watkins LR Maier SF. Stress sounds the alarmin: The role of the danger-associated molecular pattern HMGB1 in stress-induced neuroinflammatory priming. Brain Behav Immun. (2015) 48:1–7. doi: 10.1016/j.bbi.2015.03.010 25816800 PMC4508196

[61] Wang P Zuo H Shi H Wang Z Ren X Shi J. Gastrodin inhibits reactive astrocyte-mediated inflammation in hypoxic-ischemic brain damage through S100B/RAGE-Smad3 signaling. Acta Biochim Biophys Sin (Shanghai). (2025). doi: 10.3724/abbs.2024235 PMC12247138 40055915

[62] Laird MD Shields JS Sukumari-Ramesh S Kimbler DE Fessler RD Shakir B. High mobility group box protein-1 promotes cerebral edema after traumatic brain injury via activation of toll-like receptor 4. Glia. (2014) 62:26–38. doi: 10.1002/glia.22581 24166800 PMC4503251

[63] Gülke E Gelderblom M Magnus T. Danger signals in stroke and their role on microglia activation after ischemia. Ther Adv Neurol Disord. (2018) 11:1756286418774254. doi: 10.1177/1756286418774254 29854002 PMC5968660

[64] Schädlich IS Winzer R Stabernack J Tolosa E Magnus T Rissiek B. The role of the ATP-adenosine axis in ischemic stroke. Semin Immunopathol. (2023) 45:347–65. doi: 10.1007/s00281-023-00987-3 PMC10279578 36917241

[65] Balança B Desmurs L Grelier J Perret-Liaudet A Lukaszewicz AC. DAMPs and RAGE pathophysiology at the acute phase of brain injury: an overview. Int J Mol Sci. (2021) 22:2439. doi: 10.3390/ijms22052439 33670976 PMC7957733

[66] Fang P Schachner M Shen YQ. HMGB1 in development and diseases of the central nervous system. Mol Neurobiol. (2012) 45:499–506. doi: 10.1007/s12035-012-8264-y 22580958

[67] Chaudhry SR Hafez A Rezai Jahromi B Kinfe TM Lamprecht A Niemelä M. Role of damage associated molecular pattern molecules (DAMPs) in aneurysmal subarachnoid hemorrhage (aSAH). Int J Mol Sci. (2018) 19:2035. doi: 10.3390/ijms19072035 30011792 PMC6073937

[68] Chou SH Lan J Esposito E Ning M Balaj L Ji X. Extracellular mitochondria in cerebrospinal fluid and neurological recovery after subarachnoid hemorrhage. Stroke. (2017) 48:2231–7. doi: 10.1161/STROKEAHA.117.017758 PMC5526718 28663512

[69] Chaudhry SR Frede S Seifert G Kinfe TM Niemelä M Lamprecht A. Temporal profile of serum mitochondrial DNA (mtDNA) in patients with aneurysmal subarachnoid hemorrhage (aSAH). Mitochondrion. (2019) 47:218–26. doi: 10.1016/j.mito.2018.12.001 30529453

[70] Wang HC Yang TM Lin WC Lin YJ Tsai NW Liou CW. The value of serial plasma and cerebrospinal fluid nuclear and mitochondrial deoxyribonucleic acid levels in aneurysmal subarachnoid hemorrhage. J Neurosurg. (2013) 118:13–9. doi: 10.3171/2012.8.JNS112093 23020765

[71] Sokół B Woźniak A Jankowski R Jurga S Wąsik N Shahid H. HMGB1 level in cerebrospinal fluid as a marker of treatment outcome in patients with acute hydrocephalus following aneurysmal subarachnoid hemorrhage. J Stroke Cerebrovasc Dis. (2015) 24:1897–904. doi: 10.1016/j.jstrokecerebrovasdis.2015.05.002 26047599

[72] Chu XH Hu HY Godje ISG Zhu LJ Zhu JB Feng YL. Elevated HMGB1 and sRAGE levels in cerebrospinal fluid of aneurysmal subarachnoid hemorrhage patients. J Stroke Cerebrovascular Dis. (2023) 32:107061. doi: 10.1016/j.jstrokecerebrovasdis.2023.107061 36871437

[73] Wang KC Tang SC Lee JE Li YI Huang YS Yang WS. Cerebrospinal fluid high mobility group box 1 is associated with neuronal death in subarachnoid hemorrhage. J Cereb Blood Flow Metab. (2017) 37:435–43. doi: 10.1177/0271678X16629484 PMC5381442 26823474

[74] Zhu XD Chen JS Zhou F Liu QC Chen G Zhang JM. Relationship between plasma high mobility group box-1 protein levels and clinical outcomes of aneurysmal subarachnoid hemorrhage. J Neuroinflammation. (2012) 9:194. doi: 10.1186/1742-2094-9-194 22883976 PMC3424135

[75] Zhou Y Xiong KL Lin S Zhong Q Lu FL Liang H. Elevation of high-mobility group protein box-1 in serum correlates with severity of acute intracerebral hemorrhage. Mediators Inflamm. (2010) 2010:142458. doi: 10.1155/2010/142458 20936104 PMC2948906

[76] Kellermann I Kleindienst A Hore N Buchfelder M Brandner S. Early CSF and serum S100B concentrations for outcome prediction in traumatic brain injury and subarachnoid hemorrhage. Clin Neurol Neurosurg. (2016) 145:79–83. doi: 10.1016/j.clineuro.2016.04.005 27101088

[77] Moritz S Warnat J Bele S Graf BM Woertgen C. The prognostic value of NSE and S100B from serum and cerebrospinal fluid in patients with spontaneous subarachnoid hemorrhage. J Neurosurg Anesthesiol. (2010) 22:21–31. doi: 10.1097/ANA.0b013e3181bdf50d 20027011

[78] Balança B Ritzenthaler T Gobert F Richet C Bodonian C Carrillon R. Significance and diagnostic accuracy of early S100B serum concentration after aneurysmal subarachnoid hemorrhage. J Clin Med. (2020) 9:1746. doi: 10.3390/jcm9061746 32516898 PMC7356310

[79] Zhou Z Zeng J Yu S Zhao Y Yang X Zhou Y. Neurofilament light chain and S100B serum levels are associated with disease severity and outcome in patients with aneurysmal subarachnoid hemorrhage. Front Neurol. (2022) 13:956043. doi: 10.3389/fneur.2022.956043 35989914 PMC9381989

[80] Naidech AM Jovanovic B Wartenberg KE Parra A Ostapkovich N Connolly ES. Higher hemoglobin is associated with improved outcome after subarachnoid hemorrhage. Crit Care Med. (2007) 35:2383–9. doi: 10.1097/01.CCM.0000284516.17580.2C 17717494

[81] Kramer AH Zygun DA Bleck TP Dumont AS Kassell NF Nathan B. Relationship between hemoglobin concentrations and outcomes across subgroups of patients with aneurysmal subarachnoid hemorrhage. Neurocrit Care. (2009) 10:157–65. doi: 10.1007/s12028-008-9171-y 19116699

[82] Naidech AM Drescher J Ault ML Shaibani A Batjer HH Alberts MJ. Higher hemoglobin is associated with less cerebral infarction, poor outcome, and death after subarachnoid hemorrhage. Neurosurgery. (2006) 59:775–80. doi: 10.1227/01.NEU.0000232662.86771.A9 17038943

[83] Wang C Kou Y Han Y Li X. Early serum calprotectin (S100A8/A9) predicts delayed cerebral ischemia and outcomes after aneurysmal subarachnoid hemorrhage. J Stroke Cerebrovasc Dis. (2020) 29:104770. doi: 10.1016/j.jstrokecerebrovasdis.2020.104770 32173226

[84] Qiu SZ Zheng GR Ma CY Chen B Huang JJ Huang G. High serum S100A12 levels predict poor outcome after acute primary intracerebral hemorrhage. Neuropsychiatr Dis Treat. (2021) 17:3245–53. doi: 10.2147/NDT.S337041 PMC8572103 34754192

[85] Garland P Durnford AJ Okemefuna AI Dunbar J Nicoll JA Galea J. Heme-hemopexin scavenging is active in the brain and associates with outcome after subarachnoid hemorrhage. Stroke. (2016) 47:872–6. doi: 10.1161/STROKEAHA.115.011956 26768209

[86] Petzold A Worthington V Appleby I Kerr ME Kitchen N Smith M. Cerebrospinal fluid ferritin level, a sensitive diagnostic test in late-presenting subarachnoid hemorrhage. J Stroke Cerebrovasc Dis. (2011) 20:489–93. doi: 10.1016/j.jstrokecerebrovasdis.2010.02.021 PMC3023836 20719531

[87] Ohnishi M Monda A Takemoto R Fujimoto Y Sugitani M Iwamura T. High-mobility group box 1 up-regulates aquaporin 4 expression via microglia-astrocyte interaction. Neurochem Int. (2014) 75:32–8. doi: 10.1016/j.neuint.2014.05.007 24893328

[88] Rosciszewski G Cadena V Auzmendi J Cieri MB Lukin J Rossi AR. Detrimental effects of HMGB-1 require microglial-astroglial interaction: implications for the status epilepticus-induced neuroinflammation. Front Cell Neurosci. (2019) 13:380. doi: 10.3389/fncel.2019.00380 31507379 PMC6718475

[89] Chi G Lu J He T Wang Y Zhou X Zhang Y. High mobility group box-1 protein promotes astrocytic CCL5 production through the MAPK/NF-κB pathway following spinal cord injury. Sci Rep. (2024) 14:22344. doi: 10.1038/s41598-024-72947-2 39333662 PMC11437233

[90] Wang Z Yuan B Fu F Huang S Yang Z. Hemoglobin enhances miRNA-144 expression and autophagic activation mediated inflammation of microglia via mTOR pathway. Sci Rep. (2017) 7:11861. doi: 10.1038/s41598-017-12067-2 28928406 PMC5605685

[91] Sayeed MSB Alhadidi Q Shah ZA. Cofilin signaling in hemin-induced microglial activation and inflammation. J Neuroimmunology. (2017) 313:46–55. doi: 10.1016/j.jneuroim.2017.10.007 29153608 PMC11956890

[92] Wei X Zhang F Cheng D Wang Z Xing N Yuan J. Free heme induces neuroinflammation and cognitive impairment by microglial activation via the TLR4/MyD88/NF-κB signaling pathway. Cell Communication Signaling. (2024) 22:16. doi: 10.1186/s12964-023-01387-8 38183122 PMC10768134

[93] Pan H Wang H Zhu L Mao L Qiao L Su X. Depletion of Nrf2 enhances inflammation induced by oxyhemoglobin in cultured mice astrocytes. Neurochem Res. (2011) 36:2434–41. doi: 10.1007/s11064-011-0571-6 21833844

[94] Gram M Sveinsdottir S Ruscher K Hansson SR Cinthio M Akerström B. Hemoglobin induces inflammation after preterm intraventricular hemorrhage by methemoglobin formation. J Neuroinflammation. (2013) 10:100. doi: 10.1186/1742-2094-10-100 23915174 PMC3750409

[95] Kong L Li W Chang E Wang W Shen N Xu X. mtDNA-STING axis mediates microglial polarization *via* IRF3/NF-κB signaling after ischemic stroke. Front Immunol. (2022) 13:860977. doi: 10.3389/fimmu.2022.860977 35450066 PMC9017276

[96] Shi W Zhou Q Lu L Zhang Y Zhang H Pu Y. Copper induced cytosolic escape of mitochondrial DNA and activation of cGAS-STING-NLRP3 pathway-dependent pyroptosis in C8-D1A cells. Ecotoxicol Environ Saf. (2024) 285:117085. doi: 10.1016/j.ecoenv.2024.117085 39321529

[97] Bianchi R Kastrisianaki E Giambanco I Donato R. S100B protein stimulates microglia migration via RAGE-dependent up-regulation of chemokine expression and release. J Biol Chem. (2011) 286:7214–26. doi: 10.1074/jbc.M110.169342 PMC3044978 21209080

[98] Bianchi R Giambanco I Donato R. S100B/RAGE-dependent activation of microglia via NF-kappaB and AP-1 Co-regulation of COX-2 expression by S100B, IL-1beta and TNF-alpha. Neurobiol Aging. (2010) 31:665–77. doi: 10.1016/j.neurobiolaging.2008.05.017 18599158

[99] Hou JY Zhou XL Wang XY Liang J Xue Q. Peroxiredoxin-6 released by astrocytes contributes to neuroapoptosis during ischemia. Neuroscience. (2023) 512:59–69. doi: 10.1016/j.neuroscience.2023.01.003 36642396

[100] Peng L Ji Y Li Y You Y Zhou Y. PRDX6-iPLA2 aggravates neuroinflammation after ischemic stroke via regulating astrocytes-induced M1 microglia. Cell Commun Signal. (2024) 22:76. doi: 10.1186/s12964-024-01476-2 38287382 PMC10823689

[101] Qiu J Nishimura M Wang Y Sims JR Qiu S Savitz SI. Early release of HMGB-1 from neurons after the onset of brain ischemia. J Cereb Blood Flow Metab. (2008) 28:927–38. doi: 10.1038/sj.jcbfm.9600582 18000511

[102] Nakahara T Tsuruta R Kaneko T Yamashita S Fujita M Kasaoka S. High-mobility group box 1 protein in CSF of patients with subarachnoid hemorrhage. Neurocrit Care. (2009) 11:362–8. doi: 10.1007/s12028-009-9276-y 19777384

[103] Sun Q Wu W Hu YC Li H Zhang D Li S. Early release of high-mobility group box 1 (HMGB1) from neurons in experimental subarachnoid hemorrhage *in vivo* and *in vitro* . J Neuroinflamm. (2014) 11:106. doi: 10.1186/1742-2094-11-106 PMC4107626 24924349

[104] Murakami K Koide M Dumont TM Russell SR Tranmer BI Wellman GC. Subarachnoid hemorrhage induces gliosis and increased expression of the pro-inflammatory cytokine high mobility group box 1 protein. Transl Stroke Res. (2011) 2:72–9. doi: 10.1007/s12975-010-0052-2 PMC3072171 21479116

[105] Yu C Huang Y Xie J Duan C Liu S Zhao W. HMGB1 released from pyroptotic vascular endothelial cells promotes immune disorders in exertional heatstroke. Int J Hyperthermia. (2024) 41:2378867. doi: 10.1080/02656736.2024.2378867 39117343

[106] Gao X Zhao X Li J Liu C Li W Zhao J. Neutrophil extracellular traps mediated by platelet microvesicles promote thrombosis and brain injury in acute ischemic stroke. Cell Communication Signaling. (2024) 22:50. doi: 10.1186/s12964-023-01379-8 38233928 PMC10795390

[107] Lotze MT Tracey KJ. High-mobility group box 1 protein (HMGB1): Nuclear weapon in the immune arsenal. Nat Rev Immunol. (2005) 5:331–42. doi: 10.1038/nri1594 15803152

[108] Qiu J Xu J Zheng Y Wei Y Zhu X Lo EH. High-mobility group box 1 promotes metalloproteinase-9 upregulation through Toll-like receptor 4 after cerebral ischemia. Stroke. (2010) 41:2077–82. 10.1161/STROKEAHA.110.590463 PMC3066477 20671243

[109] Li H Wu W Sun Q Liu M Li W Zhang XS. Expression and cell distribution of receptor for advanced glycation end-products in the rat cortex following experimental subarachnoid hemorrhage. Brain Res. (2014) 1543:315–23. doi: 10.1016/j.brainres.2013.11.023 24291745

[110] Xu S Mei S Lu J Wu H Dong X Shi L. Transcriptome analysis of microglia reveals that the TLR2/IRF7 signaling axis mediates neuroinflammation after subarachnoid hemorrhage. Front Aging Neurosci. (2021) 13:645649. doi: 10.3389/fnagi.2021.645649 34276335 PMC8278202

[111] Hanafy KA. The role of microglia and the TLR4 pathway in neuronal apoptosis and vasospasm after subarachnoid hemorrhage. J Neuroinflammation. (2013) 10:83. doi: 10.1186/1742-2094-10-83 23849248 PMC3750560

[112] Bozza MT Jeney V. Pro-inflammatory actions of heme and other hemoglobin-derived DAMPs. Front Immunol. (2020) 11:1323. doi: 10.3389/fimmu.2020.01323 32695110 PMC7339442

[113] Stein M Brokmeier L Herrmann J Scharbrodt W Schreiber V Bender M. Mean hemoglobin concentration after acute subarachnoid hemorrhage and the relation to outcome, mortality, vasospasm, and brain infarction. J Clin Neurosci. (2015) 22:530–4. doi: 10.1016/j.jocn.2014.08.026 25533213

[114] Akeret K Buzzi RM Schaer CA Thomson BR Vallelian F Wang S. Cerebrospinal fluid hemoglobin drives subarachnoid hemorrhage-related secondary brain injury. J Cereb Blood Flow Metab. (2021) 41:3000–15. doi: 10.1177/0271678X211020629 PMC8545037 34102922

[115] Balla G Jacob HS Eaton JW Belcher JD Vercellotti GM. Hemin: a possible physiological mediator of low density lipoprotein oxidation and endothelial injury. Arterioscler Thromb. (1991) 11:1700–11. doi: 10.1161/01.ATV.11.6.1700 1931871

[116] Suzuki S Kassell NF Lee KS. Hemin activation of an inducible isoform of nitric oxide synthase in vascular smooth-muscle cells. J Neurosurg. (1995) 83:862–6. doi: 10.3171/jns.1995.83.5.0862 7472555

[117] Li Q Chen Y Li B Luo C Zuo S Liu X. Hemoglobin induced NO/cGMP suppression Deteriorate Microcirculation via Pericyte Phenotype Transformation after Subarachnoid Hemorrhage in Rats. Sci Rep. (2024) 14:13284. doi: 10.1038/s41598-024-64285-0 38858541 PMC11164870

[118] Galea I Bandyopadhyay S Bulters D Humar R Hugelshofer M Schaer DJ. Haptoglobin treatment for aneurysmal subarachnoid hemorrhage: review and expert consensus on clinical translation. Stroke. (2023) 54:1930–42. doi: 10.1161/STROKEAHA.123.040205 PMC10289236 37232189

[119] Garland P Morton MJ Haskins W Zolnourian A Durnford A Gaastra B. Haemoglobin causes neuronal damage *in vivo* which is preventab le by haptoglobin. Brain Commun. (2020) 2:fcz053. doi: 10.1093/braincomms/fcz053 32346673 PMC7188517

[120] Imai T Iwata S Hirayama T Nagasawa H Nakamura S Shimazawa M. Intracellular Fe2+ accumulation in endothelial cells and pericytes induces blood-brain barrier dysfunction in secondary brain injury after brain hemorrhage. Sci Rep. (2019) 9:6228. doi: 10.1038/s41598-019-42370-z 30996325 PMC6470176

[121] Lara FA Kahn SA da Fonseca AC Bahia CP Pinho JP Graca-Souza AV. On the fate of extracellular hemoglobin and heme in brain. J Cereb Blood Flow Metab. (2009) 29:1109–20. doi: 10.1038/jcbfm.2009.34 19337276

[122] Rollins S Perkins E Mandybur G Zhang JH. Oxyhemoglobin produces necrosis, not apoptosis, in astrocytes. Brain Res. (2002) 945:41–9. doi: 10.1016/S0006-8993(02)02562-3 12113950

[123] Liu GJ Tao T Wang H Zhou Y Gao X Gao YY. Functions of resolvin D1-ALX/FPR2 receptor interaction in the hemoglobin-induced microglial inflammatory response and neuronal injury. J Neuroinflamm. (2020) 17:1–17. doi: 10.1186/s12974-020-01918-x PMC7429751 32795323

[124] Li Q Lan X Han X Durham F Wan J Weiland A. Microglia-derived interleukin-10 accelerates post-intracerebral hemorrhage hematoma clearance by regulating CD36. Brain Behav Immun. (2021) 94:437–57. doi: 10.1016/j.bbi.2021.02.001 PMC8058329 33588074

[125] Holfelder K Schittenhelm J Trautmann K Haybaeck J Meyermann R Beschorner R. *De novo* expression of the hemoglobin scavenger receptor CD163 by activated microglia is not associated with hemorrhages in human brain lesions. Histol Histopathol. (2011) 26:1007–17. doi: 10.14670/HH-26.1007 21692033

[126] Garton T Keep RF Hua Y Xi G. CD163, a hemoglobin/haptoglobin scavenger receptor, after intracerebral hemorrhage: functions in microglia/macrophages versus neurons. Transl Stroke Res. (2017) 8:612–6. doi: 10.1007/s12975-017-0535-5 28386733

[127] Leclerc JL Lampert AS Loyola Amador C Schlakman B Vasilopoulos T Svendsen P. The absence of the CD163 receptor has distinct temporal influences on intracerebral hemorrhage outcomes. J Cereb Blood Flow Metab. (2018) 38:262–73. doi: 10.1177/0271678X17701459 PMC5951015 28358264

[128] You M Long C Wan Y Guo H Shen J Li M. Neuron derived fractalkine promotes microglia to absorb hematoma via CD163/HO-1 after intracerebral hemorrhage. Cell Mol Life Sci. (2022) 79:224. doi: 10.1007/s00018-022-04212-6 35389112 PMC11072118

[129] Chen X He X Xu F Xu N Sharifi NH Zhang P. Fractalkine enhances hematoma resolution and improves neurological function via CX3CR1/AMPK/PPARγ Pathway after GMH. Stroke. (2023) 54:2420–33. doi: 10.1161/STROKEAHA.123.043005 PMC10453335 37465997

[130] Yang Y Ren J Sun Y Xue Y Zhang Z Gong A. A connexin43/YAP axis regulates astroglial-mesenchymal transition in hemoglobin induced astrocyte activation. Cell Death Differ. (2018) 25:1870–84. doi: 10.1038/s41418-018-0137-0 PMC6180064 29880858

[131] Yang Y Xi Z Xue Y Ren J Sun Y Wang B. Hemoglobin pretreatment endows rat cortical astrocytes resistance to hemin-induced toxicity via Nrf2/HO-1 pathway. Exp Cell Res. (2017) 361:217–24. doi: 10.1016/j.yexcr.2017.10.020 29074371

[132] Fang Y Wang X Lu J Shi H Huang L Shao A. Inhibition of caspase-1-mediated inflammasome activation reduced blood coagulation in cerebrospinal fluid after subarachnoid haemorrhage. EBioMedicine. (2022) 76:103843. doi: 10.1016/j.ebiom.2022.103843 35101655 PMC8822177

[133] Chen XX Tao T Gao S Wang H Zhou XM Gao YY. Knock-down of CD24 in astrocytes aggravates oxyhemoglobin-induced hippocampal neuron impairment. Neurochem Res. (2022) 47:1123. doi: 10.1007/s11064-021-03525-5. Neurochem Res. 2024 Aug;49(8):2271-2272. doi: 10.1007/s11064-021-03468-x 34665391

[134] Sukumari-Ramesh S Laird MD Singh N Vender JR Alleyne CH Jr Dhandapani KM. Astrocyte-derived glutathione attenuates hemin-induced apoptosis in cerebral microvascular cells. Glia. (2010) 58:1858–70. doi: 10.1002/glia.21055 PMC2951495 20737478

[135] Endo M Tanaka Y Fukuoka M Suzuki H Minami Y. Wnt5a/Ror2 promotes Nrf2-mediated tissue protective function of astrocytes after brain injury. Glia. (2024) 72:411–32. doi: 10.1002/glia.24483 37904612

[136] Garton T Keep RF Hua Y Xi G. Brain iron overload following intracranial haemorrhage. Stroke Vasc Neurol. (2016) 1:172–84. doi: 10.1136/svn-2016-000042 PMC5435218 28959481

[137] Eisenstein RS Garcia-Mayol D Pettingell W Munro HN. Regulation of ferritin and heme oxygenase synthesis in rat fibroblasts by different forms of iron. Proc Natl Acad Sci USA. (1991) 88:688–92. doi: 10.1073/pnas.88.3.688 PMC50878 1992460

[138] DeGregorio-Rocasolano N Martí-Sistac O Gasull T. Deciphering the iron side of stroke: neurodegeneration at the crossroads between iron dyshomeostasis, excitotoxicity, and ferroptosis. Front Neurosci. (2019) 13:85. doi: 10.3389/fnins.2019.00085 30837827 PMC6389709

[139] Galea I Durnford A Glazier J Mitchell S Kohli S Foulkes L. Iron deposition in the brain after aneurysmal subarachnoid hemorrhage. Stroke. (2022) 53:1633–42. doi: 10.1161/STROKEAHA.121.036645 35196874

[140] Vela D. Hepcidin, an emerging and important player in brain iron homeostasis. J Transl Med. (2018) 16:25. doi: 10.1186/s12967-018-1399-5 29415739 PMC5803919

[141] Gao SQ Wang X Li T Gao CC Han YL Qiu JY. Astrocyte-derived hepcidin aggravates neuronal iron accumulation after subarachnoid hemorrhage by decreasing neuronal ferroportin1. Free Radic Biol Med. (2024) 210:318–32. doi: 10.1016/j.freeradbiomed.2023.11.036 38052274

[142] Xiong XY Liu L Wang FX Yang YR Hao JW Wang PF. Toll-like receptor 4/myD88-mediated signaling of hepcidin expression causing brain iron accumulation, oxidative injury, and cognitive impairment after intracerebral hemorrhage. Circulation. (2016) 134:1025–38. doi: 10.1161/CIRCULATIONAHA.116.021881 27576776

[143] Zhang Q Raoof M Chen Y Sumi Y Sursal T Junger W. Circulating mitochondrial DAMPs cause inflammatory responses to injury. Nature. (2010) 464:104–7. doi: 10.1038/nature08780 PMC2843437 20203610

[144] Zhou R Yazdi AS Menu P Tschopp J. A role for mitochondria in NLRP3 inflammasome activation. Nature. (2011) 475:122. *Nature.* 2011;469(7329):221-225. doi: 10.1038/nature09663 21124315

[145] Zhu M Barbas AS Lin L Scheuermann U Bishawi M Brennan TV. Mitochondria released by apoptotic cell death initiate innate immune responses. Immunohorizons. (2019) 3:26–7. doi: 10.4049/immunohorizons.1800089. *Immunohorizons.* 2018;2(11):384-397. PMC6400482 30847435

[146] Wenceslau CF Szasz T McCarthy CG Baban B NeSmith E Webb RC. Mitochondrial N-formyl peptides cause airway contraction and lung neutrophil infiltration via formyl peptide receptor activation. Pulm Pharmacol Ther. (2016) 37:49–56. doi: 10.1016/j.pupt.2016.02.005 26923940 PMC9731398

[147] Nasi M De Gaetano A Bianchini E De Biasi S Gibellini L Neroni A. Mitochondrial damage-associated molecular patterns stimulate reactive oxygen species production in human microglia. Mol Cell Neurosci. (2020) 108:103538. doi: 10.1016/j.mcn.2020.103538 32828963

[148] Li Z Li Y Han J Zhu Z Li M Liu Q. Formyl peptide receptor 1 signaling potentiates inflammatory brain injury. Sci Transl Med. (2021) 13:eabe9890. doi: 10.1126/scitranslmed.abe9890 34349037

[149] Falabella M Vernon HJ Hanna MG Claypool SM Pitceathly RDS. Cardiolipin, mitochondria, and neurological disease. Trends Endocrinol Metab. (2021) 32:224–37. doi: 10.1016/j.tem.2021.01.006 PMC8277580 33640250

[150] Pointer CB Wenzel TJ Klegeris A. Extracellular cardiolipin regulates select immune functions of microglia and microglia-like cells. Brain Res Bull. (2019) 146:153–63. doi: 10.1016/j.brainresbull.2019.01.002 30625370

[151] Zhao Z Wang M Tian Y Hilton T Salsbery B Zhou EZ. Cardiolipin-mediated procoagulant activity of mitochondria contributes to traumatic brain injury-associated coagulopathy in mice. Blood. (2016) 127:2763–72. 10.1182/blood-2015-12-688838 PMC4891956 27002118

[152] Chao H Lin C Zuo Q Liu Y Xiao M Xu X. Cardiolipin-dependent mitophagy guides outcome after traumatic brain injury. J Neurosci. (2019) 39:1930–43. doi: 10.1523/JNEUROSCI.3415-17.2018 PMC6407296 30626699

[153] Youn DH Kim BJ Kim Y Jeon JP. Extracellular mitochondrial dysfunction in cerebrospinal fluid of patients with delayed cerebral ischemia after aneurysmal subarachnoid hemorrhage. Neurocrit Care. (2020) 33:422–8. doi: 10.1007/s12028-019-00895-1 31898178

[154] Zhang C Liu C Li F Zheng M Liu Y Li L. Extracellular mitochondria activate microglia and contribute to neuroinflammation in traumatic brain injury. Neurotox Res. (2022) 40:2264–77. doi: 10.1007/s12640-022-00566-8 36087194

[155] Foote K Reinhold J Yu EPK Figg NL Finigan A Murphy MP. Restoring mitochondrial DNA copy number preserves mitochondrial function and delays vascular aging in mice. Aging Cell. (2018) 17:e12773. doi: 10.1111/acel.12773 29745022 PMC6052475

[156] Zhong Z Liang S Sanchez-Lopez E He F Shalapour S Lin XJ. New mitochondrial DNA synthesis enables NLRP3 inflammasome activation. Nature. (2018) 560:198–203. doi: 10.1038/s41586-018-0372-z 30046112 PMC6329306

[157] Jabir MS Hopkins L Ritchie ND Ullah I Bayes HK Li D. Mitochondrial damage contributes to Pseudomonas aeruginosa activation of the inflammasome and is downregulated by autophagy. Autophagy. (2015) 11:166–82. doi: 10.4161/15548627.2014.981915 PMC4502769 25700738

[158] Próchnicki T Vasconcelos MB Robinson KS Mangan MSJ De Graaf D Shkarina K. Mitochondrial damage activates the NLRP10 inflammasome. Nat Immunol. (2023) 24:595–603. doi: 10.1038/s41590-023-01451-y 36941400

[159] Yu CH Davidson S Harapas CR Hilton JB Mlodzianoski MJ Laohamonthonkul P. TDP-43 Triggers Mitochondrial DNA Release via mPTP to Activate cGAS/STING in ALS. Cell. (2020) 183:636–649.e18. doi: 10.1016/j.cell.2020.09.020 33031745 PMC7599077

[160] Quan S Fu X Cai H Ren Z Xu Y Jia L. The neuroimmune nexus: unraveling the role of the mtDNA-cGAS-STING signal pathway in Alzheimer’s disease. Mol Neurodegener. (2025) 20:25. doi: 10.1186/s13024-025-00815-2 40038765 PMC11877805

[161] Cho Y Tachibana S Lam K Arita Y Khosrowjerdi S Zhang O. Perm1 promotes cardiomyocyte mitochondrial biogenesis and protects against hypoxia/reoxygenation-induced damage in mice. J Biol Chem. (2021) 297:101121. doi: 10.1016/j.jbc.2021.101121 34464737 PMC8408517

[162] Li Q Yang L Wang K Chen Z Liu H Yang X. Oxidized mitochondrial DNA activates the cGAS-STING pathway in the neuronal intrinsic immune system after brain ischemia-reperfusion injury. Neurotherapeutics. (2024) 21:e00368. doi: 10.1016/j.neurot.2024.e00368 38688786 PMC11284550

[163] Wei FL Wang TF Wang CL Zhang ZP Zhao JW Heng W. Cytoplasmic Escape of Mitochondrial DNA Mediated by Mfn2 Downregulation Promotes Microglial Activation via cGas-Sting Axis in Spinal Cord Injury. Adv Sci (Weinh). (2024) 11:e2305442. doi: 10.1002/advs.202305442 38009491 PMC10811505

[164] Gu F Wang Z Ding H Tao X Zhang J Dai K. Microglial mitochondrial DNA release contributes to neuroinflammation after intracerebral hemorrhage through activating AIM2 inflammasome. Exp Neurol. (2024) 382:114950. doi: 10.1016/j.expneurol.2024.114950 39278588

[165] Ma Y Liu Z Deng L Du J Fan Z Ma T. FGF21 attenuates neuroinflammation following subarachnoid hemorrhage through promoting mitophagy and inhibiting the cGAS-STING pathway. J Transl Med. (2024) 22:436. doi: 10.1186/s12967-024-05239-y 38720350 PMC11077765

[166] Hernández-Ortega K Canul-Euan AA Solis-Paredes JM Borboa-Olivares H Reyes-Muñoz E Estrada-Gutierrez G. S100B actions on glial and neuronal cells in the developing brain: an overview. Front Neurosci. (2024) 18:1425525. doi: 10.3389/fnins.2024.1425525 39027325 PMC11256909

[167] Oris C Kahouadji S Bouvier D Sapin V. Blood biomarkers for the management of mild traumatic brain injury in clinical practice. Clin Chem. (2024) 70:1023–36. doi: 10.1093/clinchem/hvae049 38656380

[168] Anogianakis G Daios S Topouzis N Barmpagiannos K Kaiafa G Myrou A. Current trends in stroke biomarkers: the prognostic role of S100 calcium-binding protein B and glial fibrillary acidic protein. Life (Basel). (2024) 14:1247. doi: 10.3390/life14101247 39459548 PMC11508791

[169] Kleindienst A Meissner S Eyupoglu IY Parsch H Schmidt C Buchfelder M. Dynamics of S100B release into serum and cerebrospinal fluid following acute brain injury. Acta Neurochir Suppl. (2010) 106:247–50. doi: 10.1007/978-3-211-98811-4_46 19812958

[170] Michetti F D’Ambrosi N Toesca A Puglisi MA Serrano A Marchese E. The S100B story: from biomarker to active factor in neural injury. J Neurochem. (2019) 148:168–87. doi: 10.1111/jnc.14574 30144068

[171] Chong ZZ. S100B raises the alert in subarachnoid hemorrhage. Rev Neurosci. (2016) 27:745–59. doi: 10.1515/revneuro-2016-0021 27442365

[172] Sanchez-Peña P Pereira AR Sourour NA Biondi A Lejean L Colonne C. S100B as an additional prognostic marker in subarachnoid aneurysmal hemorrhage. Crit Care Med. (2008) 36:2267–73. doi: 10.1097/CCM.0b013e3181809750 18596638

[173] Jung CS Lange B Zimmermann M Seifert V. CSF and serum biomarkers focusing on cerebral vasospasm and ischemia after subarachnoid hemorrhage. Stroke Res Treat. (2013) 2013:560305. doi: 10.1155/2013/560305 23509668 PMC3590649

[174] Kaneda K Fujita M Yamashita S Kaneko T Kawamura Y Izumi T. Prognostic value of biochemical markers of brain damage and oxidative stress in post-surgical aneurysmal subarachnoid hemorrhage patients. Brain Res Bull. (2010) 81:173–7. doi: 10.1016/j.brainresbull.2009.10.020 19887101

[175] Ding S Wang C Wang W Yu H Chen B Liu L. Autocrine S100B in astrocytes promotes VEGF-dependent inflammation and oxidative stress and causes impaired neuroprotection. Cell Biol Toxicol. (2023) 39:1–25. doi: 10.1007/s10565-021-09674-1 34792689

[176] Villarreal A Seoane R González Torres A Rosciszewski G Angelo MF Rossi A. S100B protein activates a RAGE-dependent autocrine loop in astrocytes: implications for its role in the propagation of reactive gliosis. J Neurochem. (2014) 131:190–205. doi: 10.1111/jnc.12790 24923428

[177] Qian SQ He SR Li BB Qian J Zheng XD. Serum S100A12 and 30-day mortality after acute intracerebral hemorrhage. Clin Chim Acta. (2018) 477:1–6. doi: 10.1016/j.cca.2017.11.032 29196185

[178] Sreejit G Abdel-Latif A Athmanathan B Annabathula R Dhyani A Noothi SK. Neutrophil-derived S100A8/A9 amplify granulopoiesis after myocardial infarction. Circulation. (2020) 141:1080–94. doi: 10.1161/CIRCULATIONAHA.119.043833 PMC7122461 31941367

[179] Guo Q Zhao Y Li J Liu J Yang X Guo X. Induction of alarmin S100A8/A9 mediates activation of aberrant neutrophils in the pathogenesis of COVID-19. Cell Host Microbe. (2021) 29:222–235.e4. doi: 10.1016/j.chom.2020.12.016 33388094 PMC7762710

[180] Jin S Park CO Shin JU Noh JY Lee YS Lee NR. DAMP molecules S100A9 and S100A8 activated by IL-17A and house-dust mites are increased in atopic dermatitis. Exp Dermatol. (2014) 23:938–41. doi: 10.1111/exd.12563 25308296

[181] Tao Q Qiu X Li C Zhou J Gu L Zhang L. S100A8 regulates autophagy-dependent ferroptosis in microglia after experimental subarachnoid hemorrhage. Exp Neurol. (2022) 357:114171. doi: 10.1016/j.expneurol.2022.114171 35870523

[182] Wang G Hou G Tian Q Liu C Guo Y Wei H. Inhibition of S100A9 alleviates neurogenic pulmonary edema after subarachnoid hemorrhage. Biochem Pharmacol. (2023) 218:115905. doi: 10.1016/j.bcp.2023.115905 37949322

[183] Nakaoka H Tajima A Yoneyama T Hosomichi K Kasuya H Mizutani T. Gene expression profiling reveals distinct molecular signatures associated with the rupture of intracranial aneurysm. Stroke. (2014) 45:2239–45. doi: 10.1161/STROKEAHA.114.005851 24938844

[184] Wang G Huang K Tian Q Liu C Guo Y Wei H. S100A9 aggravates early brain injury after subarachnoid hemorrhage via inducing neuroinflammation and inflammasome activation. iScience. (2024) 27:109165. doi: 10.1016/j.bcp.2023.115905 38420589 PMC10901081

[185] Wanvimonsuk S Jaree P Kawai T Somboonwiwat K. Prx4 acts as DAMP in shrimp, enhancing bacterial resistance via the toll pathway and prophenoloxidase activation. iScience. (2022) 26:105793. doi: 10.1016/j.isci.2022.105793 36619979 PMC9813724

[186] Richard S Lapierre V Girerd N Bonnerot M Burkhard PR Lagerstedt L. Diagnostic performance of peroxiredoxin 1 to determine time-of-onset of acute cerebral infarction. Sci Rep. (2016) 6:38300. doi: 10.1038/srep38300 27924073 PMC5141372

[187] Leak RK Zhang L Luo Y Li P Zhao H Liu X. Peroxiredoxin 2 battles poly(ADP-ribose) polymerase 1- and p53-dependent prodeath pathways after ischemic injury. Stroke. (2013) 44:1124–34. doi: 10.1161/STROKEAHA.111.680157 PMC3891055 23429506

[188] Li H Wang Z Xie X Luo M Shen H Li X. Peroxiredoxin-3 plays a neuroprotective role in early brain injury after experimental subarachnoid hemorrhage in rats. Brain Res Bull. (2023) 193:95–105. doi: 10.1016/j.brainresbull.2022.12.010 36566946

[189] Xu N Jiang X Zhang W Shi Y Leak RK Keep RF. Endothelial peroxiredoxin-4 is indispensable for blood-brain barrier integrity and long-term functional recovery after ischemic stroke. Proc Natl Acad Sci U S A. (2024) 121:e2400272121. doi: 10.1073/pnas.2400272121 38437534 PMC10945775

[190] Kunze A Zierath D Tanzi P Cain K Becker K. Peroxiredoxin 5 (PRX5) is correlated inversely to systemic markers of inflammation in acute stroke. Stroke. (2014) 45:608–10. doi: 10.1161/STROKEAHA.113.003813 PMC3946812 24385276

[191] Liu DL Zhao LX Zhang S Du JR. Peroxiredoxin 1-mediated activation of TLR4/NF-κB pathway contributes to neuroinflammatory injury in intracerebral hemorrhage. Int Immunopharmacol. (2016) 41:82–9. doi: 10.1016/j.intimp.2016.10.025 27821296

[192] Lu Y Zhang XS Zhang ZH Zhou XM Gao YY Liu GJ. Peroxiredoxin 2 activates microglia by interacting with Toll-like receptor 4 after subarachnoid hemorrhage. J Neuroinflammation. (2018) 15:87. doi: 10.1186/s12974-018-1118-4 29554978 PMC5859544

[193] Du Y Wang J Zhang J Li N Li G Liu X. Intracerebral hemorrhage-induced brain injury in mice: The role of peroxiredoxin 2-Toll-like receptor 4 inflammatory axis. CNS Neurosci Ther. (2024) 30:e14681. doi: 10.1111/cns.14681 38516845 PMC10958402

[194] Zhang J Novakovic N Hua Y Keep RF Xi G. Role of lipocalin-2 in extracellular peroxiredoxin 2-induced brain swelling, inflammation and neuronal death. Exp Neurol. (2021) 335:113521. doi: 10.1016/j.expneurol.2020.113521 33129840 PMC7750274

[195] Liu X Hong E Xie J Li J Ding B Chen Y. Txnrd2 Attenuates Early Brain Injury by Inhibition of Oxidative Stress and Endoplasmic Reticulum Stress via Trx2/Prx3 Pathway after Intracerebral Hemorrhage in Rats. Neuroscience. (2024) 545:158–70. doi: 10.1016/j.neuroscience.2024.03.019 38513765

[196] Alatas ÖD Gürger M Ateşçelik M Yildiz M Demir CF Ekingen E. Neuron-specific enolase, S100 calcium-binding protein B, and heat shock protein 70 levels in patients with intracranial hemorrhage. Med (Baltimore). (2015) 94:1. Ekingen, Evren [Added]]. *Medicine (Baltimore).* 2015;94(45):e2007. doi: 10.1097/MD.0000000000002007 PMC4912289 26559295

[197] Li M Yao M Shao K Shen X Ge Z Li Y. Serum cold-inducible RNA-binding protein (CIRP) levels as a prognostic indicator in patients with acute ischemic stroke. Front Neurol. (2023) 14:1290135. doi: 10.3389/fneur.2023.1290135 37521290 PMC10381024

[198] Chen Z Hu Q Huo Y Zhang R Fu Q Qin X. Serum interleukin-33 is a novel predictive biomarker of hemorrhage transformation and outcome in acute ischemic stroke. J Stroke Cerebrovasc Dis. (2021) 30:105506. doi: 10.1016/j.jstrokecerebrovasdis.2020.105506 33307292

[199] Shao A Zhou Y Yao Y Zhang W Zhang J Deng Y. The role and therapeutic potential of heat shock proteins in haemorrhagic stroke. J Cell Mol Med. (2019) 23:5846–58. doi: 10.1111/jcmm.14479 PMC6714234 31273911

[200] Zhou K Cui S Duan W Zhang J Huang J Wang L. Cold-inducible RNA-binding protein contributes to intracerebral hemorrhage-induced brain injury via TLR4 signaling. Brain Behav. (2020) 10:e01618. doi: 10.1002/brb3.1618 32285591 PMC7303400

[201] Gao Y Ma L Luo CL Wang T Zhang MY Shen X. IL-33 exerts neuroprotective effect in mice intracerebral hemorrhage model through suppressing inflammation/apoptotic/autophagic pathway. Mol Neurobiol. (2017) 54:3879–92. doi: 10.1007/s12035-016-9947-6 27405469

[202] Yang Y Liu H Zhang H Ye Q Wang J Yang B. ST2/IL-33-dependent microglial response limits acute ischemic brain injury. J Neurosci. (2017) 37:4692–704. doi: 10.1523/JNEUROSCI.3233-16.2017 PMC5426564 28389473

[203] Jiao M Li X Chen L Wang X Yuan B Liu T. Neuroprotective effect of astrocyte-derived IL-33 in neonatal hypoxic-ischemic brain injury. J Neuroinflammation. (2020) 17:251. doi: 10.1186/s12974-020-01932-z 32859229 PMC7455908

[204] Liu YX Zhao M Yu Y Liu JP Liu WJ Yao RQ. Extracellular cold-inducible RNA-binding protein mediated neuroinflammation and neuronal apoptosis after traumatic brain injury. Burns Trauma. (2024) 12:tkae004. doi: 10.1093/burnst/tkae004 38817684 PMC11136617

[205] Richards CM McRae SA Ranger AL Klegeris A. Extracellular histones as damage-associated molecular patterns in neuroinflammatory responses. Rev Neurosci. (2022) 34:533–58. doi: 10.1515/revneuro-2022-0091 36368030

[206] Wang J. Preclinical and clinical research on inflammation after intracerebral hemorrhage. Prog Neurobiol. (2010) 92:463–77. doi: 10.1016/j.pneurobio.2010.08.001 PMC2991407 20713126

[207] Neves JD Aristimunha D Vizuete AF Nicola F Vanzella C Petenuzzo L. Glial-associated changes in the cerebral cortex after collagenase-induced intracerebral hemorrhage in the rat striatum. Brain Res Bull. (2017) 134:55–62. doi: 10.1016/j.brainresbull.2017.07.002 28705495

[208] Wu Y Eisel ULM. Microglia-astrocyte communication in alzheimer’s disease. J Alzheimers Dis. (2023) 95:785–803. doi: 10.3233/JAD-230199 37638434 PMC10578295

[209] Akeret K Buzzi RM Thomson BR Schwendinger N Klohs J Schulthess-Lutz N. MyD88-TLR4-dependent choroid plexus activation precedes perilesional inflammation and secondary brain edema in a mouse model of intracerebral hemorrhage. J Neuroinflammation. (2022) 19:290. doi: 10.1186/s12974-022-02641-5 36482445 PMC9730653

[210] Zheng J Wu H Wang X Zhang G Lu J Xu W. Temporal dynamics of microglia-astrocyte interaction in neuroprotective glial scar formation after intracerebral hemorrhage. J Pharm Anal. (2023) 13:862–79. doi: 10.1016/j.jpha.2023.02.007 PMC10499589 37719195

[211] Gu L Chen H Sun M Chen Y Shi Q Chang J. Unraveling dynamic immunological landscapes in intracerebral hemorrhage: insights from single-cell and spatial transcriptomic profiling. MedComm (2020). (2024) 5:e635. doi: 10.1002/mco2.635 38988493 PMC11233862

[212] Lan X Han X Li Q Yang QW Wang J. Modulators of microglial activation and polarization after intracerebral haemorrhage. Nat Rev Neurol. (2017) 13:420–33. doi: 10.1038/nrneurol.2017.69 PMC5575938 28524175

[213] Wu Y Xu Y Sun J Dai K Wang Z Zhang J. Inhibiting RIPK1-driven neuroinflammation and neuronal apoptosis mitigates brain injury following experimental subarachnoid hemorrhage. Exp Neurol. (2024) 374:114705. doi: 10.1016/j.expneurol.2024.114705 38290652

[214] Greenhalgh AD Brough D Robinson EM Girard S Rothwell NJ Allan SM. Interleukin-1 receptor antagonist is beneficial after subarachnoid haemorrhage in rat by blocking haem-driven inflammatory pathology. Dis Model Mech. (2012) 5:823–33. doi: 10.1242/dmm.008557 PMC3484865 22679224

[215] Liddelow SA Guttenplan KA Clarke LE Bennett FC Bohlen CJ Schirmer L. Neurotoxic reactive astrocytes are induced by activated microglia. Nature. (2017) 541:481–7. doi: 10.1038/nature21029 PMC5404890 28099414

[216] Pinteaux E Rothwell NJ Boutin H. Neuroprotective actions of endogenous interleukin-1 receptor antagonist (IL-1ra) are mediated by glia. Glia. (2006) 53:551–6. doi: 10.1002/glia.20308 16374779

[217] Deng S Chen X Lei Q Lu W. AQP2 promotes astrocyte activation by modulating the TLR4/NFκB-p65 pathway following intracerebral hemorrhage. Front Immunol. (2022) 13 :847360. doi: 10.3389/fimmu.2022.847360 35386692 PMC8978957

[218] Long J Sun Y Liu S Yang S Chen C Zhang Z. Targeting pyroptosis as a preventive and therapeutic approach for stroke. Cell Death Discov. (2023) 9:155. doi: 10.1038/s41420-023-01440-y 37165005 PMC10172388

[219] Xu B Li H Zheng H Gao Z Miao Z Xu X. Interleukin-18 interacts with NKCC1 to mediate brain injury after intracerebral hemorrhage. Brain Behavior Immunity-Health. (2024) 42:100890. doi: 10.1016/j.bbih.2024.100890 39507306 PMC11538613

[220] Lee SC Liu W Dickson DW Brosnan CF Berman JW. Cytokine production by human fetal microglia and astrocytes. Differential induction by lipopolysaccharide and IL-1 beta. J Immunol. (1993) 150:2659–67. doi: 10.4049/jimmunol.150.7.2659 8454848

[221] Jedrzejowska-Szypułka H Straszak G Larysz-Brysz M Karpe J Marcol W Olakowska E. Interleukin-1beta plays a role in the activation of peripheral leukocytes after blood-brain barrier rupture in the course of subarachnoid hemorrhage. Curr Neurovasc Res. (2010) 7:39–48. doi: 10.2174/156720210790820226 20158463

[222] Sheng F Li M Yu JM Yang SY Zou L Yang GJ. IL-33/ST2 axis in diverse diseases: regulatory mechanisms and therapeutic potential. Front Immunol. (2025) 16:1533335. doi: 10.3389/fimmu.2025.1533335 39925809 PMC11802536

[223] He D Xu H Zhang H Tang R Lan Y Xing R. Disruption of the IL-33-ST2-AKT signaling axis impairs neurodevelopment by inhibiting microglial metabolic adaptation and phagocytic function. Immunity. (2022) 55:159–173.e9. doi: 10.1016/j.immuni.2021.12.001 34982959 PMC9074730

[224] Huang LT Li H Sun Q Liu M Li WD Li S. IL-33 expression in the cerebral cortex following experimental subarachnoid hemorrhage in rats. Cell Mol Neurobiol. (2015) 35:493–501. doi: 10.1007/s10571-014-0143-9 25417195 PMC11486190

[225] Chen Z Xu N Dai X Zhao C Wu X Shankar S. Interleukin-33 reduces neuronal damage and white matter injury via selective microglia M2 polarization after intracerebral hemorrhage in rats. Brain Res Bull. (2019) 150:127–35. doi: 10.1016/j.brainresbull.2019.05.016 31129170

[226] Pierre WC Londono I Quiniou C Chemtob S Lodygensky GA. Modulatory effect of IL-1 inhibition following lipopolysaccharide-induced neuroinflammation in neonatal microglia and astrocytes. Int J Dev Neurosci. (2022) 82:243–60. doi: 10.1002/jdn.10179 35315121

[227] Shi SX Li YJ Shi K Wood K Ducruet AF Liu Q. IL (Interleukin)-15 bridges astrocyte-microglia crosstalk and exacerbates brain injury following intracerebral hemorrhage. Stroke. (2020) 51:967–74. 10.1161/STROKEAHA.119.028638 32019481

[228] Simon M Grote A. Interleukin 6 and aneurysmal subarachnoid hemorrhage. A Narrative Review. Int J Mol Sci. (2021) 22:4133. doi: 10.3390/ijms22084133 33923626 PMC8073154

[229] Gowrisankar YV Clark MA. Angiotensin II induces interleukin-6 expression in astrocytes: role of reactive oxygen species and NF-kappaB. Mol Cell Endocrinol. (2016) 437:130–41. doi: 10.1016/j.mce.2016.08.013 27539920

[230] Lucke-Wold B Dodd W Motwani K Hosaka K Laurent D Martinez M. Investigation and modulation of interleukin-6 following subarachnoid hemorrhage: targeting inflammatory activation for cerebral vasospasm. J Neuroinflammation. (2022) 19:228. doi: 10.1186/s12974-022-02592-x 36114540 PMC9479230

[231] Xie L Zhang N Zhang Q Li C Sandhu AF Iii GW. Inflammatory factors and amyloid β-induced microglial polarization promote inflammatory crosstalk with astrocytes. Aging (Albany NY). (2020) 12:22538–49. doi: 10.18632/aging.103663 PMC7746366 33196457

[232] Siglienti I Chan A Kleinschnitz C Jander S Toyka KV Gold R. Downregulation of transforming growth factor-beta2 facilitates inflammation in the central nervous system by reciprocal astrocyte/microglia interactions. J Neuropathol Exp Neurol. (2007) 66:47–56. doi: 10.1097/nen.0b013e31802d47b4 17204936

[233] Xu J Chen Z Yu F Liu H Ma C Xie D. IL-4/STAT6 signaling facilitates innate hematoma resolution and neurological recovery after hemorrhagic stroke in mice. Proc Natl Acad Sci U S A. (2020) 117:32679–90. doi: 10.1073/pnas.201849711 PMC7768771 33293423

[234] Norden DM Fenn AM Dugan A Godbout JP. TGFβ produced by IL-10 redirected astrocytes attenuates microglial activation. Glia. (2014) 62:881–95. 10.1002/glia.22647 PMC4061706 24616125

[235] Taylor RA Chang CF Goods BA Hammond MD Mac Grory B Ai Y. TGF-β1 modulates microglial phenotype and promotes recovery after intracerebral hemorrhage. J Clin Invest. (2017) 127:280–92. 10.1172/JCI88647 PMC5199690 27893460

[236] Cekanaviciute E Fathali N Doyle KP Williams AM Han J Buckwalter MS. Astrocytic transforming growth factor-beta signaling reduces subacute neuroinflammation after stroke in mice. Glia. (2014) 62:1227–40. doi: 10.1002/glia.22675 PMC4061255 24733756

[237] McAlpine CS Park J Griciuc A Kim E Choi SH Iwamoto Y. Astrocytic interleukin-3 programs microglia and limits Alzheimer’s disease. Nature. (2021) 595:701–6. doi: 10.1038/s41586-021-03734-6 PMC8934148 34262178

[238] Miyoshi K Obata K Kondo T Okamura H Noguchi K. Interleukin-18-mediated microglia/astrocyte interaction in the spinal cord enhances neuropathic pain processing after nerve injury. J Neurosci. (2008) 28:12775–87. doi: 10.1523/JNEUROSCI.3512-08.2008 PMC6671812 19036970

[239] Kyrkanides S Olschowka JA Williams JP Hansen JT O’Banion MK. TNF alpha and IL-1beta mediate intercellular adhesion molecule-1 induction via microglia-astrocyte interaction in CNS radiation injury. J Neuroimmunol. (1999) 95:95–106. doi: 10.1016/s0165-5728(98)00270-7 10229119

[240] He M Dong H Huang Y Lu S Zhang S Qian Y. Astrocyte-derived CCL2 is associated with M1 activation and recruitment of cultured microglial cells. Cell Physiol Biochem. (2016) 38:859–70. doi: 10.1159/000443040 26910882

[241] Xu J Dong H Qian Q Zhang X Wang Y Jin W. Astrocyte-derived CCL2 participates in surgery-induced cognitive dysfunction and neuroinflammation via evoking microglia activation. Behav Brain Res. (2017) 332:145–53. doi: 10.1016/j.bbr.2017.05.066 28587818

[242] Yao Y Tsirka SE. The CCL2-CCR2 system affects the progression and clearance of intracerebral hemorrhage. Glia. (2012) 60:908–18. doi: 10.1002/glia.22323 PMC3325358 22419223

[243] Xu D Gao Q Wang F Peng Q Wang G Wei Q. Sphingosine-1-phosphate receptor 3 is implicated in BBB injury via the CCL2-CCR2 axis following acute intracerebral hemorrhage. CNS Neurosci Ther. (2021) 27:674–86. doi: 10.1111/cns.13626 PMC8111497 33645008

[244] Liang P Zhang X Zhang Y Wu Y Song Y Wang X. Neurotoxic A1 astrocytes promote neuronal ferroptosis via CXCL10/CXCR3 axis in epilepsy. Free Radic Biol Med. (2023) 195:329–42. doi: 10.1016/j.freeradbiomed.2023.01.002 36610561

[245] Clarner T Janssen K Nellessen L Stangel M Skripuletz T Krauspe B. CXCL10 triggers early microglial activation in the cuprizone model. J Immunol. (2015) 194:3400–13. doi: 10.4049/jimmunol.1401459 25725102

[246] Kuboyama K Harada H Tozaki-Saitoh H Tsuda M Ushijima K Inoue K. Astrocytic P2Y(1) receptor is involved in the regulation of cytokine/chemokine transcription and cerebral damage in a rat model of cerebral ischemia. J Cereb Blood Flow Metab. (2011) 31:1930–41. doi: 10.1038/jcbfm.2011.49 PMC3185880 21487414

[247] Cheng F Wang C Yan B Yin Z Liu Y Zhang L. CSF1R blockade slows progression of cerebral hemorrhage by reducing microglial proliferation and increasing infiltration of CD8 + CD122+ T cells into the brain. Int Immunopharmacol. (2024) 133:112071. doi: 10.1016/j.intimp.2024.112071 38636374

[248] Ho MH Tsai YJ Chen CY Yang A Burnouf T Wang Y. CCL5 is essential for axonogenesis and neuronal restoration after brain injury. J BioMed Sci. (2024) 31:91. doi: 10.1186/s12929-024-01083-w 39285280 PMC11406852

[249] Zhou S Liu C Wang J Ye J Lian Q Gan L. CCL5 mediated astrocyte-T cell interaction disrupts blood-brain barrier in mice after hemorrhagic stroke. J Cereb Blood Flow Metab. (2024) 44:367–83. doi: 10.1177/0271678X231214838 PMC10870968 37974301

[250] Skuljec J Sun H Pul R Bénardais K Ragancokova D Moharregh-Khiabani D. CCL5 induces a pro-inflammatory profile in microglia. vitro. Cell Immunol. (2011) 270:164–71. doi: 10.1016/j.cellimm.2011.05.001 21620385

[251] Zhang Z Li Y Jiang S Shi FD Shi K Jin WN. Targeting CCL5 signaling attenuates neuroinflammation after seizure. CNS Neurosci Ther. (2023) 29:317–30. doi: 10.1111/cns.14006 PMC9804050 36440924

[252] Yan J Xu W Lenahan C Huang L Wen J Li G. CCR5 activation promotes NLRP1-dependent neuronal pyroptosis via CCR5/PKA/CREB pathway after intracerebral hemorrhage. Stroke. (2024) 55:e232. doi: 10.1161/STROKEAHA.120.033285 34719258 PMC8607924

[253] Lin J Xu Y Guo P Chen YJ Zhou J Xia M. CCL5/CCR5-mediated peripheral inflammation exacerbates blood–brain barrier disruption after intracerebral hemorrhage in mice. J Transl Med. (2023) 21:196. doi: 10.1186/s12967-023-04044-3 36918921 PMC10015963

[254] Wei Y Chen T Bosco DB Xie M Zheng J Dheer A. The complement C3-C3aR pathway mediates microglia-astrocyte interaction following status epilepticus. Glia. (2021) 69:1155–69. doi: 10.1002/glia.23955 PMC7936954 33314324

[255] Lian H Yang L Cole A Sun L Chiang AC Fowler SW. NFκB-activated astroglial release of complement C3 compromises neuronal morphology and function associated with Alzheimer’s disease. Neuron. (2015) 85:101–15. doi: 10.1016/j.neuron.2014.11.018 PMC4289109 25533482

[256] Gnanaguru G Tabor SJ Bonilla GM Sadreyev R Yuda K Köhl J. Microglia refine developing retinal astrocytic and vascular networks through the complement C3/C3aR axis. Development. (2023) 150:dev201047. doi: 10.1242/dev.201047 36762625 PMC10110418

[257] Zhang W Ding L Chen H Zhang M Ma R Zheng S. Cntnap4 partial deficiency exacerbates α-synuclein pathology through astrocyte-microglia C3-C3aR pathway. Cell Death Dis. (2023) 14:285. doi: 10.1038/s41419-023-05807-y 37087484 PMC10122675

[258] Tang J Jila S Luo T Zhang B Miao H Feng H. C3/C3aR inhibition alleviates GMH-IVH-induced hydrocephalus by preventing microglia-astrocyte interactions in neonatal rats. Neuropharmacology. (2022) 205:108927. doi: 10.1016/j.neuropharm.2021.108927 34921829

[259] Mou W Ma L Zhu A Cui H Huang Y. Astrocyte-microglia interaction through C3/C3aR pathway modulates neuropathic pain in rats model of chronic constriction injury. Mol Pain. (2022) 18:17448069221140532. doi: 10.1177/17448069221140532 36341694 PMC9669679

[260] Asano S Hayashi Y Iwata K Okada-Ogawa A Hitomi S Shibuta I. Microglia-astrocyte communication via C1q contributes to orofacial neuropathic pain associated with infraorbital nerve injury. Int J Mol Sci. (2020) 21:6834. doi: 10.3390/ijms21186834 32957694 PMC7560139

[261] Litvinchuk A Wan YW Swartzlander DB Chen F Cole A Propson NE. Complement C3aR inactivation attenuates tau pathology and reverses an immune network deregulated in tauopathy models and alzheimer’s disease. Neuron. (2018) 100:1337–1353.e5. doi: 10.1016/j.neuron.2018.10.031 30415998 PMC6309202

[262] Li Y Tao C An N Liu H Liu Z Zhang H. Revisiting the role of the complement system in intracerebral hemorrhage and therapeutic prospects. Int Immunopharmacol. (2023) 123:110744. doi: 10.1016/j.intimp.2023.110744 37552908

[263] Wang Z Wu X Yan T Liu M Yu W Du Q. Elevated plasma complement C1q levels contribute to a poor prognosis after acute primary intracerebral hemorrhage: A prospective cohort study. Front Immunol. (2022) 13:920754. doi: 10.3389/fimmu.2022.920754 35812425 PMC9259799

[264] Kasuya H Shimizu T. Activated complement components C3a and C4a in cerebrospinal fluid and plasma following subarachnoid hemorrhage. J Neurosurg. (1989) 71:741–6. doi: 10.3171/jns.1989.71.5.0741 2809729

[265] Wang M Xia F Wan S Hua Y Keep RF Xi G. Role of complement component 3 in early erythrolysis in the hematoma after experimental intracerebral hemorrhage. Stroke. (2021) 52:2649–60. doi: 10.1161/STROKEAHA.121.034372 PMC8316397 34176310

[266] Ming Y Zhao P Zhang H Zhang Z Huang Z Zhang L. Complement molecule C3a exacerbates early brain injury after subarachnoid hemorrhage by inducing neuroinflammation through the C3aR-ERK-P2X7-NLRP3 inflammasome signaling axis. Inflammation. (2024). doi: 10.1007/s10753-024-02155-7 39528767

[267] Rynkowski MA Kim GH Garrett MC Zacharia BE Otten ML Sosunov SA. C3a receptor antagonist attenuates brain injury after intracerebral hemorrhage. J Cereb Blood Flow Metab. (2009) 29:98–107. doi: 10.1038/jcbfm.2008.95 18728680 PMC3731073

[268] Yang L Wu J Zhang F Zhang L Zhang X Zhou J. Microglia aggravate white matter injury via C3/C3aR pathway after experimental subarachnoid hemorrhage. Exp Neurol. (2024) 379:114853. doi: 10.1016/j.expneurol.2024.114853 38866102

[269] Nowrangi DS McBride D Manaenko A Dixon B Tang J Zhang JH. rhIGF-1 reduces the permeability of the blood-brain barrier following intracerebral hemorrhage in mice. Exp Neurol. (2019) 312:72–81. doi: 10.1016/j.expneurol.2018.11.009 30503192

[270] Akturk UD Tuncer C Bozkurt H Sahin OS Bulut H Arikok A. Blocking VEGF by bevacizumab attenuates VEGF-induced vasospasm after experimental subarachnoid hemorrhage in rabbits. World Neurosurg. (2020) 139:e136–43. doi: 10.1016/j.wneu.2020.03.151 32251821

[271] Goshi N Morgan RK Lein PJ Seker E. A primary neural cell culture model to study neuron, astrocyte, and microglia interactions in neuroinflammation. J Neuroinflamm. (2022) 19:49. doi: 10.1186/s12974-022-02391-4. *J Neuroinflammation.* 2020;17(1):155. PMC7216677 32393376

[272] Rothhammer V Borucki DM Tjon EC Takenaka MC Chao CC Ardura-Fabregat A. Microglial control of astrocytes in response to microbial metabolites. Nature. (2018) 557:724–8. doi: 10.1038/s41586-018-0119-x PMC6422159 29769726

[273] Wang S Guo Y Cao RQ. VEGFD/VEGFR3 signaling contributes to the dysfunction of the astrocyte IL-3/microglia IL-3Rα cross-talk and drives neuroinflammation in mouse ischemic stroke. Acta Pharmacol Sin. (2024) 46(2):292–307. doi: Zhu YM, Qiao SG, Du HP, 10.1038/s41401-024-01405-6 PMC11747567 39478160

[274] Wang QS Ding HG Chen SL Liu XQ Deng YY Jiang WQ. Hypertonic saline mediates the NLRP3/IL-1β signaling axis in microglia to alleviate ischemic blood-brain barrier permeability by downregulating astrocyte-derived VEGF in rats. CNS Neurosci Ther. (2020) 26:1045–57. doi: 10.1111/cns.13427 PMC7539845 32529750

[275] Upadhya D Shetty AK. Extracellular vesicles as therapeutics for brain injury and disease. Curr Pharm Des. (2019) 25:3500–5. doi: 10.2174/1381612825666191014164950 31612823

[276] Sheng B Lai N Tao T Chen X Gao S Zhu Q. Diagnosis potential of subarachnoid hemorrhage using miRNA signatures isolated from plasma-derived extracellular vesicles. Front Pharmacol. (2023) 14:1090389. doi: 10.3389/fphar.2023.1090389 36860299 PMC9968748

[277] Casado-Fernández L Laso-García F Piniella D Gómez-de Frutos MC Otero-Ortega L Bravo SB. The proteomic signature of circulating extracellular vesicles following intracerebral hemorrhage: Novel insights into mechanisms underlying recovery. Neurobiol Dis. (2024) 201:106665. doi: 10.1016/j.nbd.2024.106665 39277144

[278] Laso-García F Piniella D Gómez-de Frutos MC Casado-Fernández L Pérez-Mato M Alonso-López E. Protein content of blood-derived extracellular vesicles: An approach to the pathophysiology of cerebral hemorrhage. Front Cell Neurosci. (2023) 16:1058546. doi: 10.3389/fncel.2022.1058546 36776230 PMC9912619

[279] Laso-García F Casado-Fernández L Piniella D Gómez-de Frutos MC Arizaga-Echebarria JK Pérez-Mato M. Circulating extracellular vesicles promote recovery in a preclinical model of intracerebral hemorrhage. Mol Ther Nucleic Acids. (2023) 32:247–62. doi: 10.1016/j.omtn.2023.03.006 PMC10113711 37090418

[280] Zhu H Wang N Chang Y Zhang Y Jiang S Ren X. Extracellular vesicles bearing serum amyloid A1 exacerbate neuroinflammation after intracerebral haemorrhage. Stroke Vasc Neurol. (2024) 2:svn-2024-003525. doi: 10.1136/svn-2024-003525 PMC12230225 39357895

[281] Jiang S Li X Li Y Chang Z Yuan M Zhang Y. APOE from patient-derived astrocytic extracellular vesicles alleviates neuromyelitis optica spectrum disorder in a mouse model. Sci Transl Med. (2024) 16:eadg5116. doi: 10.1126/scitranslmed.adg5116 38416841

[282] Long X Yao X Jiang Q Yang Y He X Tian W. Astrocyte-derived exosomes enriched with miR-873a-5p inhibit neuroinflammation via microglia phenotype modulation after traumatic brain injury. J Neuroinflammation. (2020) 17:89. doi: 10.1186/s12974-020-01761-0 32192523 PMC7082961

[283] Han J Cho HJ Park D Han S. DICAM in the extracellular vesicles from astrocytes attenuates microglia activation and neuroinflammation. Cells. (2022) 11:2977. doi: 10.3390/cells11192977 36230938 PMC9562652

[284] Wang Y Li H Sun H Xu C Sun H Wei W. A2 reactive astrocyte-derived exosomes alleviate cerebral ischemia-reperfusion injury by delivering miR-628. J Cell Mol Med. (2024) 28:e70004. doi: 10.1111/jcmm.70004 39159174 PMC11332600

[285] Fei X Dou YN Wang L Wu X Huan Y Wu S. Homer1 promotes the conversion of A1 astrocytes to A2 astrocytes and improves the recovery of transgenic mice after intracerebral hemorrhage. J Neuroinflammation. (2022) 19:67. doi: 10.1186/s12974-022-02428-8 35287697 PMC8922810

[286] Zhao G Jing J. HOMER1A restores sevoflurane-induced cognitive dysfunction by regulating microglia’s activation through activating the AMPK/TXNIP axis. Signa Vitae. (2023) 19:4. doi: 10.22514/sv.2023.065

[287] Fei X Wang L Dou YN Fei F Zhang Y Lv W. Extracellular vesicle encapsulated Homer1a as novel nanotherapeutics against intracerebral hemorrhage in a mouse model. J Neuroinflammation. (2024) 21:85. 38582897 10.1186/s12974-024-03088-6 PMC10999083

[288] Söderholm M Nordin Fredrikson G Nilsson J Engström G. High serum level of matrix metalloproteinase-7 is associated with increased risk of spontaneous subarachnoid hemorrhage. Stroke. (2018) 49:1626–31. doi: 10.1161/STROKEAHA.118.020660 29880550

[289] Egashira Y Zhao H Hua Y Keep RF Xi G. White matter injury after subarachnoid hemorrhage: role of blood-brain barrier disruption and matrix metalloproteinase-9. Stroke. (2015) 46:2909–15. doi: 10.1161/STROKEAHA.115.010351 PMC4589516 26374478

[290] Xue M Hollenberg MD Yong VW. Combination of thrombin and matrix metalloproteinase-9 exacerbates neurotoxicity in cell culture and intracerebral hemorrhage in mice. J Neurosci. (2006) 26:10281–91. doi: 10.1523/JNEUROSCI.2806-06.2006 PMC6674619 17021183

[291] Min H Hong J Cho IH Jang YH Lee H Kim D. TLR2-induced astrocyte MMP9 activation compromises the blood brain barrier and exacerbates intracerebral hemorrhage in animal models. Mol Brain. (2015) 8:23. 25879213 10.1186/s13041-015-0116-z PMC4397689

[292] Liu Y Bai Q Yong VW Xue M. EMMPRIN promotes the expression of MMP-9 and exacerbates neurological dysfunction in a mouse model of intracerebral hemorrhage. Neurochem Res. (2022) 47:2383–95. doi: 10.1007/s11064-022-03630-z 35608790

[293] Zhang Y Liu Y Zhang X Yong VW Xue M. Omarigliptin protects the integrity of the blood-brain barrier after intracerebral hemorrhage in mice. J Inflammation Res. (2023) 16:2535–48. doi: 10.2147/JIR.S411017 PMC10278948 37342770

[294] Suzuki H Hasegawa Y Kanamaru K Zhang JH. Mechanisms of osteopontin-induced stabilization of blood-brain barrier disruption after subarachnoid hemorrhage in rats. Stroke. (2010) 41:1783–90. 10.1161/STROKEAHA.110.586537 PMC2923856 20616319

[295] Wu B Ma Q Suzuki H Chen C Liu W Tang J. Recombinant osteopontin attenuates brain injury after intracerebral hemorrhage in mice. Neurocritical Care. (2011) 14:109–17. doi: 10.1007/s12028-010-9372-z 20440599

[296] Sun C Rahman MSU Enkhjargal B Peng J Zhou K Xie Z. Osteopontin modulates microglial activation states and attenuates inflammatory responses after subarachnoid hemorrhage in rats. Exp Neurol. (2024) 371:114585. doi: 10.1016/j.expneurol.2023.114585 37884185

[297] Gong L Manaenko A Fan R Huang L Enkhjargal B McBride D. Osteopontin attenuates inflammation via JAK2/STAT1 pathway in hyperglycemic rats after intracerebral hemorrhage. Neuropharmacology. (2018) 138:160–9. doi: 10.1016/j.neuropharm.2018.06.009 PMC6487497 29885817

[298] Gliem M Krammes K Liaw L van Rooijen N Hartung HP Jander S. Macrophage-derived osteopontin induces reactive astrocyte polarization and promotes re-establishment of the blood brain barrier after ischemic stroke. Glia. (2015) 63:2198–207. doi: 10.1002/glia.22885 26148976

[299] Xing C Wang X Cheng C Montaner J Mandeville E Leung W. Neuronal production of lipocalin-2 as a help-me signal for glial activation. Stroke. (2014) 45:2085–92. doi: 10.1161/STROKEAHA.114.005733 PMC4122238 24916903

[300] Ranjbar Taklimie F Gasterich N Scheld M Weiskirchen R Beyer C Clarner T. Hypoxia induces astrocyte-derived lipocalin-2 in ischemic stroke. Int J Mol Sci. (2019) 20:1271. doi: 10.3390/ijms20061271 30871254 PMC6471434

[301] Fei X Dou Y Yang Y Zheng B Luo P Dai S. Lipocalin-2 inhibition alleviates neural injury by microglia ferroptosis suppression after experimental intracerebral hemorrhage in mice via enhancing ferritin light chain expression. Biochim Biophys Acta Mol Basis Dis. (2024) 1870:167435. doi: 10.1016/j.bbadis.2024.167435 39067535

[302] Egashira Y Hua Y Keep RF Iwama T Xi G. Lipocalin 2 and blood-brain barrier disruption in white matter after experimental subarachnoid hemorrhage. Acta Neurochir Suppl. (2016) 121:131–4. doi: 10.1007/978-3-319-18497-5_23 26463936

[303] Gu L Chen H Geng R Sun M Shi Q Chen Y. Single-cell and spatial transcriptomics reveals ferroptosis as the most enriched programmed cell death process in hemorrhage stroke-induced oligodendrocyte-mediated white matter injury. Int J Biol Sci. (2024) 20:3842–62. doi: 10.7150/ijbs.96262 PMC11302879 39113700

[304] Bonvento G Bolaños JP. Astrocyte-neuron metabolic cooperation shapes brain activity. Cell Metab. (2021) 33:1546–64. doi: 10.1016/j.cmet.2021.07.006 34348099

[305] Czapski GA Strosznajder JB. Glutamate and GABA in microglia-neuron cross-talk in alzheimer’s disease. Int J Mol Sci. (2021) 22:11677. doi: 10.3390/ijms222111677 34769106 PMC8584169

[306] SChadt F Israel I Beez A Alushi K Weiland J Ernestus RI. Analysis of cerebral glucose metabolism following experimental subarachnoid hemorrhage over 7 days. Sci Rep. (2023) 13:427. doi: 10.1038/s41598-022-26183-1 36624132 PMC9829694

[307] Sarrafzadeh A Haux D Sakowitz O Benndorf G Herzog H Kuechler I. Acute focal neurological deficits in aneurysmal subarachnoid hemorrhage: relation of clinical course, CT findings, and metabolite abnormalities monitored with bedside microdialysis. Stroke. (2003) 34:1382–8. doi: 10.1161/01.STR.0000074036.97859.02 12750537

[308] Lilla N Füllgraf H Stetter C Köhler S Ernestus RI Westermaier T. First description of reduced pyruvate dehydrogenase enzyme activity following subarachnoid hemorrhage (SAH). Front Neurosci. (2017) 11:37. doi: 10.3389/fnins.2017.00037 28261039 PMC5306203

[309] Tholance Y Aboudhiaf S Balança B Barcelos GK Grousson S Carrillon R. Early brain metabolic disturbances associated with delayed cerebral ischemia in patients with severe subarachnoid hemorrhage. J Cereb Blood Flow Metab. (2023) 43:1967–82. doi: 10.1177/0271678X231193661 PMC10676142 37572080

[310] Ardanaz CG de la Cruz A Minhas PS Hernández-Martín N Pozo MÁ Valdecantos MP. Astrocytic GLUT1 reduction paradoxically improves central and peripheral glucose homeostasis. Sci Adv. (2024) 0:eadp1115. doi: 10.1126/sciadv.adp1115 PMC11488540 39423276

[311] Thieren L Zanker HS Droux J Dalvi U Wyss MT Waag R. Astrocytic GLUT1 deletion in adult mice enhances glucose metabolism and resilience to stroke. Nat Commun. (2025) 16:4190. doi: 10.1038/s41467-025-59400-2 40328784 PMC12056070

[312] Wang L Pavlou S Du X Bhuckory M Xu H Chen M. Glucose transporter 1 critically controls microglial activation through facilitating glycolysis. Mol Neurodegener. (2019) 14:2. doi: 10.1186/s13024-019-0305-9 30634998 PMC6329071

[313] Li Y Zhou H He X Jin L Zhu Y Hu L. Impaired microglial glycolysis promotes inflammatory responses after intracerebral haemorrhage via HK2-dependent mitochondrial dysfunction. J Adv Res. (2024) 73:575–91. doi: 10.1016/j.jare.2024.08.016 PMC12225926 39142439

[314] Liu Y Yang S Cai E Lin L Zeng P Nie B. Functions of lactate in the brain of rat with intracerebral hemorrhage evaluated with MRI/MRS and *in vitro* approaches. CNS Neurosci Ther. (2020) 26:1031–44. doi: 10.1111/cns.13399 PMC7539841 32488963

[315] Tassinari ID Rodrigues FDS Bertram C Mendes-da-Cruz DA Guedes RP Paz AH. Lactate protects microglia and neurons from oxygen-glucose deprivation/reoxygenation. Neurochem Res. (2024) 49:1762–81. doi: 10.1007/s11064-024-04135-7 38551797

[316] Xiong XY Pan XR Luo XX Wang YF Zhang XX Yang SH. Astrocyte-derived lactate aggravates brain injury of ischemic stroke in mice by promoting the formation of protein lactylation. Theranostics. (2024) 14:4297–317. doi: 10.7150/thno.96375 PMC11303085 39113798

[317] Zhou J Zhang L Peng J Zhang X Zhang F Wu Y. Astrocytic LRP1 enables mitochondria transfer to neurons and mitigates brain ischemic stroke by suppressing ARF1 lactylation. Cell Metab. (2024) 36:2054–2068.e14. doi: 10.1016/j.cmet.2024.05.016 38906140

[318] Zhang Q Wang SS Zhang Z Chu SF. PKM2-mediated metabolic reprogramming of microglia in neuroinflammation. Cell Death Discov. (2025) 11:149. doi: 10.1038/s41420-025-02453-5 40189596 PMC11973174

[319] Kang BS Choi BY Kho AR Lee SH Hong DK Park MK. Effects of pyruvate kinase M2 (PKM2) gene deletion on astrocyte-specific glycolysis and global cerebral ischemia-induced neuronal death. Antioxidants (Basel). (2023) 12:491. doi: 10.3390/antiox12020491 36830049 PMC9952809

[320] Wei Y Miao Q Zhang Q Mao S Li M Xu X. Aerobic glycolysis is the predominant means of glucose metabolism in neuronal somata, which protects against oxidative damage. Nat Neurosci. (2023) 26:2081–89. doi: 10.1038/s41593-023-01476-4 37996529

[321] Xia X Chen W Zhou T Zhou F Lu C Yan Z. TEPP-46 inhibits glycolysis to promote M2 polarization of microglia after ischemic stroke. Int Immunopharmacol. (2025) 149:114148. doi: 10.1016/j.intimp.2025.114148 39904037

[322] Zhu H Zhang H Zhao XJ Zhang L Liu X Zhang ZY. Tetramerization of PKM2 alleviates traumatic brain injury by ameliorating mitochondrial damage in microglia. J Neuroimmune Pharmacol. (2024) 19:48. doi: 10.1007/s11481-024-10138-6 39196455

[323] Xiong XY Liang YJ Zhang XX Yang SH Zhong ZQ Liu SQ. PKM2 nuclear translocation promotes glial cell activation and aggravates the brain injury of intracerebral hemorrhage. J Integr Neurosci. (2023) 22:168. doi: 10.31083/j.jin2206168 38176945

[324] Gao J Liu R Tang J Pan M Zhuang Y Zhang Y. Suppressing nuclear translocation of microglial PKM2 confers neuroprotection via downregulation of neuroinflammation after mouse cerebral ischemia-reperfusion injury. Int Immunopharmacol. (2024) 141:112880. doi: 10.1016/j.intimp.2024.112880 39153304

[325] Li X Zhou R Peng H Peng J Li Q Mei M. Microglia PKM2 mediates neuroinflammation and neuron loss in mice epilepsy through the astrocyte C3-neuron C3R signaling pathway. Brain Sci. (2023) 13:262. doi: 10.3390/brainsci13020262 36831807 PMC9954168

[326] Huang X Guo M Zhang Y Xie J Huang R Zuo Z. Microglial IL-1RA ameliorates brain injury after ischemic stroke by inhibiting astrocytic CXCL1-mediated neutrophil recruitment and microvessel occlusion. Glia. (2023) 71:1607–25. doi: 10.1002/glia.24359 36929654

[327] Song C Zhang Y Dong Y. Acute and subacute IL-1β administrations differentially modulate neuroimmune and neurotrophic systems: possible implications for neuroprotection and neurodegeneration. J Neuroinflammation. (2013) 10:59. doi: 10.1186/1742-2094-10-59 23651534 PMC3656796

[328] Jiang W Wang X Wang W Hua F Zhang Z Zhang Z. Inhibition of NK1R attenuates LPS-induced microglial inflammation and consequent death of PC12 cells. Brain Res Bull. (2020) 162:115–24. doi: 10.1016/j.brainresbull.2020.05.015 32540418

[329] Burmeister AR Johnson MB Chauhan VS Moerdyk-Schauwecker MJ Young AD Cooley ID. Human microglia and astrocytes constitutively express the neurokinin-1 receptor and functionally respond to substance P. J Neuroinflammation. (2017) 14:245. doi: 10.1186/s12974-017-1012-5 29237453 PMC5729418

[330] Jin P Deng S Sherchan P Cui Y Huang L Li G. Neurokinin receptor 1 (NK1R) antagonist aprepitant enhances hematoma clearance by regulating microglial polarization via PKC/p38MAPK/NFκB pathway after experimental intracerebral hemorrhage in mice. Neurotherapeutics. (2021) 18:1922–38. doi: 10.1007/s13311-021-01077-8 PMC8608951 34244927

[331] Jin P Qi D Cui Y Lenahan C Zhang JH Tao X. Aprepitant attenuates NLRC4-dependent neuronal pyroptosis via NK1R/PKCδ pathway in a mouse model of intracerebral hemorrhage. J Neuroinflammation. (2022) 19:198. doi: 10.1186/s12974-022-02558-z 35922848 PMC9351153

[332] Gaire BP Song MR Choi JW. Sphingosine 1-phosphate receptor subtype 3 (S1P3) contributes to brain injury after transient focal cerebral ischemia via modulating microglial activation and their M1 polarization. J Neuroinflammation. (2018) 15:284. doi: 10.1186/s12974-018-1323-1 30305119 PMC6180378

[333] Matuskova H Porschen LT Matthes F Lindgren AG Petzold GC Meissner A. Spatiotemporal sphingosine-1-phosphate receptor 3 expression within the cerebral vasculature after ischemic stroke. iScience. (2024) 27:110031. doi: 10.1016/j.isci.2024.110031 38868192 PMC11167442

[334] Lu L Barfejani AH Qin T Dong Q Ayata C Waeber C. Fingolimod exerts neuroprotective effects in a mouse model of intracerebral hemorrhage. Brain Res. (2014) 1555:89–96. doi: 10.1016/j.brainres.2014.01.048 24502984 PMC3994537

[335] Rolland WB Lekic T Krafft PR Hasegawa Y Altay O Hartman R. Fingolimod reduces cerebral lymphocyte infiltration in experimental models of rodent intracerebral hemorrhage. Exp Neurol. (2013) 241:45–55. doi: 10.1016/j.expneurol.2012.12.009 23261767 PMC3570752

[336] Song D Li M Zhang L Zhang K An Y Feng M. Sphingosine-1-phosphate receptor 3 promotes neuronal apoptosis via the TNF-α/caspase-3 signaling pathway after acute intracerebral hemorrhage. Mol Cell Neurosci. (2024) 131:103979. doi: 10.1016/j.mcn.2024.103979 39581391

[337] Chaudhry SR Shafique S Sajjad S Hänggi D Muhammad S. Janus faced HMGB1 and post-aneurysmal subarachnoid hemorrhage (aSAH) inflammation. Int J Mol Sci. (2022) 23:11216. doi: 10.3390/ijms231911216 36232519 PMC9569479

[338] Wang D Liu K Wake H Teshigawara K Mori S Nishibori M. Anti-high mobility group box-1 (HMGB1) antibody inhibits hemorrhage-induced brain injury and improved neurological deficits in rats. Sci Rep. (2017) 7:46243. doi: 10.1038/srep46243 28393932 PMC5385548

[339] Ieong C Sun H Wang Q Ma J. Glycyrrhizin suppresses the expressions of HMGB1 and ameliorates inflammative effect after acute subarachnoid hemorrhage in rat model. J Clin Neurosci. (2018) 47:278–84. doi: 10.1016/j.jocn.2017.10.034 29078973

[340] Li D Lei C Zhang S Zhang S Liu M Wu B. Blockade of high mobility group box-1 signaling via the receptor for advanced glycation end-products ameliorates inflammatory damage after acute intracerebral hemorrhage. Neurosci Lett. (2015) 609:109–19. doi: 10.1016/j.neulet.2015.10.035 26483322

[341] Yang F Wang Z Zhang JH Tang J Liu X Tan L. Receptor for advanced glycation end-product antagonist reduces blood-brain barrier damage after intracerebral hemorrhage. Stroke. (2015) 46:1328–36. doi: 10.1161/STROKEAHA.114.008336 25782468

[342] Lei C Zhang S Cao T Tao W Liu M Wu B. HMGB1 may act via RAGE to promote angiogenesis in the later phase after intracerebral hemorrhage. Neuroscience. (2022) 481:238–9. doi: 10.1016/j.neuroscience.2021.11.041 34906390

[343] Wang YC Wang PF Fang H Chen J Xiong XY Yang QW. Toll-like receptor 4 antagonist attenuates intracerebral hemorrhage-induced brain injury. Stroke. (2013) 44:2545–52. doi: 10.1161/STROKEAHA.113.001038 23839500

[344] Freitas-Andrade M Comin CH Van Dyken P Ouellette J Raman-Nair J Blakeley N. Astroglial Hmgb1 regulates postnatal astrocyte morphogenesis and cerebrovascular maturation. Nat Commun. (2023) 14:4965. doi: 10.1038/s41467-023-40682-3 37587100 PMC10432480

[345] Hayakawa K Pham LD Katusic ZS Arai K Lo EH. Astrocytic high-mobility group box 1 promotes endothelial progenitor cell-mediated neurovascular remodeling during stroke recovery. Proc Natl Acad Sci U S A. (2012) 109:7505–10. 10.1073/pnas.1121146109 PMC3358881 22529378

[346] Hayakawa K Miyamoto N Seo JH Pham LD Kim KW Lo EH. High-mobility group box 1 from reactive astrocytes enhances the accumulation of endothelial progenitor cells in damaged white matter. J Neurochem. (2013) 125:273–80. doi: 10.1111/jnc.12120 PMC3604050 23227954

[347] Qi L Wang C Deng L Pan JJ Suo Q Wu S. Low-intensity focused ultrasound stimulation promotes stroke recovery via astrocytic HMGB1 and CAMK2N1 in mice. Stroke Vasc Neurol. (2024) 9:505–18. doi: 10.1136/svn-2023-002614 PMC11732924 38191183

[348] Tian X Sun L Feng D Sun Q Dou Y Liu C. HMGB1 promotes neurovascular remodeling via Rage in the late phase of subarachnoid hemorrhage. Brain Res. (2017) 1670:135–45. doi: 10.1016/j.brainres.2017.06.001 28606778

[349] Schaer DJ Vinchi F Ingoglia G Tolosano E Buehler PW. Haptoglobin, hemopexin, and related defense pathways-basic science, clinical perspectives, and drug development. Front Physiol. (2014) 5:415. doi: 10.3389/fphys.2014.00415 25389409 PMC4211382

[350] Bandyopadhyay S Garland P Gaastra B Zolnourian A Bulters D Galea I. The haptoglobin response after aneurysmal subarachnoid haemorrhage. Int J Mol Sci. (2023) 24:16922. doi: 10.3390/ijms242316922 38069244 PMC10707007

[351] Kawakita F Nakajima H Suzuki Y Nampei M Oinaka H Suzuki H. Effects of haptoglobin on early brain injury, vasospasm, and lymphatic drainage after subarachnoid hemorrhage in mice. Stroke. (2024) 55:2885–95. doi: 10.1161/STROKEAHA.124.048048 PMC11593995 39474674

[352] Xu Y Liu Y Wu Y Sun J Lu X Dai K. Curcumin alleviates microglia-mediated neuroinflammation and neuronal ferroptosis following experimental subarachnoid hemorrhage by modulating the nrf2/HO-1 signaling pathway. Mol Neurobiol. (2024). doi: 10.1007/s12035-024-04443-7 39207623

[353] Zhu Q Enkhjargal B Huang L Zhang T Sun C Xie Z. Aggf1 attenuates neuroinflammation and BBB disruption via PI3K/Akt/NF-κB pathway after subarachnoid hemorrhage in rats. J Neuroinflammation. (2018) 15:178. doi: 10.1186/s12974-018-1211-8 29885663 PMC5994242

[354] Wang Y Kong XQ Wu F Xu B Bao DJ Cheng CD. SOCS1/JAK2/STAT3 axis regulates early brain injury induced by subarachnoid hemorrhage via inflammatory responses. Neural Regener Res. (2021) 16:2453–64. doi: 10.4103/1673-5374.313049 PMC8374552 33907034

[355] Zhang L Guo K Zhou J Zhang X Yin S Peng J. Ponesimod protects against neuronal death by suppressing the activation of A1 astrocytes in early brain injury after experimental subarachnoid hemorrhage. J Neurochem. (2021) 158:880–97. doi: 10.1111/jnc.15457 34143505

[356] Sun XG Chu XH Godje Godje IS Liu SY Hu HY Zhang YB. Aerobic Glycolysis Induced by mTOR/HIF-1α Promotes Early Brain Injury After Subarachnoid Hemorrhage via Activating M1 Microglia. Transl Stroke Res. (2024) 15:1–15. doi: 10.1007/s12975-022-01105-5 36385451

[357] Zhang A Cong L Nan C Zhao Z Liu L. 3D biological scaffold delivers Bergenin to reduce neuroinflammation in rats with cerebral hemorrhage. J Transl Med. (2024) 22:946. doi: 10.1186/s12967-024-05735-1 39420402 PMC11484212

[358] You WC Li W Zhuang Z Tang Y Lu HC Ji XJ. Biphasic activation of nuclear factor-kappa B in experimental models of subarachnoid hemorrhage *in vivo* and in *vitro* . Mediators Inflamm. (2012) 2012:786242. doi: 10.1155/2012/786242 23049172 PMC3461645

[359] Tang S Lai N Xu L. Neuronal pyroptosis mediated by STAT3 in early brain injury after subarachnoid hemorrhage. Brain Res. (2024) 1822:148666. doi: 10.1016/j.brainres.2023.148666 37949309

[360] Parker BL Larsen MR Edvinsson LI Povlsen GK. Signal transduction in cerebral arteries after subarachnoid hemorrhage-a phosphoproteomic approach. J Cereb Blood Flow Metab. (2013) 33:1259–69. doi: 10.1038/jcbfm.2013.78 PMC3734778 23715060

[361] Osuka K Watanabe Y Yamauchi K Nakazawa A Usuda N Tokuda M. Activation of the JAK-STAT signaling pathway in the rat basilar artery after subarachnoid hemorrhage. Brain Res. (2006) 1072:1–7. doi: 10.1016/j.brainres.2005.12.003 16413512

[362] Zhang R Yong VW Xue M. Revisiting minocycline in intracerebral hemorrhage: mechanisms and clinical translation. Front Immunol. (2022) 13:844163. doi: 10.3389/fimmu.2022.844163 35401553 PMC8993500

[363] Zheng Y Fan L Xia S Yang Q Zhang Z Chen H. Role of complement C1q/C3-CR3 signaling in brain injury after experimental intracerebral hemorrhage and the effect of minocycline treatment. Front Immunol. (2022) 13:919444. doi: 10.3389/fimmu.2022.919444 36189326 PMC9520460

[364] Yang H Gao X Xiao W Su J Li Y Ni W. Minocycline Alleviates White Matter Injury following Intracerebral Hemorrhage by Regulating CD4+ T Cell Differentiation via Notch1 Signaling Pathway. Oxid Med Cell Longev. (2022) 2022:3435267. doi: 10.1155/2022/3435267 35571238 PMC9098346

[365] Miao H Li R Han C Lu X Zhang H. Minocycline promotes posthemorrhagic neurogenesis via M2 microglia polarization via upregulation of the TrkB/BDNF pathway in rats. J Neurophysiol. (2018) 120:1307–17. doi: 10.1152/jn.00234.2018 29790836

[366] Wang G Li Z Li S Ren J Suresh V Xu D. Minocycline preserves the integrity and permeability of BBB by altering the activity of DKK1-wnt signaling in ICH model. Neuroscience. (2019) 415:135–46. doi: 10.1016/j.neuroscience.2019.06.038 31344398

[367] Zhao F Hua Y He Y Keep RF Xi G. Minocycline-induced attenuation of iron overload and brain injury after experimental intracerebral hemorrhage. Stroke. (2011) 42:3587–93. doi: 10.1161/STROKEAHA.111.623926 PMC3226873 21998050

[368] Zhou K Enkhjargal B Xie Z Sun C Wu L Malaguit J. Dihydrolipoic acid inhibits lysosomal rupture and NLRP3 through lysosome-associated membrane protein-1/calcium/calmodulin-dependent protein kinase II/TAK1 pathways after subarachnoid hemorrhage in rat. Stroke. (2018) 49:175–83. doi: 10.1161/STROKEAHA.117.018593 PMC5744882 29273596

[369] Yuan B Zhou XM You ZQ Xu WD Fan JM Chen SJ. Inhibition of AIM2 inflammasome activation alleviates GSDMD-induced pyroptosis in early brain injury after subarachnoid haemorrhage. Cell Death Dis. (2020) 11:76. doi: 10.1038/s41419-020-2248-z 32001670 PMC6992766

[370] Gan H Zhang L Chen H Xiao H Wang L Zhai X. The pivotal role of the NLRC4 inflammasome in neuroinflammation after intracerebral hemorrhage in rats. Exp Mol Med. (2021) 53:1807–18. doi: 10.1038/s12276-021-00702-y PMC8639719 34848837

[371] Chen S Zuo Y Huang L Sherchan P Zhang J Yu Z. The MC4 receptor agonist RO27-3225 inhibits NLRP1-dependent neuronal pyroptosis via the ASK1/JNK/p38 MAPK pathway in a mouse model of intracerebral haemorrhage. Br J Pharmacol. (2019) 176:1341–56. doi: 10.1111/bph.14639 PMC6468256 30811584

[372] Xiao L Zheng H Li J Zeng M He D Liang J. Targeting NLRP3 inflammasome modulates gut microbiota, attenuates corticospinal tract injury and ameliorates neurobehavioral deficits after intracerebral hemorrhage in mice. BioMed Pharmacother. (2022) 149:112797. 35279596 10.1016/j.biopha.2022.112797

[373] Ren H Kong Y Liu Z Zang D Yang X Wood K. Selective NLRP3 (Pyrin domain-containing protein 3) inflammasome inhibitor reduces brain injury after intracerebral hemorrhage. Stroke. (2018) 49:184–92. doi: 10.1161/STROKEAHA.117.018904 PMC5753818 29212744

[374] Dodd WS Noda I Martinez M Hosaka K Hoh BL. NLRP3 inhibition attenuates early brain injury and delayed cerebral vasospasm after subarachnoid hemorrhage. J Neuroinflammation. (2021) 18:163. doi: 10.1186/s12974-021-02207-x 34284798 PMC8293512

[375] Zhang X Wu Q Zhang Q Lu Y Liu J Li W. Resveratrol Attenuates Early Brain Injury after Experimental Subarachnoid Hemorrhage via Inhibition of NLRP3 Inflammasome Activation. Front Neurosci. (2017) 11:611. doi: 10.3389/fnins.2017.00611 29163015 PMC5675880

[376] Jin L Jin F Guo S Liu W Wei B Fan H. Metformin inhibits NLR family pyrin domain containing 3 (NLRP)-relevant neuroinflammation via an adenosine-5’-monophosphate-activated protein kinase (AMPK)-dependent pathway to alleviate early brain injury after subarachnoid hemorrhage in mice. Front Pharmacol. (2022) 13:796616. doi: 10.3389/fphar.2022.796616 35370693 PMC8969021

[377] Li J Chen J Mo H Chen J Qian C Yan F. Minocycline protects against NLRP3 inflammasome-induced inflammation and P53-associated apoptosis in early brain injury after subarachnoid hemorrhage. Mol Neurobiol. (2016) 53:2668–78. doi: 10.1007/s12035-015-9318-8 26143258

[378] Li X Zhang H Zheng W Sun J Wang L He Z. Ozanimod-dependent activation of SIRT3/NF-κB/AIM2 pathway attenuates secondary injury after intracerebral hemorrhage. Mol Neurobiol. (2023) 60:1117–31. doi: 10.1007/s12035-022-03137-2 36417102

[379] Cai W Dai X Chen J Zhao J Xu M Zhang L. STAT6/Arg1 promotes microglia/macrophage efferocytosis and inflammation resolution in stroke mice. JCI Insight. (2019) 4:e131355. doi: 10.1172/jci.insight.131355 31619589 PMC6824303

[380] Wen L You W Wang H Meng Y Feng J Yang X. Polarization of microglia to the M2 phenotype in a peroxisome proliferator-activated receptor gamma-dependent manner attenuates axonal injury induced by traumatic brain injury in mice. J Neurotrauma. (2018) 35:2330–40. doi: 10.1089/neu.2017.5540 29649924

[381] Soliman E Leonard J Basso EKG Gershenson I Ju J Mills J. Efferocytosis is restricted by axon guidance molecule EphA4 via ERK/Stat6/MERTK signaling following brain injury. J Neuroinflammation. (2023) 20:256. doi: 10.1186/s12974-023-02940-5 37941008 PMC10633953

[382] Huang L Zhang Y Zhao L Chen Q Li L. Ferrostatin-1 polarizes microglial cells toward M2 phenotype to alleviate inflammation after intracerebral hemorrhage. Neurocrit Care. (2022) 36:942–54. doi: 10.1007/s12028-021-01401-2 35099711

[383] Krishna S Cheng B Sharma DR Yadav S Stempinski ES Mamtani S. PPAR-γ activation enhances myelination and neurological recovery in premature rabbits with intraventricular hemorrhage. Proc Natl Acad Sci U S A. (2021) 118:e2103084118. doi: 10.1073/pnas.2103084118 34462350 PMC8433527

[384] Yao X Jiang Q Ding W Yue P Wang J Zhao K. Interleukin 4 inhibits high mobility group box-1 protein-mediated NLRP3 inflammasome formation by activating peroxisome proliferator-activated receptor-γ in astrocytes. Biochem Biophys Res Commun. (2019) 509:624–31. doi: 10.1016/j.bbrc.2018.11.145 30606476

[385] Jang E Kim JH Lee S Kim JH Seo JW Jin M. Phenotypic polarization of activated astrocytes: the critical role of lipocalin-2 in the classical inflammatory activation of astrocytes. J Immunol. (2013) 191:5204–19. doi: 10.4049/jimmunol.1301637 24089194

[386] Quan W Xu CS Li XC Yang C Lan T Wang MY. Telmisartan inhibits microglia-induced neurotoxic A1 astrocyte conversion via PPARγ-mediated NF-κB/p65 degradation. Int Immunopharmacol. (2023) 123:110761. doi: 10.1016/j.intimp.2023.110761 37544025

[387] Apostolakis S Stavrinou P. Pharmacotherapy in SAH: clinical trial lessons. CNS Neurol Disord Drug Targets. (2024) 23:1308–19. doi: 10.2174/0118715273251761231127095039 38243987

[388] Jin J Duan J Du L Xing W Peng X Zhao Q. Inflammation and immune cell abnormalities in intracranial aneurysm subarachnoid hemorrhage (SAH): Relevant signaling pathways and therapeutic strategies. Front Immunol. (2022) 13:1027756. doi: 10.3389/fimmu.2022.1027756 36505409 PMC9727248

[389] Liu H Busl KM Doré S. Role of dexmedetomidine in aneurysmal subarachnoid hemorrhage: A comprehensive scoping review. J Neurosurg Anesthesiol. (2022) 34:176–82. doi: 10.1097/ANA.0000000000000728 33060552

[390] Wang ZJ Lin TH. A competing risk model analysis of dexmedetomidine of in-hospital mortality in subarachnoid hemorrhage patients. Sci Rep. (2024) 14:29590. doi: 10.1038/s41598-024-81025-6 39609577 PMC11604967

[391] Liu Z Yang Y He L Pang M Luo C Liu B. High-dose methylprednisolone for acute traumatic spinal cord injury: A meta-analysis. Neurology. (2019) 93:e841–50. doi: 10.1212/WNL.0000000000007998 31358617

[392] Xu FF Sun S Ho AS Lee D Kiang KM Zhang XQ. Effects of progesterone vs. dexamethasone on brain oedema and inflammatory responses following experimental brain resection. Brain Inj. (2014) 28:1594–601. doi: 10.3109/02699052.2014.943289 25093611

[393] Güresir E Lampmann T Bele S Czabanka M Czorlich P Gempt J. Fight INflammation to Improve outcome after aneurysmal Subarachnoid HEmorRhage (FINISHER) trial: Study protocol for a randomized controlled trial. Int J Stroke. (2023) 18:242–7. doi: 10.1177/17474930221093501 35361026

[394] van Dijk BJ Meijers JCM Kloek AT Knaup VL Rinkel GJE Morgan BP. Complement C5 contributes to brain injury after subarachnoid hemorrhage. Transl Stroke Res. (2020) 11:678–88. doi: 10.1007/s12975-019-00757-0 PMC7340633 31811640

[395] Frank R Szarvas PA Pesti I Zsigmond A Berkecz R Menyhárt Á. Nimodipine inhibits spreading depolarization, ischemic injury, and neuroinflammation in mouse live brain slice preparations. Eur J Pharmacol. (2024) 977:176718. doi: 10.1016/j.ejphar.2024.176718 38849040

[396] Hohmann U Ghadban C Hohmann T Kleine J Schmidt M Scheller C. Nimodipine exerts time-dependent neuroprotective effect after excitotoxical damage in organotypic slice cultures. Int J Mol Sci. (2022) 23:3331. doi: 10.3390/ijms23063331 35328753 PMC8954806

[397] Galea J Ogungbenro K Hulme S Patel H Scarth S Hoadley M. Reduction of inflammation after administration of interleukin-1 receptor antagonist following aneurysmal subarachnoid hemorrhage: results of the Subcutaneous Interleukin-1Ra in SAH (SCIL-SAH) study. J Neurosurg. (2018) 128:515–23. doi: 10.3171/2016.9.JNS16615 28298024

[398] Xu HL Pelligrino DA Paisansathan C Testai FD. Protective role of fingolimod (FTY720) in rats subjected to subarachnoid hemorrhage. J Neuroinflammation. (2015) 12:16. doi: 10.1186/s12974-015-0234-7 25622980 PMC4324852

[399] Wang Y Zhou S Han Z Yin D Luo Y Tian Y. Fingolimod administration improves neurological functions of mice with subarachnoid hemorrhage. Neurosci Lett. (2020) 736:135250. doi: 10.1016/j.neulet.2020.135250 32673690

[400] Feng D Liu T Zhang X Xiang T Su W Quan W. Fingolimod improves diffuse brain injury by promoting AQP4 polarization and functional recovery of the glymphatic system. CNS Neurosci Ther. (2024) 30:e14669. doi: 10.1111/cns.14669 38459666 PMC10924110

[401] Cheng H Di G Gao CC He G Wang X Han YL. FTY720 reduces endothelial cell apoptosis and remodels neurovascular unit after experimental traumatic brain injury. Int J Med Sci. (2021) 18:304–13. doi: 10.7150/ijms.49066 PMC7757143 33390799

[402] Geraghty JR Butler M Maharathi B Tate AJ Lung TJ Balasubramanian G. Diffuse microglial responses and persistent EEG changes correlate with poor neurological outcome in a model of subarachnoid hemorrhage. Sci Rep. (2024) 14:13618. doi: 10.1038/s41598-024-64631-2 38871799 PMC11176397

[403] Fu Y Hao J Zhang N Ren L Sun N Li YJ. Fingolimod for the treatment of intracerebral hemorrhage: a 2-arm proof-of-concept study. JAMA Neurol. (2014) 71:1092–101. doi: 10.1001/jamaneurol.2014.1065 25003359

[404] Li YJ Chang GQ Liu Y Gong Y Yang C Wood K. Fingolimod alters inflammatory mediators and vascular permeability in intracerebral hemorrhage. Neurosci Bull. (2015) 31:755–62. doi: 10.1007/s12264-015-1532-2 PMC5563722 25958190

[405] Kajimoto R Igarashi T Moro N Oshima H Suma T Otani N. Glibenclamide reduces secondary brain injury in a SAH rat model by reducing brain swelling and modulating inflammatory response. J Neurosurg Sci. (2023) 67:431–8. doi: 10.23736/S0390-5616.22.05271-7 35380195

[406] Liu K Zhu J Chang Y Lin Z Shi Z Li X. Attenuation of cerebral edema facilitates recovery of glymphatic system function after status epilepticus. JCI Insight. (2021) 6:e151835. doi: 10.1172/jci.insight.151835 34494549 PMC8492308

[407] Simard JM Chen M Tarasov KV Bhatta S Ivanova S Melnitchenko L. Newly expressed SUR1-regulated NC(Ca-ATP) channel mediates cerebral edema after ischemic stroke. Nat Med. (2006) 12:433–40. doi: 10.1038/nm1390 PMC2740734 16550187

[408] Feng X Zhang T Wang N Qu X Qi M Zhao H. Safety and efficacy of glibenclamide on cerebral oedema following aneurysmal subarachnoid haemorrhage: a randomised, double-blind, placebo-controlled clinical trial. Stroke Vasc Neurol. (2024) 9:530–40. doi: 10.1136/svn-2023-002892 PMC11732842 38191184

[409] Zhao J Yang F Song C Li L Yang X Wang X. Glibenclamide advantage in treating edema after intracerebral hemorrhage (GATE-ICH): study protocol for a multicenter randomized, controlled, assessor-blinded trial. Front Neurol. (2021) 12:656520. doi: 10.3389/fneur.2021.656520 33986719 PMC8110908

[410] Lynch JR Wang H McGirt MJ Floyd J Friedman AH Coon AL. Simvastatin reduces vasospasm after aneurysmal subarachnoid hemorrhage: results of a pilot randomized clinical trial. Stroke. (2005) 36:2024–6. doi: 10.1161/01.STR.0000177879.11607.10 16051891

[411] Chou SH Smith EE Badjatia N Nogueira RG Sims JR Ogilvy CS. A randomized, double-blind, placebo-controlled pilot study of simvastatin in aneurysmal subarachnoid hemorrhage. Stroke. (2008) 39:2891–3. doi: 10.1161/STROKEAHA.107.505875 18658043

[412] Wang Y Chen Q Tan Q Feng Z He Z Tang J. Simvastatin accelerates hematoma resolution after intracerebral hemorrhage in a PPARγ-dependent manner. Neuropharmacology. (2018) 128:244–54. doi: 10.1016/j.neuropharm.2017.10.021 29054366

[413] Li B Mahmood A Lu D Wu H Xiong Y Qu C. Simvastatin attenuates microglial cells and astrocyte activation and decreases interleukin-1beta level after traumatic brain injury. Neurosurgery. (2009) 65:179–86. doi: 10.1227/01.NEU.0000346272.76537.DC PMC2749520 19574840

[414] Chen D Sui L Chen C Liu S Sun X Guan J. Atorvastatin suppresses NLRP3 inflammasome activation in intracerebral hemorrhage via TLR4- and MyD88-dependent pathways. Aging (Albany NY). (2022) 14:462–76. doi: 10.18632/aging.203824 PMC8791214 35017318

[415] Tso MK Macdonald RL. Subarachnoid hemorrhage: a review of experimental studies on the microcirculation and the neurovascular unit. Transl Stroke Res. (2014) 5:174–89. doi: 10.1007/s12975-014-0323-4 24510780

[416] Johshita H Kassell NF Sasaki T. Blood-brain barrier disturbance following subarachnoid hemorrhage in rabbits. Stroke. (1990) 21:1051–8. doi: 10.1161/01.str.21.7.1051 2368106

[417] Germanò A d’Avella D Imperatore C Caruso G Tomasello F. Time-course of blood-brain barrier permeability changes after experimental subarachnoid haemorrhage. Acta Neurochir (Wien). (2000) 142:575–81. doi: 10.1007/s007010050472 10898366

[418] Li Z Liang G Ma T Li J Wang P Liu L. Blood-brain barrier permeability change and regulation mechanism after subarachnoid hemorrhage. Metab Brain Dis. (2015) 30:597–603. doi: 10.1007/s11011-014-9609-1 25270004

[419] Mindt S Tokhi U Hedtke M Groß HJ Hänggi D. Mass spectrometry-based method for quantification of nimodipine and glutamate in cerebrospinal fluid. Pilot study with patients after aneurysmal subarachnoid haemorrhage. J Clin Pharm Ther. (2020) 45:81–7. doi: 10.1111/jcpt.13028 31421063

[420] Dhir N Attri SV Pattanaik S Kumar MP Gill NK Patial A. Aneurysmal subarachnoid hemorrhage: impact on phenytoin permeability across the blood-brain barrier. Neurol India. (2020) 68:588–92. doi: 10.4103/0028-3886.288987 32643669

[421] Porchet F Chioléro R de Tribolet N. Hypotensive effect of nimodipine during treatment for aneurysmal subarachnoid haemorrhage. Acta Neurochir (Wien). (1995) 137:62–9. doi: 10.1007/BF02188783 8748871

[422] Luzzi S Bektaşoğlu PK Doğruel Y Güngor A. Beyond nimodipine: advanced neuroprotection strategies for aneurysmal subarachnoid hemorrhage vasospasm and delayed cerebral ischemia. Neurosurg Rev. (2024) 47:305. doi: 10.1007/s10143-024-02543-5 38967704 PMC11226492

[423] Stiefel MF Heuer GG Abrahams JM Bloom S Smith MJ Maloney-Wilensky E. The effect of nimodipine on cerebral oxygenation in patients with poor-grade subarachnoid hemorrhage. J Neurosurg. (2004) 101:594–9. doi: 10.3171/jns.2004.101.4.0594 15481712

[424] Viderman D Sarria-Santamera A Bilotta F. Side effects of continuous intra-arterial infusion of nimodipine for management of resistant cerebral vasospasm in subarachnoid hemorrhage patients: A systematic review. Neurochirurgie. (2021) 67:461–9. doi: 10.1016/j.neuchi.2021.02.005 33652066

[425] Haley EC Jr Kassell NF Torner JC. A randomized trial of nicardipine in subarachnoid hemorrhage: angiographic and transcranial Doppler ultrasound results. A report of the Cooperative Aneurysm Study. J Neurosurg. (1993) 78:548–53. doi: 10.3171/jns.1993.78.4.0548 8450327

[426] Shan T Zhang T Qian W Ma L Li H You C. Effectiveness and feasibility of cilostazol in patients with aneurysmal subarachnoid hemorrhage: a systematic review and meta-analysis. J Neurol. (2020) 267:1577–84. doi: 10.1007/s00415-019-09198-z 30739182

[427] Wessels L Wolf S Adage T Breitenbach J Thomé C Kerschbaumer J. Localized nicardipine release implants for prevention of vasospasm after aneurysmal subarachnoid hemorrhage: A randomized clinical trial. JAMA Neurol. (2024) 81:1060–5. doi: 10.1001/jamaneurol.2024.2564 PMC11334004 39158893

[428] Sweeney JF Chen J Darwish B Holden D Barnes E Varelas P. Intrathecal nicardipine after aneurysmal subarachnoid hemorrhage: A scoping review. Neurocrit Care. (2025) 42:595–609. doi: 10.1007/s12028-024-02175-z 39715986

[429] Chen G Cao Y Du X Cui J Zeng X Yang H. The clinical research landscape of intracranial nicardipine for aneurysmal subarachnoid hemorrhage: insights from bibliometric analysis. Drug Des Devel Ther. (2025) 19:1129–46. doi: 10.2147/DDDT.S503226 PMC11847436 39991082

[430] Sun Q Xu X Wang T Xu Z Lu X Li X. Neurovascular units and neural-glia networks in intracerebral hemorrhage: from mechanisms to translation. Transl Stroke Res. (2021) 12:447–60. doi: 10.1007/s12975-021-00897-2 33629275

[431] Thilak S Brown P Whitehouse T Gautam N Lawrence E Ahmed Z. Diagnosis and management of subarachnoid haemorrhage. Nat Commun. (2024) 15:1850. doi: 10.1038/s41467-024-46015-2 38424037 PMC10904840

[432] Song Q Ruiz J Xing F Lo HW Craddock L Pullikuth AK. Single-cell sequencing reveals the landscape of the human brain metastatic microenvironment. Commun Biol. (2023) 6:760. doi: 10.1038/s42003-023-05124-2 37479733 PMC10362065

[433] Soelter TM Howton TC Clark AD Oza VH Lasseigne BN. Altered glia-neuron communication in Alzheimer’s Disease affects WNT, p53, and NFkB Signaling determined by snRNA-seq. Cell Commun Signal. (2024) 22:317. doi: 10.1186/s12964-024-01686-8 38849813 PMC11157763

[434] Wälchli T Ghobrial M Schwab M. Single-cell atlas of the human brain vasculature across development, adulthood and disease. Nature. (2024) 632:603–13. 10.1038/s41586-024-07493-y PMC11324530 38987604

[435] Wang X Wen D Xia F Fang M Zheng J You C. Single-cell transcriptomics revealed white matter repair following subarachnoid hemorrhage. Transl Stroke Res. (2024). doi: 10.1007/s12975-024-01265-6 38861152

[436] Zhang P Gao C Guo Q Yang D Zhang G Lu H. Single-cell RNA sequencing reveals the evolution of the immune landscape during perihematomal edema progression after intracerebral hemorrhage. J Neuroinflammation. (2024) 21:140. doi: 10.1186/s12974-024-03113-8 38807233 PMC11131315

[437] Hirano K Hirano M. Current perspective on the role of the thrombin receptor in cerebral vasospasm after subarachnoid hemorrhage. J Pharmacol Sci. (2010) 114:127–33. doi: 10.1254/jphs.10R03CP 20859063

[438] Romoli M Giammello F Mosconi MG De Mase A De Marco G Digiovanni A. Immunological profile of vasospasm after subarachnoid hemorrhage. Int J Mol Sci. (2023) 24:8856. doi: 10.3390/ijms24108856 37240207 PMC10218712

[439] Jackson CM Choi J Routkevitch D Pant A Saleh L Ye X. PD-1+ Monocytes mediate cerebral vasospasm following subarachnoid hemorrhage. Neurosurgery. (2021) 88:855–63. doi: 10.1093/neuros/nyaa495 33370819

[440] Kubota T Handa Y Tsuchida A Kaneko M Kobayashi H Kubota T. The kinetics of lymphocyte subsets and macrophages in subarachnoid space after subarachnoid hemorrhage in rats. Stroke. (1993) 24:1993–2001. doi: 10.1161/01.str.24.12.1993 8248982

[441] Guo Y Liu J Zeng H Cai L Wang T Wu X. Neutrophil to lymphocyte ratio predicting poor outcome after aneurysmal subarachnoid hemorrhage: A retrospective study and updated meta-analysis. Front Immunol. (2022) 13:962760. doi: 10.3389/fimmu.2022.962760 36016932 PMC9398491

[442] Gris T Laplante P Thebault P Cayrol R Najjar A Joannette-Pilon B. Innate immunity activation in the early brain injury period following subarachnoid hemorrhage. J Neuroinflammation. (2019) 16:253. doi: 10.1186/s12974-019-1629-7 31801576 PMC6894125

[443] Roa JA Sarkar D Zanaty M Ishii D Lu Y Karandikar NJ. Preliminary results in the analysis of the immune response after aneurysmal subarachnoid hemorrhage. Sci Rep. (2020) 10:11809. doi: 10.1038/s41598-020-68861-y 32678268 PMC7367262

[444] Tao T Chen X Zhou Y Huang ZJ Rong YY Lin QS. Continued P2X7 activation leads to mitochondrial fission and compromising microglial phagocytosis after subarachnoid haemorrhage. J Neurochem. (2022) 163:419–37. doi: 10.1111/jnc.15712 PMC9828135 36269673

[445] Chen P Lin MH Li YX Huang ZJ Rong YY Lin QS. Bexarotene enhances astrocyte phagocytosis via ABCA1-mediated pathways in a mouse model of subarachnoid hemorrhage. Exp Neurol. (2024) 378:114839. doi: 10.1016/j.expneurol.2024.114839 38824081

[446] Nijboer CH Kooijman E van Velthoven CT. Intranasal stem cell treatment as a novel therapy for subarachnoid hemorrhage. Stem Cells Dev. (2018) 27:313–25. doi: 10.1089/scd.2017.0148 29310519

[447] Sankarappan K Shetty AK. Promise of mesenchymal stem cell-derived extracellular vesicles for alleviating subarachnoid hemorrhage-induced brain dysfunction by neuroprotective and antiinflammatory effects. Brain Behav Immun Health. (2024) 40:100835. doi: 10.1016/j.bbih.2024.100835 39165307 PMC11334735

[448] Liu W Li R Yin J Guo S Chen Y Fan H. Mesenchymal stem cells alleviate the early brain injury of subarachnoid hemorrhage partly by suppression of Notch1-dependent neuroinflammation: involvement of Botch. J Neuroinflammation. (2019) 16:8. doi: 10.1186/s12974-019-1396-5 30646897 PMC6334441

[449] Li J Wang H Du C Jin X Geng Y Han B. hUC-MSCs ameliorated CUMS-induced depression by modulating complement C3 signaling-mediated microglial polarization during astrocyte-microglia crosstalk. Brain Res Bull. (2020) 163:109–19. doi: 10.1016/j.brainresbull.2020.07.004 32681971

[450] Baranovskii DS Klabukov ID Arguchinskaya NV Yakimova AO Kisel AA Yatsenko EM. Adverse events, side effects and complications in mesenchymal stromal cell-based therapies. Stem Cell Investig. (2022) 9:7. doi: 10.21037/sci-2022-025 PMC9659480 36393919

[451] Zhu X Badawi M Pomeroy S Sutaria DS Xie Z Baek A. Comprehensive toxicity and immunogenicity studies reveal minimal effects in mice following sustained dosing of extracellular vesicles derived from HEK293T cells. J Extracell Vesicles. (2017) 6:1324730. doi: 10.1080/20013078.2017.1324730 28717420 PMC5505007

[452] Yang M Deng S Jiang J Tian M Xiao L Gong Y. Oxytocin improves intracerebral hemorrhage outcomes by suppressing neuronal pyroptosis and mitochondrial fission. Stroke. (2023) 54:1888–900. doi: 10.1161/STROKEAHA.123.043391 37317879

[453] Fan H Ding R Liu W Zhang X Li R Wei B. Heat shock protein 22 modulates NRF1/TFAM-dependent mitochondrial biogenesis and DRP1-sparked mitochondrial apoptosis through AMPK-PGC1α signaling pathway to alleviate the early brain injury of subarachnoid hemorrhage in rats. Redox Biol. (2021) 40:101856. doi: 10.1016/j.redox.2021.101856 33472123 PMC7816003

[454] Zhang Z Zhang A Liu Y Hu X Fang Y Wang X. New mechanisms and targets of subarachnoid hemorrhage: A focus on mitochondria. Curr Neuropharmacol. (2022) 20:1278–96. doi: 10.2174/1570159X19666211101103646 PMC9881073 34720082

[455] Chen W Huang J Hu Y Khoshnam SE Sarkaki A. Mitochondrial transfer as a therapeutic strategy against ischemic stroke. Transl Stroke Res. (2020) 11:1214–28. doi: 10.1007/s12975-020-00828-7 32592024

[456] Hayakawa K Esposito E Wang X Terasaki Y Liu Y Xing C. Transfer of mitochondria from astrocytes to neurons after stroke. Nature. (2016) 539:123. doi: 10.1038/nature19805. *Nature.* 2016;535(7613):551-555. PMC4968589 27466127

[457] Scheiblich H Eikens F Wischhof L Opitz S Jüngling K Cserép C. Microglia rescue neurons from aggregate-induced neuronal dysfunction and death through tunneling nanotubes. Neuron. (2024) 112:3106–3125.e8. doi: 10.1016/j.neuron.2024.06.029 39059388

[458] Jung JE Sun G Bautista Garrido J Obertas L Mobley AS Ting SM. The mitochondria-derived peptide humanin improves recovery from intracerebral hemorrhage: implication of mitochondria transfer and microglia phenotype change. J Neurosci. (2020) 40:2154–65. doi: 10.1523/JNEUROSCI.2212-19.2020 PMC7055138 31980585

[459] Tashiro R Bautista-Garrido J Ozaki D Sun G Obertas L Mobley AS. Transplantation of astrocytic mitochondria modulates neuronal antioxidant defense and neuroplasticity and promotes functional recovery after intracerebral hemorrhage. J Neurosci. (2022) 42:7001–14. doi: 10.1523/JNEUROSCI.2222-21.2022 PMC9463988 35970559

